# Synaptic and intrinsic plasticity mediated by CCK-type signaling coordinates behavioral changes during motivational state shifts

**DOI:** 10.1016/j.celrep.2025.116049

**Published:** 2025-07-22

**Authors:** Guo Zhang, Xue-Ying Ding, Elena V. Romanova, Cui-Ping Liu, Michael A. Barry, Alisha Doda, Qian-Xue Chen, Carrie Reaver, Qing-Chun Jin, Stanislav S. Rubakhin, Fan Li, Yu-Fei Jin, Yan-Sheng Kan, Yu-Ling Liu, Shi-Qi Guo, Ying-Yu Xue, Yu-Shuo Mei, Ping Fu, Ju-Ping Xu, Rui-Ting Mao, Cheng-Yi Liu, Yan-Chu-Fei Zhang, Yi-Long Zhang, Jian-Hui Chang, Shao-Qian Wu, Hui-Ying Wang, Wei-Jia Liu, Ping Chen, Zhen Zhou, Hai-Bo Zhou, Quan Yu, James W. Checco, Jonathan V. Sweedler, Elizabeth C. Cropper, Jian Jing

**Affiliations:** 1 State Key Laboratory of Pharmaceutical Biotechnology, Department of Neurology and Medical Psychology, Nanjing Drum Tower Hospital, The Affiliated Hospital of Nanjing University Medical School, Institute for Brain Sciences, School of Life Sciences, Nanjing University, Nanjing, Jiangsu, China; 2 Department of Chemistry and the Beckman Institute for Advanced Science and Technology, University of Illinois at Urbana-Champaign, Urbana, IL, USA; 3 Department of Neuroscience and Friedman Brain Institute, Icahn School of Medicine at Mount Sinai, New York, NY, USA; 4 Department of Chemistry, University of Nebraska-Lincoln, Lincoln, NE, USA; 5 School of Electronic Science and Engineering, Nanjing University, Nanjing, Jiangsu, China; 6 Peng Cheng Laboratory, Shenzhen, China; 7 The Nebraska Center for Integrated Biomolecular Communication (NCIBC), University of Nebraska-Lincoln, Lincoln, NE, USA; 8 These authors contributed equally; 9 Lead contact

## Abstract

Transitions from hunger to satiety involve multiple behavioral changes, including modulation and inhibition of feeding behavior. In mammals, cholecystokinin (CCK) is a key satiety peptide implicated in these processes; however, whether and how CCK might induce satiety via synaptic and intrinsic plasticity remains unclear. Here, we investigate CCK-type signaling in the protostome mollusk *Aplysia californica*. We demonstrate that *Aplysia* CCK (apCCK) acts as a conserved brain-gut peptide. Gut-localized apCCK-expressing neurons project centrally and release apCCK near the feeding-pattern generator. *In vivo*, apCCK suppresses food intake, while *in vitro*, it shifts motor output toward egestive patterns and inhibits feeding programs. Mechanistically, apCCK modulates the excitability of the egestive-promoting B20 interneuron and suppresses synaptic input to protraction-phase motoneurons, thereby altering program selection and inhibiting feeding-program generation. These findings highlight the importance of both synaptic and intrinsic plasticity in specific circuit elements for implementing motivational shifts driven by satiety signaling.

## In brief

Zhang et al. employed multidisciplinary approaches to comprehensively characterize cholecystokinin (CCK)-type signaling in the nervous system and gut of the mollusk *Aplysia californica*, revealing that CCK functions as a brain-gut peptide regulating satiety, analogous to its role in mammals. Tellingly, these effects are mediated through both synaptic and intrinsic plasticity mechanisms.

## Graphical Abstract

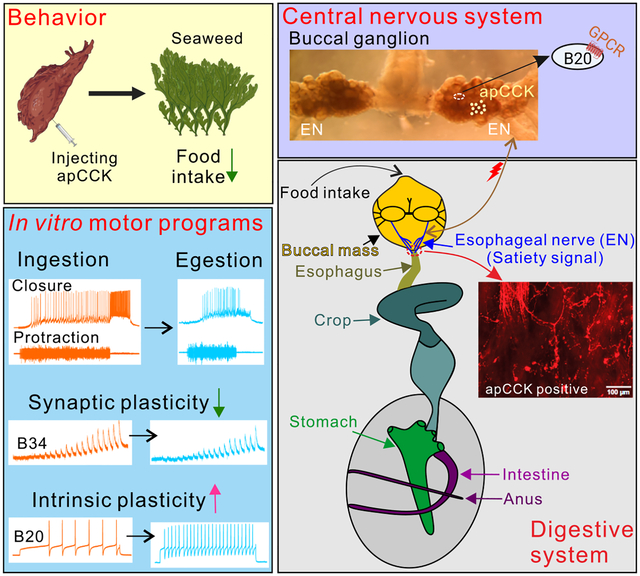

## INTRODUCTION

An animal’s behavior is dynamically shaped by its motivational state, sensory input, and prior learning experience. A defining feature of motivational states is their capacity to organize behavior into coherent, goal-directed sequences by inducing multiple coordinated behavioral changes.^1–7^ One of the most fundamental motivational shifts occurs between hunger and satiety, involving complex changes in feeding behavior. During this transition, the behavior changes in that ingestive responses are replaced by rejection responses, and, eventually, feeding stops.^8–14^ The neural and molecular mechanisms driving these coordinated behavioral changes remain an active area of investigation.

Satiety peptides play a pivotal role in the transition from hunger to satiation.^14–22^ Among these, cholecystokinin (CCK) has been extensively studied as a key neuropeptide regulating satiety. First identified as a satiety signal in mammals,^23,24^ CCK has since been implicated in feeding regulation across various species.^25–28^ Its release has been associated with reduced food intake and increased satiety.^20,24,28–33^ Despite substantial research, the precise neural circuits and mechanisms by which CCK establishes a satiety-driven motivational state remains elusive. In particular, while peptides are known to modulate motor circuits through synaptic or intrinsic plasticity,^3,34–37^ it remains unclear how such plasticity orchestrates multiple behavioral changes. For instance, in mammals, while postprandial activation of gut CCK cells stimulates the vagus afferents, which in turn activates neurons in the nucleus tractus solitarius (NTS),^32^ the downstream effects on brainstem pattern-generating circuits to regulate feeding remain uncertain. In arthropods, CCK-type peptides, known as sulfakinins (SKs), have been extensively studied in the context of feeding, mating, and aggression.^33,38–44^ SK signaling reduces food intake,^42,43,45,46^ but the absence of gut SKs in most species^43,45,47–49^ indicates that they do not function as satiety peptides in these organisms.

To address these gaps, we investigated the CCK signaling system in the mollusk *Aplysia californica* (*Aplysia*), a model organism ideal for studying neural mechanisms due to its relatively simple nervous system, well-characterized feeding circuit, and established roles of modulatory peptides.^13,36,50–66^ This model allows a unique exploration of whether *Aplysia* possesses gut CCK cells and how they influence the central nervous system (CNS) circuit to coordinate behavioral changes.

In this study, we characterized the *Aplysia* CCK (apCCK) precursor and its constituent peptides using *in situ* hybridization, immunohistochemistry, and mass spectrometry (MS). We identified multiple apCCK forms localized in the CNS and the gut, four of which suppressed food intake. We characterized two *Aplysia* CCK receptors, apCCKR1 and apCCKR2, and their differential activation by various apCCKs. Importantly, gut apCCK neurons project to the buccal ganglion via the esophageal nerve (EN), which innervates the gut. EN stimulation triggers apCCK release in the buccal ganglion. As the buccal ganglion houses the feeding-pattern generator, these data suggest a direct role for apCCK in modulating motor circuits. *In vitro*, apCCKs bias motor programs toward egestion and inhibit feeding-motor programs by modulating the excitability of specific interneurons and suppressing direct and indirect excitatory inputs to protraction motoneurons, respectively. Single-cell RNA sequencing using switching mechanism at 5^′^ end of RNA template (SMART) technology (SMART-seq) revealed the expression of the apCCK receptors in individual interneurons, providing insights into apCCK-mediated modulation.

Our study provides comprehensive insights into apCCK-mediated circuit and neural mechanisms, highlighting how both synaptic and intrinsic plasticity coordinate behavioral changes during motivational-state shifts. It supports the evolutionary conservation of satiety signaling pathways and underscores the value of comparative models in uncovering fundamental principles of neural circuit modulation.

## RESULTS

### Identification of the CCK precursor in *Aplysia*

To identify the CCK precursor in *Aplysia*, we conducted BLAST searches in the NCBI database and AplysiaTools (http://aplysiatools.org/).^67^ Both searches returned the same protein with similar mRNA sequences (Figure S1). To verify the transcript’s presence in *Aplysia*, we designed primers based on the putative sequence (Data S1) and performed PCR on cDNA from the *Aplysia* CNS. We obtained a 459-bp mRNA (Figure S2A), now deposited in the NCBI database (NCBI: PV530374). Phylogenetic analysis showed that it is most closely related to CCK precursors in other mollusks, such as *Crassostrea gigas* (21.56% similarity, Figure S3 and Data S2).

NeuroPred^68^ analysis predicted two CCK-like peptides: apCCK1 (QGAWSYDYGLGGGRF-NH_2_) and apCCK2 (SYGDYGI GGGRF-NH_2_) (Figure S4). Most CCKs in both protostomes and deuterostomes contain at least one conserved tyrosine (Tyr), which can be sulfated and is critical for peptide activities.^69–71^ In *Aplysia*, apCCK1 and apCCK2 contain two tyrosine residues, potentially generating eight variants, including non-sulfated, monosulfated, and disulfated forms (Figure S4B). Sequence comparisons revealed structural similarities with other species’ CCKs or arthropods’ SKs,^27,45,70^ including amidation and a conserved C-terminal residue (Figure S4 and Data S3).

These findings confirm the presence of an apCCK precursor and suggest the existence of eight possible apCCK isoforms.

### Distribution of apCCK precursor mRNA and peptides in *Aplysia* tissues

**Figure 1.**
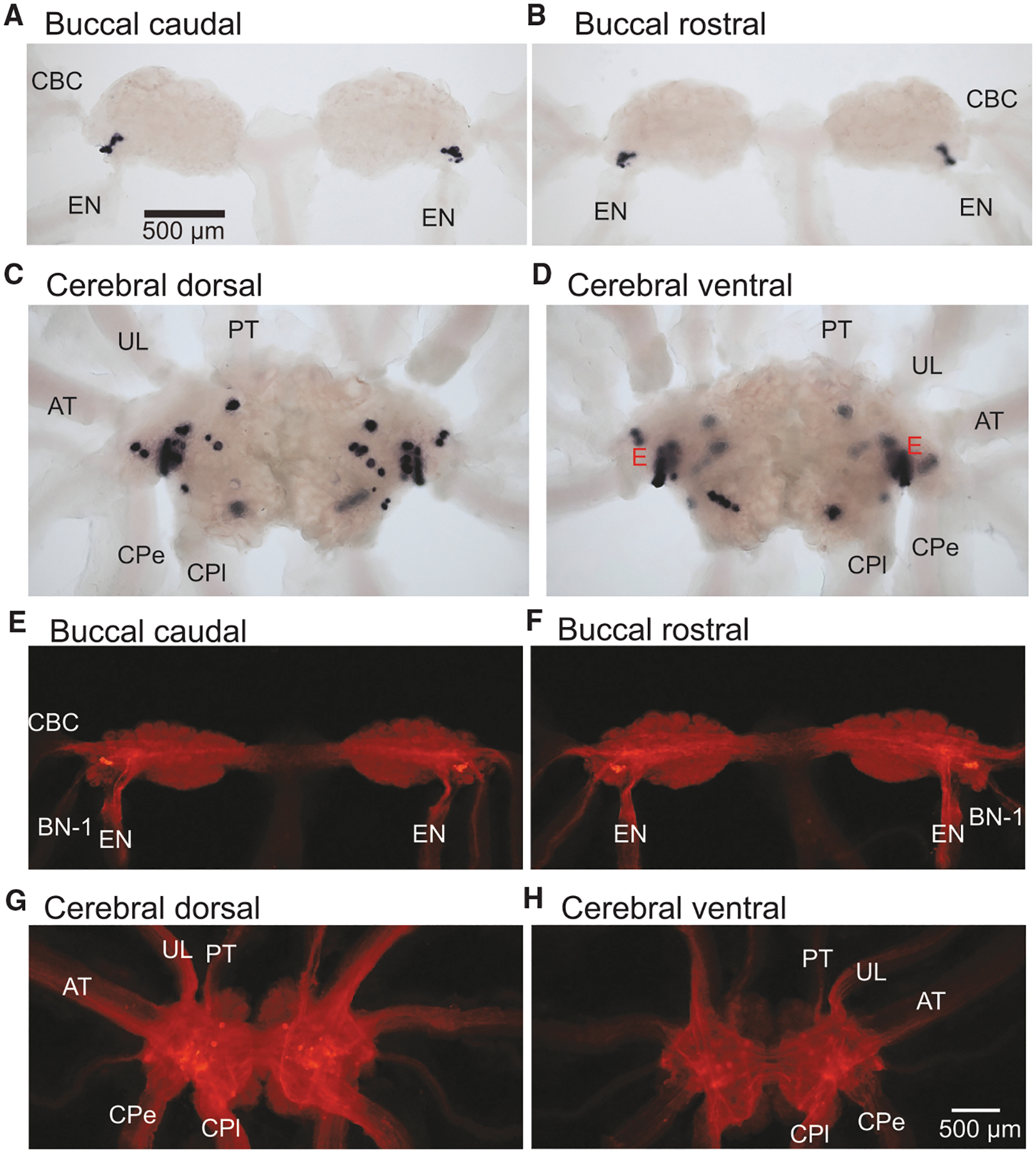
Distribution of apCCK-positive neurons in the buccal and cerebral ganglia in whole mounts by *in situ* hybridization and immunohistochemistry (A–D) *In situ* hybridization. The caudal (A) and rostral (B) surface of buccal ganglia. Note the positive neurons near the root of EN/BN-1 in the buccal caudal surface. The dorsal (C) and ventral (D) surface of cerebral ganglia. Note the positive E cluster neurons on the cerebral dorsal and ventral surface. (E–H) Immunohistochemistry.The caudal (E) and rostral (F) surface of buccal ganglia. The dorsal (G) and ventral (H) surface of cerebral ganglia. (A–D) have the same magnification; (E–H) have the same magnification. *n* = 3 ganglia for each panel. Buccal abbreviations: BN-1, buccal nerve 1; EN, esophageal nerve; CBC, cerebral-buccal connective nerve. Cerebral abbreviations: UL, upper labial nerve; PT, posterior tentacular nerve; AT, anterior tentacular nerve; CPe, cerebral-pedal connective nerve; CPl, cerebral-pleural connective nerve. See also Figure S6.

Analysis of *Aplysia* mRNA expression data (GEO: GSE79231) (Figure S5) showed that the apCCK precursor is present in both the CNS and peripheral tissues. To map apCCK-positive neurons in the CNS of *Aplysia*, we used *in situ* hybridization on whole mounts that revealed apCCK expression in all central ganglia, including the buccal ganglion (Figures 1A and 1B), the cerebral ganglion (Figures 1C and 1D), the abdominal ganglion (Figures S6A and S6B), and the pleural-pedal ganglia (Figures S6C–S6F), with the cerebral ganglion containing the highest number of apCCK-positive neurons, especially in the “E” cluster. The buccal ganglion had a cluster on the caudal surface, while the abdominal and pleural-pedal ganglia had fewer dispersed neurons. Immunohistochemistry showed similar patterns of cell-body staining (Figures 1E–1H and S6G–S6L). In addition, immunohistochemistry revealed various degrees of apCCK immunoreactivity (apCCK-IR) in the nerves of all major ganglia, including all nerves connected to the buccal ganglion and the CBC (cerebral buccal connective) (Figures 1E and 1F), which could not be stained by *in situ* hybridization due to the lack of mRNAs in nerves.

### Identification of mature apCCKs in *Aplysia* by MS

To characterize the apCCK precursor processing, we utilized liquid chromatography-tandem MS (LC-MS/MS) to analyze mature peptides from central ganglia extracts. We identified amidated peptides with and without N-terminal pyroglutamylation, along with various sulfation states. These included non-sulfated apCCK1 and apCCK2 (Figure S7 and Data S4) as well as five sulfated forms: [sY6]-apCCK1, [sY8]-apCCK1, [sY6, sY8]-apCCK1, [sY2]-apCCK2, and [sY5]-apCCK2 (Figure S8 and Data S4). Disulfated CCK2 ([sY2, sY5]-apCCK2) was not detected, likely due, at least in part, to its low abundance.

### apCCKs significantly reduce food intake in intact animals

**Figure 2.**
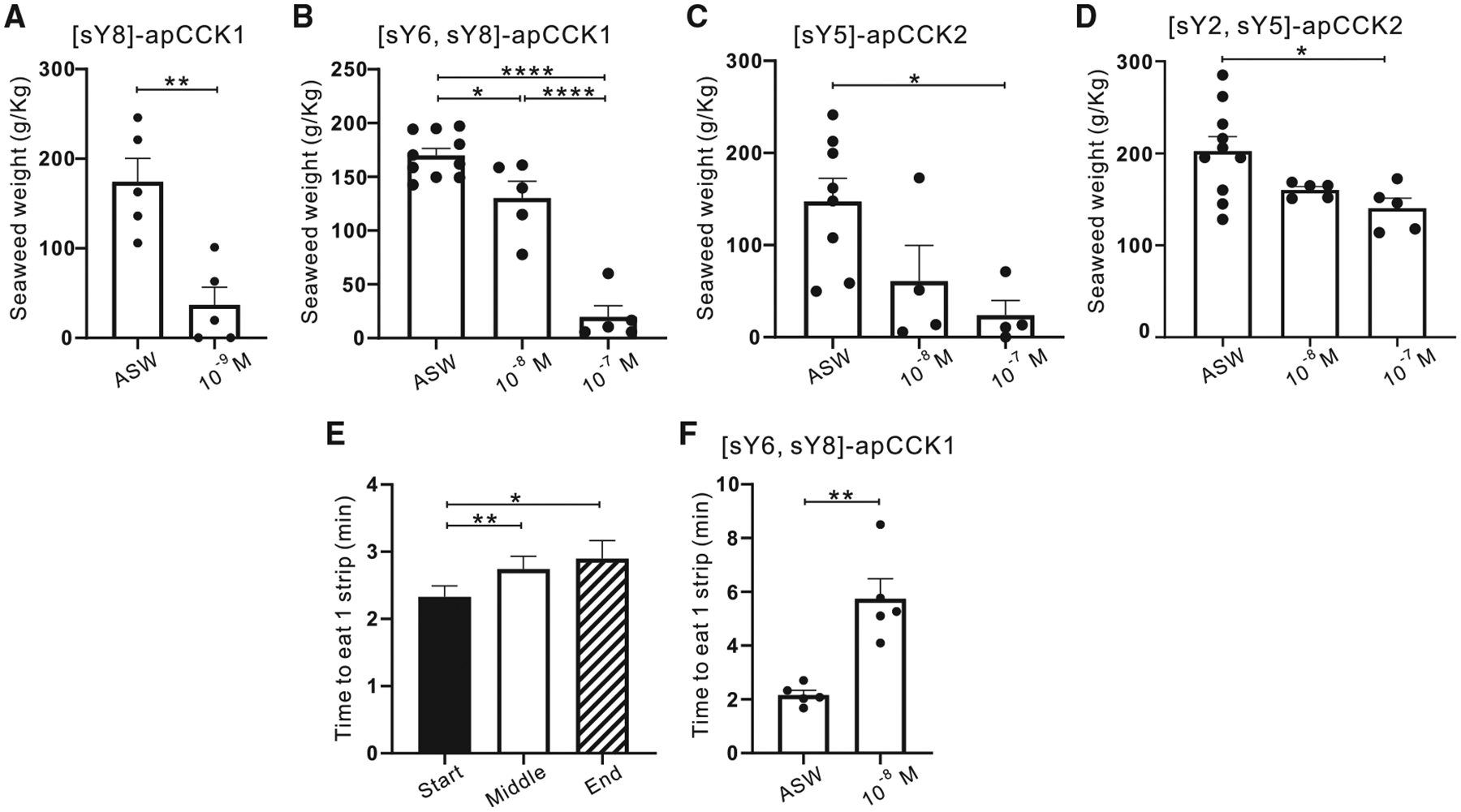
Effects of apCCKs on feeding behavior (A–D) Monosulfated [sY8]-apCCK1 (A, paired t test, *t*_4_ = 5.979, *p* = 0.0039), disulfated [sY6, sY8]-apCCK1 (B, *F*(2, 17) = 59.52, *p* < 0.0001), monosulfated [sY5]-apCCK2 (C, *F*(2, 13) = 5.449, *p* = 0.0191), and disulfated [sY2, sY5]-apCCK2 (D, *F*(2, 17) = 4.995, *p* = 0.0197) reduced the amount of food (seaweed) consumed in a single meal in a dose-dependent manner. (E) As the meal progressed from the start, through the middle, to the end periods, control animals took longer to consume single strips of seaweed (1 × 10 cm). *F*(2, 48) = 6.11, *p* = 0.0071, *n* = 25. (F) Animals injected with 10^−8^ M [sY6, sY8]-apCCK1 took longer to consume one strip of seaweed (average of the first three strips) compared with control animals. Paired t test, *t*_4_ = 4.651, *p* = 0.0097. One-way ANOVAs are used in (B)–(E) with their Tukey post hoc tests: *p < 0.05, **p < 0.01, ****p < 0.0001. Error bars denote SEM. See also Figure S9.

To explore the role of apCCKs as satiety signals in mollusks, we examined their effects on food intake. Based on their presence in feeding-related ganglia, animals were injected with artificial seawater (ASW) or apCCK solutions (10^−7^ M or 10^−6^ M). If the 10^−7^ M concentration completely inhibited feeding behaviors, subsequent experiments used lower concentrations. Four of eight apCCK forms significantly reduced food intake (Figures 2A–2D). [sY8]-apCCK1 was the most potent (Figure 2A), completely inhibiting consumption at 10^−8^ M or 10^−7^ M. [sY6, sY8]-apCCK1 also strongly reduced food intake (Figure 2B), while [sY5]-apCCK2 (Figure 2C) and [sY2, sY5]-apCCK2 (Figure 2D) showed significant but weaker effects. The remaining four forms had no impact (Figure S9). Moreover, [sY6, sY8]-apCCK1 slowed food consumption (Figure 2E), which could stem, in part, from a shift from ingestive to egestive patterns as the meal progressed. [sY6, sY8]-apCCK1 also extended the duration to consume one strip of seaweed compared to controls (Figure 2F), further mimicking natural satiety.

These findings indicate that specific sulfated apCCK forms act as satiety peptides in *Aplysia*.

### Identification of apCCK receptors: The molecular mechanisms underlying the action of apCCK

Next, we sought to identify putative apCCK receptors. First, we searched the NCBI database and AplysiaTools and obtained four putative apCCK receptors (Figures S10–S13 and Data S5). BLAST searches of each sequence against NCBI in species with well-characterized protein sequences (Data S6) suggest that two sequences are potential apCCK receptors. We tentatively named these sequences apCCKR1 (Figure S10) and apCCKR2 (Figure S11) (sequences deposited into the NCBI database with accession numbers NCBI: PV530375 and PV530376). The other two sequences were named Class-A_GPCR1 (Figure S12) and Srw_GPCR1 (Figure S13), respectively.

Phylogenetic analysis based on a balanced tree from Jiang et al.^62^ after adding CCK receptors from molluscan species showed that apCCKR1 and apCCKR2 clustered with *Lottia gigantea* CCK3R and CCK2R (Figure S14B), whereas the other two sequences did not. Another tree with CCKRs/SKRs, neuropeptide Y (NPY)-Rs/neuropeptide F (NPF)-Rs, orexin/allatotropin-Rs, and vasopressin/oxytocin-Rs families from various species (Figure S15) indicates that all CCKRs/SKRs, including molluscan ones, form one clade, supporting a conserved lineage of CCK receptors across bilaterians. Furthermore, this analysis indicates that CCK receptors are more closely related to NPY receptors than to orexin/allatotropin or vasopressin/oxytocin receptors. Finally, it indicates that apCCKR1 is most closely related to *Lottia gigantea* CCK2R (similarity 50.59%) and that apCCKR2 is more similar to *Lottia gigantea* CCK3R (similarity 61.81%) (Figures S15 and S16; Data S7).

We cloned these four receptors (Figures S2B–S2E) using primers (Data S1) designed on the basis of their open reading frame sequences. To search for additional sequences that might be related to apCCKRs, we used the cloned apCCKRs’ sequences to BLAST both the transcriptome and the genome of the AplysiaTools databases but found no more related sequences.

**Figure 3.**
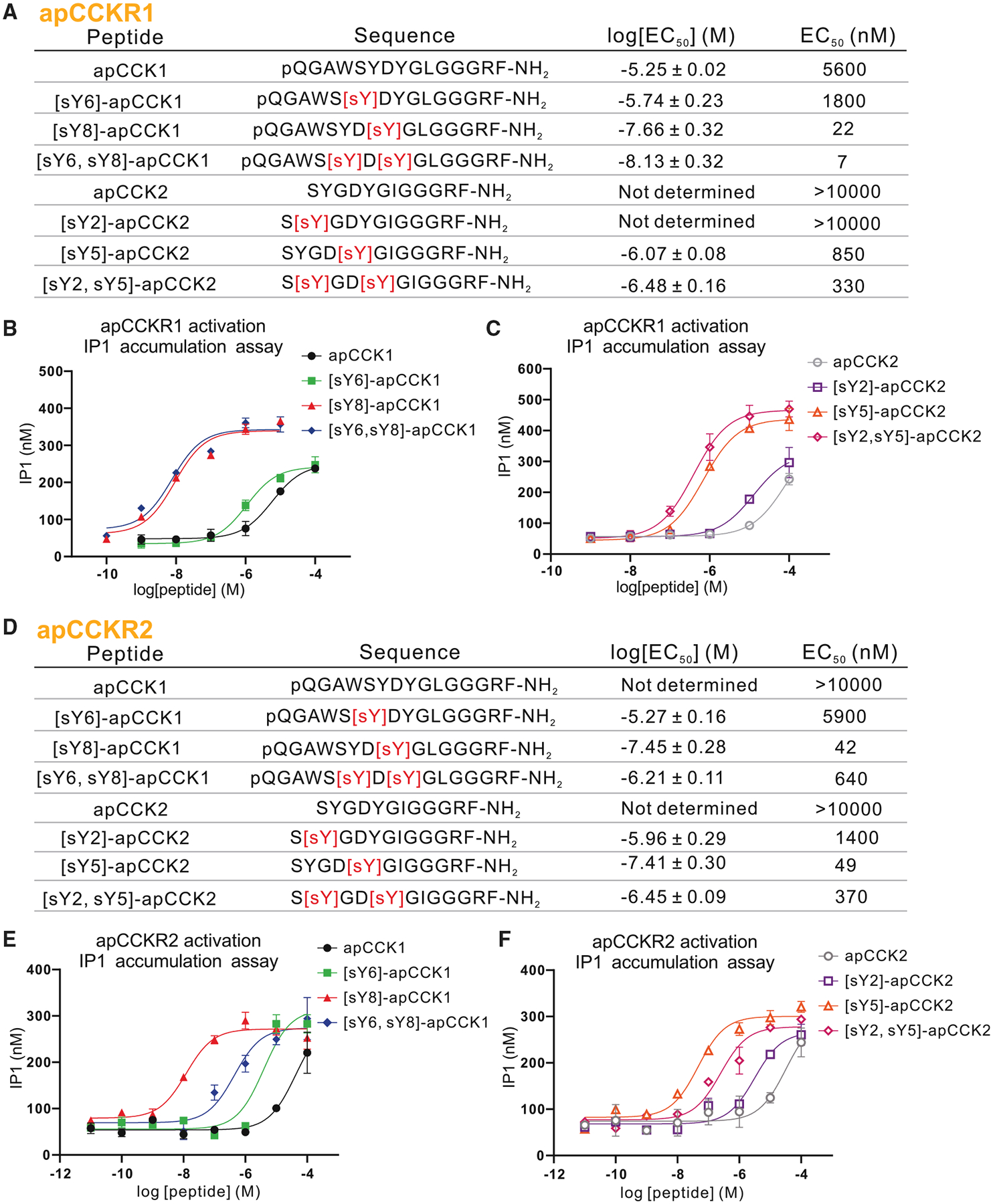
Activation of apCCKs on putative receptors determined by IP1 accumulation assay (A–C) Sequences of all tested apCCK peptides and a summary of the average log[EC_50_] and EC_50_ values for apCCKR1 (A) and representative examples of dose-response curves (B and C). (D–F) Sequences of all tested apCCK peptides and a summary of the average log[EC_50_] and EC_50_ values for apCCKR2 (D) and representative examples of dose-response curves (E and F). Log[EC_50_] values are reported as the mean ± SD from at least three independent experiments. See also Figures S17 and S18.

We tested receptor activation using the IP1 (inositol monophosphate) accumulation assay that measures IP1 generated in the G_αq_ pathway (see STAR Methods). Initial screening with eight forms of apCCKs, at two concentrations (10^−9^ M and 10^−5^ M) showed that apCCKR1 and apCCKR2 responded to most apCCK forms (Figures S17A and S17B), whereas Class-A_GPCR1 (Figure S17C) and Srw_GPCR1 (Figure S17D) did not, confirming that the latter are not *Aplysia* CCK receptors. Subsequent dose-response curves showed that for apCCKR1 (Figures 3A–3C), the approximate order of potency was [sY6, sY8]-apCCK1, [sY8]-apCCK1, [sY2, sY5]-apCCK2, [sY5]-apCCK2, [sY6]-apCCK1, apCCK1, [sY2]-apCCK2, and apCCK2 (Figure 3A). For apCCKR2 (Figures 3D–3F), the approximate potency order was [sY8]-apCCK1, [sY5]-apCCK2, [sY2, sY5]-apCCK2, [sY6, sY8]-apCCK1, [sY2]-apCCK2, [sY6]-apCCK1, apCCK2, and apCCK1 (Figure 3D). These findings suggest that sulfated tyrosine modifications located toward the C-terminal region may be more critical for the physiological functions of apCCKs than those located toward the N-terminal region. Interestingly, for apCCKR1, disulfated forms of apCCK1 and apCCK2 tended to be the most potent (Figures S18A and S18B). In contrast, for apCCKR2, the disulfated forms of apCCK1 and apCCK2 were less potent than the apCCKs with monosulfated tyrosine located toward the C terminus (Figures S18C and S18D). This pattern of activation potency is consistent with the *in vivo* behavioral results, which showed that apCCK1, [sY6]-apCCK1, apCCK2, and [sY2]-apCCK2 did not influence feeding (Figure S9).

Expression profiling (GEO: GSE79231) revealed that *apCCKR1* is widely distributed in the CNS and peripheral tissues (Figure S19A), while *apCCKR2* may be primarily in the eye and statocyst (Figure S19B). *In situ* hybridization showed that *apCCKR1* is present in all central ganglia (Figure S20), mirroring the distribution of apCCK neuropeptides (Figure 1), whereas *apCCKR2* in the CNS is minimal (Figure S21). Taken together, these findings suggest that apCCKR1 and apCCKR2 mediate the behavioral effects of apCCKs on feeding in *Aplysia*.

### Peripheral sources of *Aplysia* CCKs

**Figure 4.**
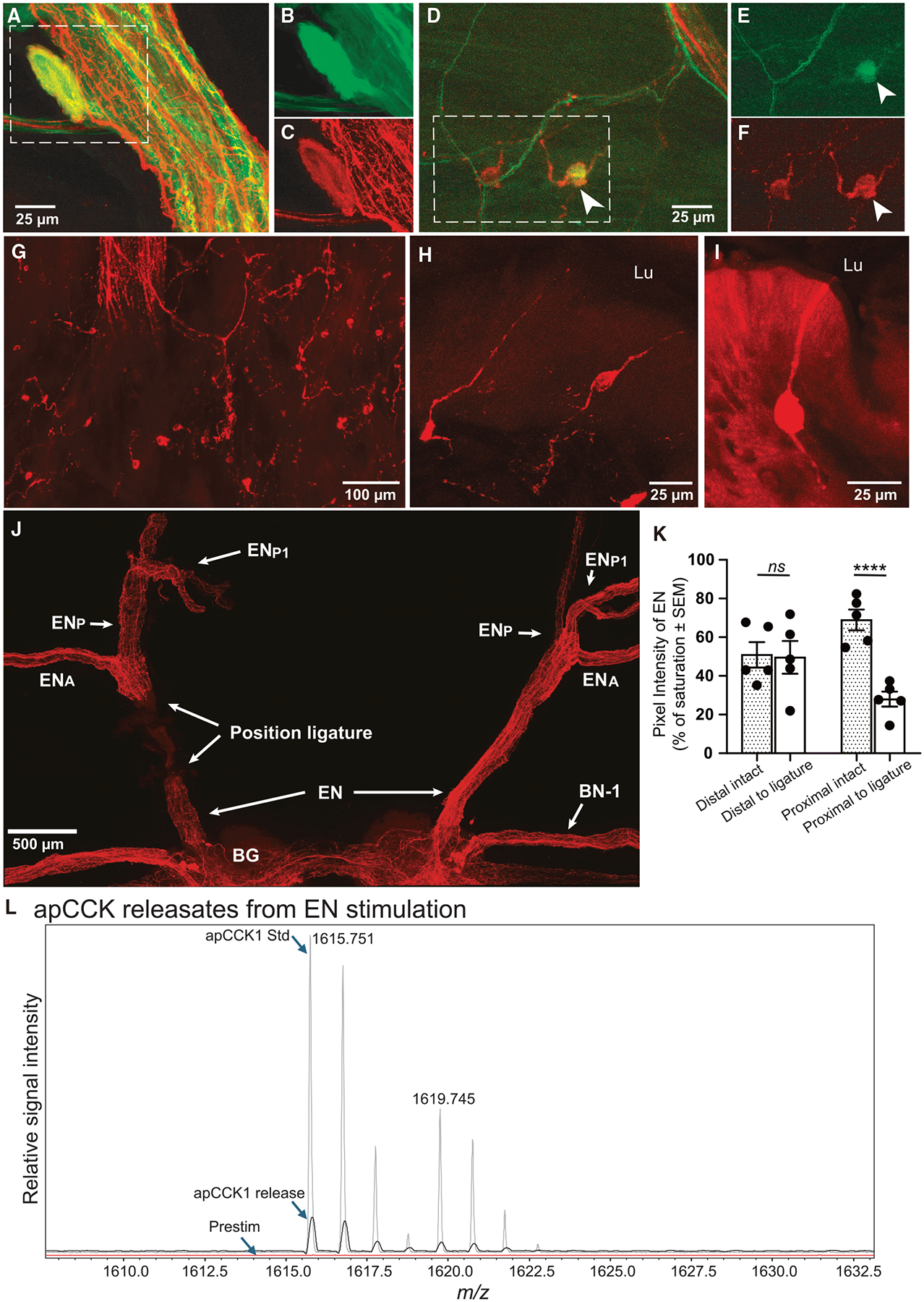
Central projection and distribution of peripheral apCCK neurons, EN ligation, and apCCK releasates induced by EN stimulation (A–C) Area outlined by dashed line in (A) is shown in (B) and (C). A larger EN-backfilled (B) and apCCK-IR neuron (C) is located in the anterior esophagus. (A–C) have the same magnification. (D–F) Area outlined by dashed line in (D) is shown in (E) and (F). Some small apCCK-IR neurons (red, F) were backfilled (green, E) from the EN, indicating that they projected centrally to the buccal ganglion. (D–F) have the same magnification. (G) Distributions of neurons and fibers of apCCK-IR neurons that were scattered in the pharynx, in this case near the opening of the salivary duct. (H and I) Photographs through folds of the mucosal walls. (J) Representative example following EN ligation (left side). BG, buccal ganglion; EN, esophageal nerve; EN_A_, anterior branch of EN; EN_P_, posterior branch of EN; EN_P1_, first branches of EN_P_; BN-1, buccal nerve 1. (K) Plots of EN staining (means ± SEM, *n* = 5) compare the mean pixel intensity (expressed as percent saturation) of regions of interest on the experimental (ligature) versus control (intact) sides. There was a significant effect of ligation only proximal to the ligation, but no effect on the distal side (two-way ANOVA: interaction location × treatment *F*(1, 8) = 55.13, *p* < 0.0001). Bonferroni post hoc test: ****p < 0.0001; ns, not significant. See also Figure S22. (L) Overlaid mass spectra (from MALDI-TOF MS) of apCCK1 standard (Std, gray trace), prestimulation (Prestim, red trace) and EN-stimulated releasates (apCCK1 release, black trace). There is a peak matching the mass of apCCK1 (pQ-apCCK1) in releasates from EN stimulation. The figure also shows distribution of natural isotopes among apCCK1 molecules. The shape of isotopic cluster is defined by the chemical formula of the peptide. The similar isotopic envelopes between apCCK1 standard and releasates serve as further proof of assignment by mass match.

Functioning as a brain-gut peptide in vertebrates, CCK regulates satiety by transmitting peripheral signals from the gastrointestinal tract to the CNS.^20,32^ However, its role in protostomes remains unclear. To investigate peripheral apCCK sources in *Aplysia*, we backfilled the distal branches of the esophageal nerve (EN), which showed strong apCCK-IR (Figures 1E and 1F). Some backfilled neurons were relatively large (i.e., 25–100 μm), although most of these neurons were near large nerve bundles in the submucosa and were not apCCK-IR. One large neuron was along the enteric nerve plexus described previously^72^ and was apCCK immunopositive (Figures 4A–4C).

Other neurons backfilled were small (i.e., 10–20 μm). In four out of seven preparations, these neurons were apCCK-IR (Figures 4D–4F), whereas the absence of double labeling in the other three preparations may be due to the fact that small cells are difficult to backfill. The majority of the double-labeled neurons were part of a large population of apCCK-IR cells with cell bodies in the mucosal layer or in the endothelium of the anterior esophagus and pharynx (Figure 4G). In general, apCCK-IR cells were most numerous in a longitudinal strip just ventral to, but not in the dorsal longitudinal folds (DLFs) described by Howells^73^ (Figure S22). A thinner strip with a moderately dense population of apCCK-IR cells was observed just dorsal to the DLFs. The rostro-caudal location of densely and moderately densely packed apCCK-IR cells largely corresponded to the extent of the DLFs, with few apCCK-IR cells at the level of the orifice of the salivary duct or in the anterior esophagus caudal to the DLFs. Most of these cells have long processes that can collect into nerve branches, classifying them as neurons. Although in most cases the preparations were flat, in some cases there were folds of the pharynx walls that allowed a cross-sectional view of the endothelium, revealing that many apCCK-IR neurons were bipolar with a process that appeared to extend to the lumen (Figures 4H, 4I, and S22C–S22F).

EN ligation in intact animals^13^ reduced apCCK-IR on the proximal side of the ligature, likely due to the degeneration of severed axons whose cell bodies are located peripherally. However, there was no change in apCCK-IR intensity on the distal side (Figures 4J and 4K), supporting a peripheral origin of the majority of apCCK-IR axons in the EN. LC-MS/MS showed the presence of apCCK1 and apCCK2 in EN extracts (Data S4). Specific apCCKs detected depend on the relative abundance of different forms of apCCKs and MS equipment sensitivity.

Thus, the anatomical studies suggest that most of the peripheral apCCK-IR cells are neurons located in the mucosa, some of which project to the buccal ganglia where the feeding-pattern generator is located.

### Release of apCCK in buccal ganglia during EN stimulation

Consistent with a role for the EN in satiation, electrical stimulation of the EN can activate egestive motor programs.^74–76^ To determine the potential role of apCCKs in the generation of egestive programs elicited by EN stimulation, we examined the complement of neuropeptides secreted after EN stimulation using MALDI-TOF MS. For collection of release, a solid-phase extraction (SPE) pipette was placed on the rostral side of the buccal hemi-ganglion near the neuropile and replaced between prestimulation and stimulation collection periods. The neuropilar region is where immunocytochemistry showed prominent staining. MALDI-TOF MS (Figure 4L) detected non-sulfated apCCK1 after EN stimulation, whereas the prestimulation samples showed no presence of apCCK neuropeptides. Along with the LC-MS/MS results (Data S4), these data provide compelling evidence that apCCK is not only present in the EN but also is released at the site of the buccal pattern-generating neurons after EN stimulation.

### apCCKs modulate feeding programs in isolated ganglia

To explore the neural basis of the behavioral effects of apCCKs, we examined their modulation of feeding-motor programs in isolated ganglia. All eight forms of apCCKs were tested on programs induced by CBI-2, a command-like interneuron that is active during food-elicited feeding behavior^56^ and provides potent excitatory inputs to buccal central pattern generator (CPG) interneurons,^54,77^ orchestrating ingestive motor programs (Figure S23A). Stable single-cycle motor programs were evoked by stimulating CBI-2 at 8–10 Hz throughout the protraction phase with a 90-s intertrial interval. Two concentrations, 10^−6^ M and 10^−5^ M, of apCCKs were tested.

**Figure 5.**
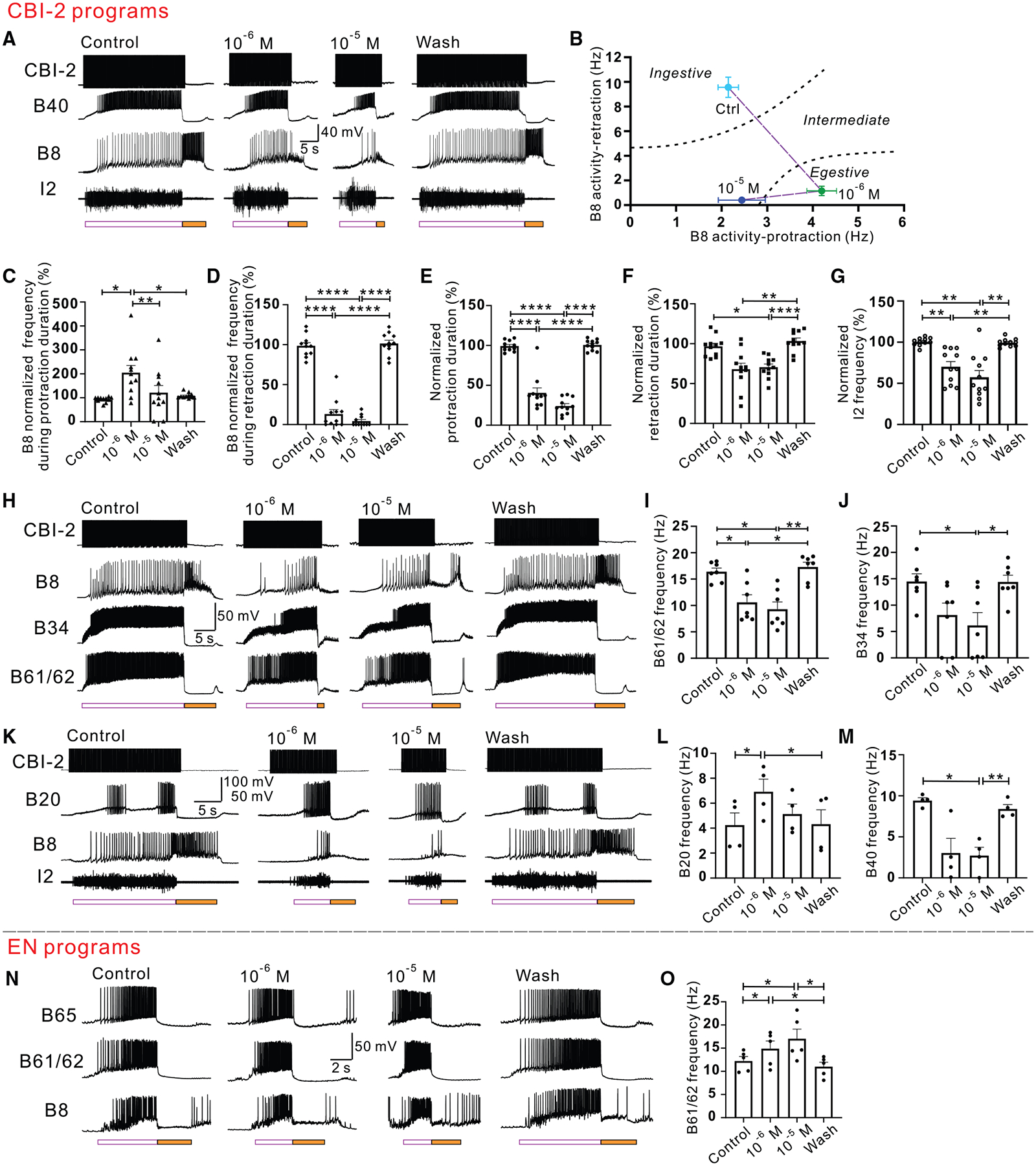
Effects of [sY8]-apCCK1 on CBI-2- and EN-elicited feeding motor programs CBI-2 programs were elicited by stimulation of CBI-2 at 10 Hz with short current pulses. EN programs were elicited by stimulation of EN at 2 Hz. Protraction (open bar) is monitored by activity in I2 nerve, whereas retraction (filled bar) is monitored by sustained hyperpolarization of the protraction interneuron B40 (A), B34 (H), B20 (K) or B65 (N). (A–B) Effects on program type. (A) Representative examples. (B) Group data. Plot of average B8 activity during protraction versus retraction of CBI-2-elicited programs (*n* = 11). (C–F) Group data for the effects of [sY8]-apCCK1 on B8 frequency during protraction (C, *F*(3, 30) = 7.838, *p* = 0.011) and retraction (D, *F*(3, 30) = 145.5, *p* < 0.0001), and protraction duration (E, *F*(3, 30) = 110.2, *p* < 0.0001) and retraction duration (F, *F*(3, 30) = 11.46, *p* = 0.0005). (G) Group data showing [sY8]-apCCK1 significantly decreased the firing frequency of the I2 nerve (*F*(3, 30) = 20.93, *p* < 0.0001). (H–J) [sY8]-apCCK1 decreased B61/62 and B34 frequency in CBI-2 programs. (H) Representative examples. (I and J) Group data (I, *F*(3, 18) = 21.29, *p* = 0.0018; J, *F*(3, 18) = 13.35, *p* = 0.0019). (K and L) [sY8]-apCCK1 increased B20 frequency in CBI-2 programs at low concentration (10^−6^ M) but not at higher concentration (10^−5^ M). (K) Representative examples. (L) Group data (*F*(3, 9) = 19.28, *p* = 0.0043). (M) [sY8]-apCCK1 decreased B40 frequency in CBI-2 programs (*F*(3, 9) = 18.69, *p* = 0.0075). (N and O) [sY8]-apCCK1 increased B61/62 frequency in EN programs. (N) Representative examples. (O) Group data (*F*(3, 15) = 18.52, *p* = 0.0013). One-way ANOVAs are used in (C)–(G), (I), (J), (L), (M), and (O) with their Tukey post hoc tests: *p < 0.05, **p < 0.01, ****p < 0.0001. Error bars denote SEM. See also Figures S23, S24, and S25.

Each peptide manifested diverse effects on feeding-motor programs. [sY8]-apCCK1 (Figures 5A–5G) shifted ingestive programs to egestive (Figure S23B) at 10^−6^ M and reduced B8 neuron activity at 10^−5^ M (Figures 5A and 5B). Group data showed that B8 activity during protraction at 10^−6^ M was significantly increased, but decreased at 10^−5^ M (Figure 5C). In contrast, B8 activity during retraction significantly decreased at both 10^−6^ M and 10^−5^ M (Figure 5D). Furthermore, [sY8]-apCCK1 shortened the protraction and retraction duration of CBI-2 programs (Figures 5E and 5F).

Similarly, [sY6, sY8]-apCCK1 (Figures S24A–S24F), [sY5]-apCCK2 (Figures S24G–S24L), and [sY2, sY5]-apCCK2 (Figures S24M–S24R) shortened protraction duration and reduced B8 frequency during retraction to different extents. Additionally, all apCCKs with disulfated and monosulfated tyrosine located toward the C terminus reduced the firing frequency of the I2 nerve (Figures 5G, S24F, S24L, and S24R), which contains axons of protraction motoneurons, e.g., B61/62 neurons,^78^ reflecting protraction activity. This inhibitory effect was also observed directly in both B61/62 and its presynaptic interneuron B34 (Figures 5H–5J).^52,79^ Weaker B61/62 activity is associated with weaker programs.^78^ In contrast, apCCKs with non-sulfated and monosulfated tyrosine located toward the N terminus had no significant effect (Figure S25).

Generally, motor neuron activity in the *Aplysia* feeding circuit is controlled by CPG interneurons. Therefore, we assessed the impact of apCCKs on two critical CPG neurons, B40 and B20, during the motor programs, which orchestrate distinct feeding patterns.^13,53,54,79^ Specifically, both B40 and B20 are active during protraction, with B40 facilitating ingestive patterns by enhancing B8 activity during retraction and B20 promoting egestive patterns by enhancing B8 activity during protraction. The B20 firing frequency increased with 10^−6^ M [sY8]-apCCK1 (Figures 5K and 5L), whereas it decreased with 10^−5^ M [sY8]-apCCK1, which paralleled B8 activity changes during protraction (Figure 5C). In contrast, B40 firing frequency decreased with [sY8]-apCCK1 (Figures 5A and 5M), which paralleled B8 activity change during retraction (Figure 5D). These data indicate that when [sY8]-apCCK1 converts CBI-2-elicited ingestive programs to egestive programs at lower concentrations, the activity of the interneurons that participate in the generation of egestive and ingestive programs undergoes concomitant changes.

In conclusion, the apCCKs with disulfated and monosulfated tyrosine located toward the C terminus had distinct effects on feeding programs. This suggests that different forms of apCCKs may act in concert to reduce food intake and promote satiety.

### apCCK-mediated synaptic and intrinsic plasticity

**Figure 6.**
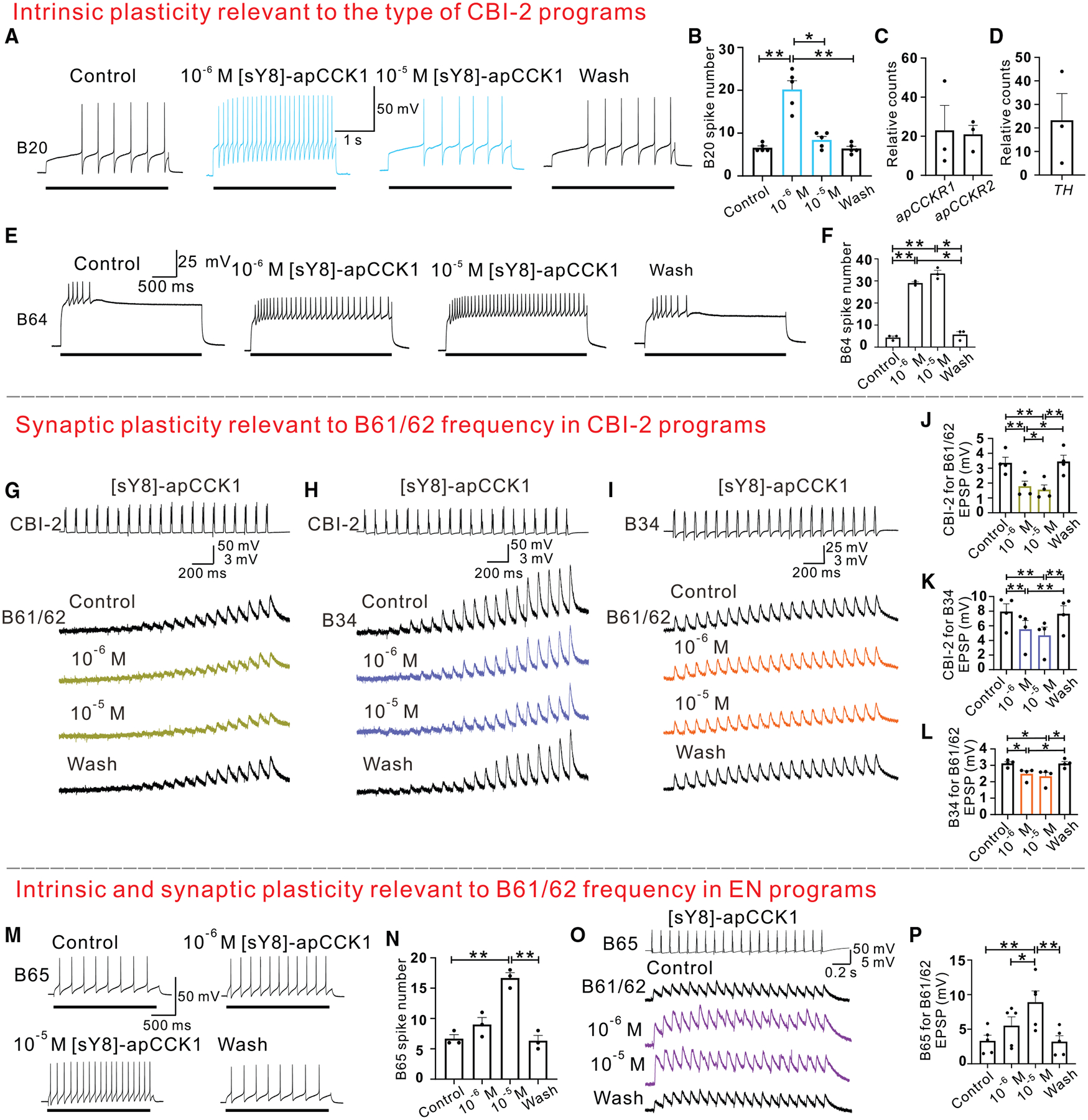
[sY8]-apCCK1 induced intrinsic and synaptic plasticity underlie modulation of feeding program types and protraction activity (A and B) The effect of [sY8]-apCCK1 on B20 excitability. (A) Representative examples. (B) Group data (*F*(3, 12) = 36.96, *p* = 0.0015). (C and D) SMART-seq showing expression of *apCCKR1*, *apCCKR2* (C), and *TH* gene (D) in B20 neurons. Relative counts are obtained by removing batch effects. (E and F) The effect of [sY8]-apCCK1 on B64 excitability. (E) Representative examples. (F) Group data (*F*(3, 6) = 191, *p* = 0.0001). (G–L) [sY8]-apCCK1 reduced the synaptic strength between CBI-2 and B61/62 neurons (G, representative examples; J, group data, *F*(3, 9) = 80.47, *p* = 0.0024), between CBI-2 and B34 neurons (H, representative examples; K, group data, *F*(3, 9) = 83.19, *p* = 0.0009), and between B34 and B61/62 neurons (I, representative example; L, group data, *F*(3, 9) = 29.59, *p* = 0.0074). (M and N) [sY8]-apCCK1 enhanced B65 excitability. (M) Representative examples. (N) Group data (*F*(3, 6) = 86.34, *p* = 0.0089). (O and P) [sY8]-apCCK1 increased the synaptic strength between B65 and B61/62 neurons. Note that because the first EPSP in each stimulation trial tended to be the largest, the amplitudes of the EPSPs were taken from the first EPSP. (O) Representative examples. (P) Group data (*F*(3, 12) = 29.54, *p* = 0.0003). One-way ANOVAs are used in (B), (F), (J)–(L), (N), and (P) with their Tukey post hoc tests: *p < 0.05, **p < 0.01. Error bars denote SEM. All quantified EPSPs were taken from the last EPSP during the 2-s stimulation, except for the EPSPs from B65 to B61. See also Figures S26, S27, S28, and S29.

To characterize apCCK mechanisms of action, we investigated their effects on synaptic and intrinsic plasticity of key circuit elements. [sY8]-apCCK1 significantly enhanced B20 excitability at 10^−6^ M but had no significant effect at a higher concentration (Figures 6A and 6B), aligning with the observed changes in B20 activity during feeding programs (Figure 5L). Surprisingly, it also enhanced B40 excitability (Figures S26A and S26B), contrasting with its activity in programs (Figure 5M). However, [sY8]-apCCK1 decreased synaptic strength between CBI-2 and B40 (Figures S26C and S26D). These data suggest that [sY8]-apCCK1 modulates feeding programs by altering the intrinsic plasticity of B20 and by altering the synaptic plasticity from CBI-2 to B40.

Given that [sY8]-apCCK1 altered B20 excitability (Figures 6A and 6B), we conducted SMART-seq of three B20 neurons. Both *apCCKR1* and *apCCKR2* were detected (Figure 6C). Moreover, the observed behavioral and motor program effects of the eight forms of apCCKs correlate with activation potency of these peptides on apCCKR1 and apCCKR2 (Table S1), suggesting that both receptors could mediate apCCK actions on the CNS, including B20 excitability. Because B20 is a dopaminergic neuron^80^ and neighboring cells are not, we determined whether the B20 neurons express the tyrosine hydroxylase (*TH*) gene. To date, the *TH* gene has not been reported in *Aplysia*. BLAST searches in the NCBI and AplysiaTools databases with *Drosophila TH* gene returned one sequence from AplysiaTools that is very similar to other *TH* genes (Figure S27 and Data S8). The phylogenetic tree also suggests that this putative sequence is likely to be *Aplysia TH* (Figure S28). Importantly, this *TH* gene was indeed expressed in the neurons that we collected (Figure 6D). This confirms the identity of these neurons as B20s.

We then identified neural targets associated with the apCCK-mediated reduction in protraction duration. [sY8]-apCCK1 enhanced the excitability of the retraction interneuron B64 (Figures 6E and 6F),^51^ which is pivotal in terminating protraction. Thus, B64 likely mediates apCCK effects on protraction duration. These findings indicate that apCCKs influence program types primarily by modulating the intrinsic plasticity of relevant interneurons.

Another significant effect of apCCKs is the reduction in protraction motoneurons (B61/62) and protraction interneuron (B34) firing frequency during CBI-2-elicited programs (Figures 5H–5J). Unexpectedly, [sY8]-apCCK1 enhanced the excitability of both B61/62 and B34 (Figures S26E–S26H), which could not explain the observed reduction in B61/62 frequency during motor programs. SMART-seq data indicate that both receptors are expressed in B34 (Figure S26I), which could mediate the changes in B34 excitability by apCCKs. Subsequent examination revealed that [sY8]-apCCK1 decreased synaptic strength between CBI-2 and B61/62 (Figures 6G and 6J), between CBI-2 and B34 (Figures 6H and 6K), and between B34 and B61/62 (Figures 6I and 6L). Similar effects were observed with [sY6, sY8]-apCCK1 (Figure S29). These data suggest that apCCKs modulate protraction activity primarily by influencing synaptic plasticity through direct and indirect pathways.

Although protraction (B61/62) activity is inhibited by apCCKs during CBI-2-elicited programs, this might not be the case for the egestive motor programs elicited by EN stimulation, which could contribute positively to satiety. We therefore examined [sY8]-apCCK1 effects on B61/62 activity in EN-elicited egestive motor programs. Notably, the B61/62 firing frequency increased rather than decreased in EN-elicited programs (Figures 5N and 5O). This difference may be explained in part by effects on the interneuron B65, which is strongly active during EN programs but not during CBI-2 programs,^56,81–83^ whereas the opposite is true for B34.^54,61^ B65 elicits fast excitatory postsynaptic potentials (EPSPs) in B61/62.^81^ [sY8]-apCCK1 increased B65 excitability (Figures 6M and 6N) and EPSPs from B65 to B61/62 (Figures 6O and 6P), which could account for the increase of B61/62 activity during EN programs.

In summary, the apCCKs influence feeding behavior and promote satiety by modifying motor programs and circuit dynamics. In particular, they exert complex effects on synaptic transmission and the intrinsic properties of key interneurons.

## DISCUSSION

In this study, we leveraged the tractable model system of the mollusk *Aplysia* to explore the roles of apCCKs in establishing a satiety motivational state. By integrating chemical, cellular, molecular, behavioral, anatomical, and neurophysiological approaches, we demonstrated that the *Aplysia* CCKs act as brain-gut peptides, with gut apCCK neurons projecting directly to the feeding circuit. The apCCKs establish satiety by inducing multiple changes in feeding-motor programs that involve both alterations in synaptic transmission and alterations in the intrinsic properties of key circuit elements.

### *Aplysia* CCKs as brain-gut peptides and classical satiety signals

CCK was first identified in mammals as a satiety peptide that inhibits food intake.^23,29,30^ Extensive evidence supports its role as a brain-gut peptide^31,84–86^ with gut CCK cells (neuropods) activated by food intake. These cells utilize glutamate to stimulate vagus afferents.^32^ Additionally, exogenous CCK can activate NTS neurons containing leptin receptor or peptides (neuronatin or pro-opiomelanocortin).^87–89^ Finally, CCKergic neurons in the NTS may influence feeding through the parabrachial nucleus calcitonin gene-related peptidergic neurons^90^ or the central nucleus of amygdala.^91^ While both gut and brain CCK contribute to feeding suppression,^92,93^ these effects likely involve multiple circuit and signaling pathways.

In non-mammalian vertebrates^26,94–96^ and deuterostome invertebrates,^71^ CCK appears to inhibit feeding and is present in both the gut and brain, suggesting conservation of function. In protostomes, most work has been carried out in arthropods, where SKs also inhibit feeding^40–43^ but are only found in the CNS, with one possible exception.^97^ This restricted distribution suggests that SKs may not function as classical satiety peptides in arthropods.

In other protostome species, particularly within the superphylum Lophotrochozoa (i.e., annelids, mollusks, and brachiopods), few data are available. Early research in mollusks—prior to the genomic era—used mammalian CCK or gastrin antibodies^98–100^ and exogenous CCK-8^99,101–103^ to identify putative CCK-positive neurons and infer functional roles. However, these findings may not reflect authentic CCK signaling. For instance, a prior study suggested that the large B13 motoneurons in the *Aplysia* buccal ganglion are apCCK immunopositive,^99,104^ whereas our data reveal only a cluster of small neurons near the EN/BN-1 (Figures 1A and 1E), suggesting that B13 is not apCCK immunopositive. Recent studies addressing bona fide molluscan CCK signaling include a report identifying bioactive CCKs in the hindgut of *Crassostrea gigas*^70^ without determining their role in satiety. Another mapping study in *Biomphalaria glabrata* found no CCK-type peptide expression in the digestive system.^105^ In contrast, we demonstrate that apCCKs act as brain-gut peptides to regulate satiety.

The apCCK precursor could potentially give rise to eight peptides. MS revealed that seven of the eight mature forms are present in the *Aplysia* CNS. The “missing” peptide is the disulfated apCCK2 ([sY2, sY5]-apCCK2). Detecting disulfated peptides via MS is challenging, in part due to their low abundance.^106^ Additionally, the sulfate group is not stable and generates a relatively weak signal due to its reduced ionization efficiency (a problem even more pronounced for disulfated isoforms). To overcome these challenges, we employed trapped-ion mobility spectrometry (TIMS), which allowed for confident identification of sulfated apCCKs via their molecular shape correlated to retention times and fragmentation patterns. TIMS also allowed distinction of isobaric peptide forms such as sY6 and sY8 of apCCK1 or sY2 and sY5 of apCCK2. Additionally, sulfated peptides have a similar nominal mass as phosphorylated peptides, which could be resolved by separation via ion mobility.

Anatomical studies (Figures 1 and 4) revealed apCCK localization in both the CNS and periphery, with gut-derived apCCK neurons projecting directly via the EN to the buccal ganglion, which houses the feeding central pattern generator. Prior studies found that severing the EN delays satiation in *Aplysia*.^107^ Thus, previous studies have indicated that EN afferents are important for satiation. Here, we show that EN stimulation triggers apCCK release in the buccal ganglion (Figure 4L). Behaviorally, four of the eight apCCKs inhibit food intake (Figures 2A–2D), with [sY8]-apCCK1 showing the strongest effect (Figure 5). In the isolated CNS, apCCKs inhibit protraction activity during the motor program, an *in vitro* analog of food intake suppression. Interestingly, at lower concentration, [sY8]-apCCK1 promoted egestive programs. In parallel, satiation biases behavior toward rejection in mammals.^9–12,14,108^ Importantly, other neuropeptides, such as *Aplysia* NPY^13^ and SCP (small cardioactive peptide),^109^ are also expressed in the EN and promote egestive programs, suggesting that satiety regulation in *Aplysia*, like in mammals,^16,17^ involves multiple peptides. Dopamine is likewise present in the EN,^110^ although its role in satiety remains to be determined.

Taken together, our data indicate that the CCKs in *Aplysia* exhibit functional and distributional features similar to those of their mammalian counterparts, suggesting a conserved role in regulating satiety. In contrast, functions of arthropod CCKs/SKs may have diverged. One notable difference between *Aplysia* and mammals lies in the CCK projection pattern. *Aplysia* gut CCK neurons, at least in part, project directly to the feeding CPG (Figure 4), whereas in mammals the gut-to-CNS CCK signaling appears to be more complex, i.e., it presumably involves additional circuit elements. Moreover, *Aplysia* gut CCK cells have long processes, indicating that they are true neurons, unlike the short processes of mammalian CCK neuropods.^32^ Despite these differences, our findings underscore the value of using *Aplysia* as a model to study CCK function and its conservation across species.

### apCCK receptors and the roles of apCCK post-translational modification in receptor activation

In vertebrates, two CCK receptors are present: CCK1R, which is found in both the CNS and periphery, and CCK2R, which is primarily in the CNS.^111,112^ This dual-receptor system was also observed in the chordate *Ciona intestinalis*,^69^ while a single receptor was reported in a deuterostome invertebrate, the starfish *Asterias rubens* (phylum Echinodermata).^71^ Among protostomes, some arthropods and nematodes^43^ have two receptors, e.g., in *Drosophila*^113,114^ and *Caenorhabditis elegans*,^27^ whereas others have one (e.g., cockroach *Periplaneta americana*)^40^ or none^40,43^ (Data S9), suggesting divergent evolution for CCK signaling in these taxa.

Similar to vertebrates, in mollusks, including *Crassostrea gigas*^70^ and *Aplysia*, two CCK receptors are present. Our analysis of apCCKRs revealed high activation potency to the four bioactive apCCKs (Table S1). *In situ* hybridization (Figure S20) and gene-expression data (Figure S19A) suggest that apCCKR1 is localized in both the CNS and periphery, whereas apCCKR2 may be located more peripherally (Figures S19B and S21). Future work is needed to clarify the central and peripheral distributions of apCCKR2.

The identification of receptors has enhanced our understanding of how post-translational modification (PTM) of apCCKs affects receptor activation. Most species, including vertebrates, possess CCK with a single Tyr residue that can undergo sulfation, generating multiple CCK isoforms with different lengths.^27,43,71,115^ In mammals, sulfated CCK exhibits greater potency on CCK1R, whereas the non-sulfated and sulfated forms show comparable potency on CCK2R.^33,43,116^

CCKs containing two Tyrs have been identified in six species, chordata *Ciona intestinalis*,^117^ three crustaceans (*Penaeus monodon*,^48^ *Litopenaeus vannamei*,^106^ and *Homarus americanus*^118^), and two mollusks (*Crassostrea gigas*^70^ and *Aplysia*) (Data S9). Uniquely, both apCCK1 and apCCK2 contain two Tyrs, potentially generating eight forms of apCCKs following sulfation (Figure S4). Studies in *Ciona intestinalis*^69^ and *Crassostrea gigas*^70^ demonstrate that sulfated CCKs show higher receptor activation potency than non-sulfated forms, and the monosulfated CCKs with the more C-terminal Tyr tend to have higher potency than CCKs with the more N-terminal Tyr, which holds for *Aplysia*. Additionally, in *Aplysia*, disulfated apCCK peptides exhibited differential potency at the two receptor subtypes, demonstrating increased potency on apCCKR1 and decreased potency on apCCKR2 (Figure S18). The effects of various apCCK forms on behavior, motor programs, and cellular and synaptic plasticity match with their potency on both receptors (Table S1), suggesting that both apCCKR1 and apCCKR2 mediate apCCK’s satiety effect, with PTMs playing a crucial role in modulating receptor activation and downstream signaling.

### Synaptic and intrinsic plasticity mediated by apCCKs during the development of satiety motivational state

Synaptic and intrinsic plasticity are critical for learning^119–122^ and peptidergic modulation.^123–125^ While early research emphasized synaptic plasticity in learning, subsequent studies highlight the significance of intrinsic plasticity.^126–130^ In peptidergic modulation, research across various model systems consistently demonstrates that peptides influence the excitability of specific circuit elements to produce adaptive changes in motor output.^3,34–37,131^ Peptides also influence synaptic properties.^35,124,125,132–134^ However, to our knowledge, few studies have elucidated how these peptidergic actions coordinate multiple behavioral changes. In principle, either synaptic or intrinsic plasticity could mediate these effects, or both may be required to produce complex behavioral modifications.

**Figure 7.**
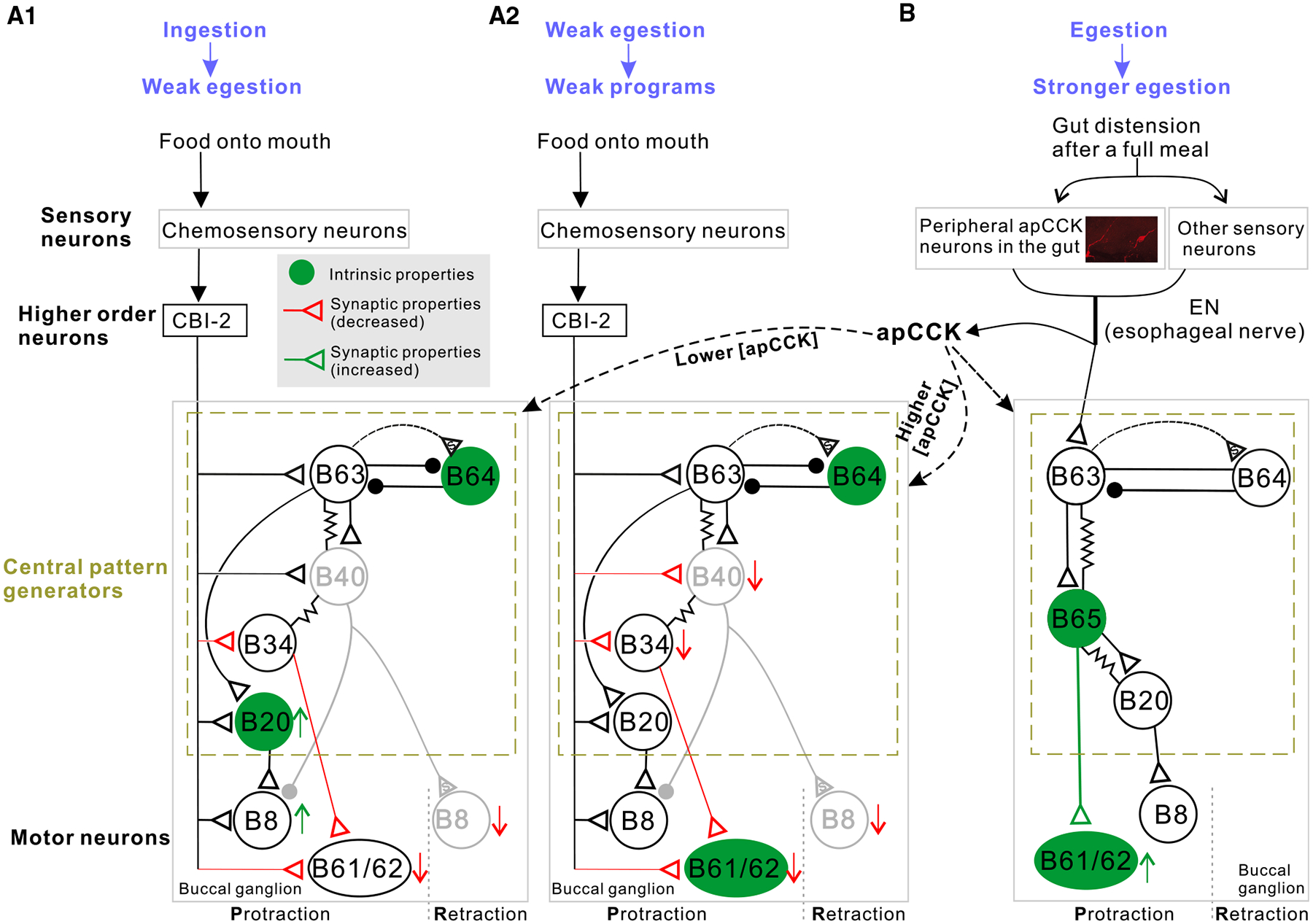
Schematic diagrams illustrating apCCK-mediated synaptic and intrinsic plasticity to induce satiety Food presentation to the mouth activates the cerebral higher-order neuron CBI-2, which in turn induces ingestive programs by providing larger excitation to B40 than to B20. Peripheral apCCK cells in the gut (fluorescent image from Figure 4H) are likely activated by gut distension after food consumption. These peripheral apCCK cells project directly through the EN (the esophageal nerve) to the buccal ganglion to release apCCKs to act on the pattern-generator elements there. Other sensory neurons might also contribute. (A) CBI-2-elicited programs. (A1) At lower apCCK concentration, apCCK-mediated intrinsic plasticity in protraction interneuron B20 (increased excitability at relatively low concentration, green) is responsible for changes in type of motor programs from ingestion to egestion, whereas that in retraction interneuron B64 (increased excitability, green) is responsible for shortened protraction duration. apCCK-mediated synaptic plasticity (reduced EPSPs, red) from CBI-2 and interneuron B34 to protraction motoneuron B61/62 and from CBI-2 to B34 is responsible for reduction in B61/62 frequency, i.e., weak egestive programs. (A2) At higher apCCK concentration, B20 excitability returned to the control level, and there is also a reduction of excitation from CBI-2 to B40 (red). Overall, activity of both B8 and B61/62 would be lower, resulting in weak programs. (B) For EN-elicited egestive programs, apCCK promotes these programs by increasing B61/62 activity. apCCK-mediated synaptic plasticity from B65 to B61/62 (increased EPSPs, green) and intrinsic plasticity in both B65 and B61 (increased excitability, green) are responsible for increased B61/62 frequency. Note that for B61/62 activity, during CBI-2 programs, apCCK-mediated synaptic and intrinsic plasticity is a push-pull mechanism (A), but during EN-elicited egestive programs, the two forms of plasticity work jointly to enhance egestive programs (B).

Our study provides the first evidence that apCCK-mediated synaptic and intrinsic plasticity work in concert to coordinate multiple changes in motor output during motivational-state shifts (Figure 7). To bias motor programs toward egestion, apCCK primarily enhances the excitability of the egestion-promoting B20 interneuron,^53^ an effect that could be mediated by binding to apCCKR1 and apCCKR2 because both are present in B20 as demonstrated by single-cell SMART-seq (Figure 6C). Consistent with apCCK effects on motor programs, this excitatory effect is concentration dependent, occurring primarily at lower concentrations (Figures 6A and 6B), suggesting complex receptor-mediated regulation. Additionally, apCCK-induced increased excitability of retraction interneuron B64^51,135^ shortens protraction durations, further promoting egestive programs.

A key manifestation of motor-program inhibition is the reduced activity recorded in the I2 nerve, which contains axons of the protraction motoneurons B61/62. In this context, intrinsic and synaptic plasticity play distinct roles in programs elicited by CBI-2, a cerebral higher-order neuron activated by food during feeding (Figure 7A),^56^ compared to programs elicited by EN, which is activated when food enters the gut (Figure 7B).^75,76^ For CBI-2-driven programs, the apCCKs actually increase the excitability of B61/62 and B34, a buccal interneuron that is presynaptic to B61/62. In contrast, the apCCKs reduce the synaptic input B61/62 receive directly from both B34 and CBI-2 and reduce indirect excitation in that synaptic transmission between CBI-2 and B34 is also suppressed. Consequently, synaptic inputs likely take precedence over excitability, a logical mechanism given that modulation of excitability is most effective when synaptic drives remain stable. The opposing effects of apCCK on B61/62 excitability and its excitatory synaptic input may confer an adaptive advantage. Instead of unidirectional inhibition, this contrast creates a push-pull mechanism that enables B61/62 inhibition while preserving its ability to rapidly resume normal activity when a behavioral-state shift is required. Similar opposing functional effects have been observed in the *Aplysia* feeding circuit in priming and task switching.^136^

Moreover, synaptic plasticity enables input-specific modulation of target neurons by differentiating signals from CBI-2 and EN. While the apCCKs reduce direct and indirect excitatory input from CBI-2 to B61/62, they simultaneously enhance synaptic input to B61/62 from B65—an interneuron selectively activated during EN-driven programs.^56,81–83^ The increased B61/62 excitability acts synergistically with the enhanced B65-B61/62 synaptic transmission during EN-induced programs. This selective modulation is physiologically significant because it allows apCCKs to suppress CBI-2-driven ingestive programs while facilitating robust EN-driven egestive programs in satiety states.

In summary, we have demonstrated that apCCKs function as satiety peptides and have elucidated their underlying neural mechanisms. We provide the first evidence of gut apCCK cells directly projecting to and modulating the feeding circuit in a protostome. Importantly, we showed that apCCKs affect both synaptic transmission and the intrinsic properties of specific circuit elements to induce multiple changes leading to motivational-state shifts. This constitutes an elegant mechanism for modulating a complex neural circuit. This dual-plasticity approach enables nuanced regulation of the intricate *Aplysia* feeding circuit, illustrating the sophisticated neural modulation in response to motivational states. Given the conservation of CCK-type signaling between *Aplysia* and mammals, our findings have significant implications for mammalian CCK research and advance our understanding of satiety signaling mechanisms.

### Limitations of the study

Taking advantage of the well-defined *Aplysia* feeding behavior and circuit, our results support a role of apCCKs in satiety. However, the current lack of genetic tools for neuron-specific labeling and manipulation in *Aplysia* limits our ability to assess whether peripheral apCCK neurons are activated by food intake and whether apCCKs are required for the feeding reduction that follows satiation. Addressing these questions will require future studies. Additionally, identifying the intracellular signaling pathways downstream of apCCK receptor activation and comparing them to other neuromodulatory systems would be an important direction for future research.

## RESOURCE AVAILABILITY

### Lead contact

Requests for further information, resources, and reagents should be directed to and will be fulfilled by the lead contact, Jian Jing (jingj01@live.com).

### Materials availability

This study did not generate new unique reagents.

### Data and code availability

- Single-cell SMART-seq data are publicly available at NCBI GenBank, accession number GEO: GSE296741.
- Microscopy data reported in this paper will be shared by the lead contact upon request.
- This paper does not report any original code.
- Any additional information required to reanalyze the data reported in this paper is available from the lead contact upon request.

## STAR★METHODS

### EXPERIMENTAL MODEL DETAILS

#### *Aplysia* experiments

Experiments were performed on *Aplysia californica* (60–350 g) obtained from Marinus Scientific (Long Beach, CA) or RSMAS National Resource for *Aplysia* (Miami, FL). Animals were maintained in an aquarium with a circulating artificial seawater at 14°C–16°C and the room was equipped with a 12:12 h light-dark cycle, with 6 a.m.−6 p.m. as the daylight. Before dissection, animals were anesthetized by injection of isotonic 333 mM MgCl_2_ (about 50% of body weight) into the body cavity.

### METHOD DETAILS

#### Peptide synthesis

Peptides were either synthesized via Fmoc solid-phase peptide synthesis by the lab of James W. Checco at the University of Nebraska-Lincoln, or commercially obtained from GuoPing Pharmaceutical Co., Ltd. or Sangon Biotech (Shanghai) Co., Ltd.

For peptides synthesized in James W. Checco’s lab, a representative protocol follows. Peptides were synthesized on NovaPEG rink amide resin (Novabiochem, 855047). For each coupling reaction, 6 equivalents of Fmoc-protected amino acid, 5.4 equivalents of O-(1H-6-Chlorobenzotriazole-1-yl)-1,1,3,3-tetramethyluronium hexafluorophosphate (HCTU), and 12 equivalents of N-methylmorpholine (NMM) in dimethylformamide (DMF) were used. Coupling was performed for 30 min–overnight at room temperature while stirring. The resin was then rinsed with DMF and deprotection was performed using 20% piperidine in DMF (v/v) at room temperature while stirring for 15 min. For the peptides containing sulfotyrosine ([sY6]-apCCK1, [sY8]-apCCK1, [sY6, sY8]-apCCK1, [sY2]-apCCK2, [sY5]-apCCK2, and [sY2, sY5]-apCCK2), Fmoc-Tyr (SO_3_nP) (Novabiochem, 8.52347) was used for the incorporation of sulfotyrosine. Some stocks of the peptides were synthesized with one or more ^13^C-labeled amino acids to differentiate between synthetic and endogenous peptides by MS. The peptides were cleaved using 95% trifluoroacetic acid (TFA), 2.5% triisopropylsilane (TIS), and 2.5% water for 3–4 h, precipitated using cold diethyl ether, and then pelleted by centrifuging at 3,000 × g for 10 min.

For the peptides without sulfotyrosine (apCCK1, apCCK2), cleaved peptides were purified using reverse-phase HPLC using water with 0.1% TFA (solvent A) and acetonitrile (ACN) with 0.1% TFA (solvent B). For peptides that contain sulfotyrosine, a final deprotection of the neopentyl protecting group on the sulfotyrosine side chain was performed by dissolving the crude peptide in 1.5–3 mL of 2:1 mixture of 2 M ammonium acetate in water and ACN with overnight incubation at 37°C while shaking. After this deprotection, peptide purification was performed by reverse-phase HPLC using 0.1 M ammonium acetate in water (solvent A) and 100% ACN (solvent B). Peptide identity was confirmed using MALDI-MS and the final peptide purity was assessed using reverse-phase HPLC.

#### Bioinformatic analysis of peptide precursors and receptors

To identify *Aplysia* CCK precursor and receptors, we took advantage of bioinformatic methods. Specifically, for apCCK precursor, we performed a BLAST search in the NCBI database using a previously reported putative peptide sequence (QGAWSYDYGLGGGRF),^70^ retrieving two predicted sequences (accession number: XM_005096206.3, XM_005096207.3), and a related sequence named “betsin” (accession number: GU973878). Using the RNA sequence from NCBI (XM_005096206.3), we also found a DNA sequence (DNA: HiC_scaffold_2:24339260–31742029) in AplysiaTools (http://aplysiatools.org/)^67^ that produces a similar but longer mRNA sequence (TRINITY_DN10267_c0_g1_i2) (Figure S1). The two mRNA sequences from NCBI (Figure S1A), though varying in length, likely result from alternative splicing and contain the same open reading frame (ORF) encoding the same protein. Notably, no published studies exist on “bestin”. The mRNA sequences align with the ORF sequences from NCBI database (XM_005096206.3, XM_005096207.3 and GU973878) as well as AplysiaTools (TRINITY_DN10267_c0_g1_i2), confirming the presence of a CCK precursor in *Aplysia*.

For *Aplysia* CCK receptors, we used the keywords “*Aplysia* cholecystokinin receptor” or “*Aplysia* CCK receptor” to search NCBI protein database, and then used the putative sequences to search AplysiaTools databases for comparison and to find more complete sequences. Specifically, a search of NCBI yielded four sequences (XP_012946450.2, XP_012936249.1, XP_005110494.2, and XP_005090536.1), but three out of the four were incomplete GPCRs (XP_012946450.2 (Figure S10, Data S5), XP_012936249.1 (Figure S11, Data S5), and XP_005110494.2 (Figure S12, Data S5)). The only complete sequence (XP_005090536.1) was identical to that in the AplysiaTools database (Figure S13, Data S5). Further search in the AplysiaTools databases resulted in the retrieval of full sequences for the three incomplete receptors (accession numbers: TRINITY_DN21082_c0_g2_i6.p1, TRINITY_DN21082_c0_g1_i3.p1, and asmbl_32627.p1, Data S5). All four exhibited characteristic features of GPCRs such as ERY/ERF near the third transmembrane domain and NPXXY/HPXXY in the seventh transmembrane domain (Figure S14A). To further investigate whether these four putative receptors GPCRs were indeed apCCK receptors, we conducted BLAST searches of each sequence against NCBI in species with well-characterized protein sequences (Data S6), including *Caenorhabditis elegans*, *Drosophila melanogaster*, *Danio rerio* and *Mus musculus*. Two sequences (TRINITY_DN21082_c0_g2_i6.p1, TRINITY_DN21082_c0_g1_i3.p1) matched cholecystokinin/sulfakinin receptors with low E-values in other species, suggesting their relation to *Aplysia* CCK receptors, tentatively named apCCKR1 and apCCKR2, respectively. The other two sequences (asmbl_32627.p1 in AplysiaTools, XP_005090536.1 in NCBI) did not return significant matches with known proteins (Data S6). Using Pfam (http://pfam.xfam.org/search#tabview=tab1), we classified the first as Class-A GPCRs (rhodopsin family), and the second one as Serpentine type 7TM GPCR chemoreceptor Srw, named Aplysia Class-A_GPCR1 and Aplysia Srw_GPCR1 (Data S6).

The open reading frames (ORFs) of the apCCK precursor, and the putative apCCK receptors were obtained using ORF Finder (https://www.ncbi.nlm.nih.gov/orffinder/). For the apCCK precursor, the putative signal peptide was predicted using SignalP-5.0 (http://www.cbs.dtu.dk/services/SignalP/) and the putative peptides encoded by the apCCK precursor were predicted using NeuroPred (http://stagbeetle.animal.uiuc.edu/cgi-bin/neuropred.py). We also compared the apCCK precursor and neuropeptides with those of other species using BioEdit software. For the putative apCCK receptors, transmembrane domains were predicted using DeepTMHMM 1.0.12 (https://dtu.biolib.com/DeepTMHMM). The phylogenetic trees of sequences from different species were constructed by MEGA X software (https://www.megasoftware.net/) using alignment by CLUSTALW,^138^ and the maximum likelihood method with 1000 replicates. We used LG + G + F model to generate a balanced tree including putative CCK receptors (Figure S14B), and used JTT + G + F model to generate the CCK receptor phylogenetic tree (Figure S15). The selection of the models was based on the results of “find best DNA/protein models” of MEGA analysis.

#### Cloning of *Aplysia* CCK precursor and putative receptors

##### RNA extraction.

*Aplysia* buccal, cerebral, pleural-pedal and abdominal ganglia were dissected out and combined, then maintained in artificial seawater containing the following (in mM): 460 NaCl, 10 KCl, 55 MgCl_2_, 11 CaCl_2_, and 10 HEPES buffer, pH 7.6. RNA was prepared from the *Aplysia* ganglia using the TRIzol reagent method. Specifically, the dissected ganglia were placed into 200 μL TRIzol (Sigma, T9424) and stored at −80°C until use. The frozen ganglia were thawed in TRIzol and homogenized with a plastic pestle. TRIzol was added to a total volume of 1 mL and the preparation was incubated at room temperature for 10 min. Then, 200 μL chloroform was added, and the solution was mixed thoroughly by shaking, and left to stand on ice for 15 min. The solution was centrifuged (12,000 × g, 4°C, 15 min). The upper aqueous phase was transferred into a clean tube and an equal volume of isopropanol was added. The tube was shaken gently and left to stand at −20°C for 2 h. The tube was centrifuged again (12,000 × g, 4°C, 15 min), the supernatant was discarded, and 1 mL of 75% ethanol/water was added. The centrifuge tube was shaken gently to suspend the pellet, and then was centrifuged (12,000 × g, 4°C, 10 min). The supernatant was discarded and the precipitant was dried at room temperature for 5–10 min. Finally, 20 μL of nuclease-free water was added to dissolve the RNA pellet, and RNA concentration was obtained using a Nanodrop ND-1000 spectrophotometer (Thermo Fisher Scientific).

##### Reverse transcription.

5 μL of the above RNA at 200 ng/μL, 4 μL of 5 × PrimeScript RT Master Mix and 11 μL DEPC H_2_O were used to synthesize cDNA by reverse transcription using PrimeScript RT Master Mix Kit (Takara, RR036A) according to the kit instructions. Specifically, reverse transcriptase polymerase chain reaction (RT-PCR) was performed at 37°C for 15 min and reverse transcriptase was inactivated at 85°C for 5 s. The synthesized first-strand cDNA served as a template for PCR.

##### PCR.

The synthesized cDNA above was used as templates and the predicted ORFs of the apCCK precursor or apCCK receptors were amplified by PCR. Each pair of specific primers was designed (see Data S1) based on protein coding sequences for the apCCK precursor and putative receptors. The PCR reaction was performed with 98°C/2 min pre-denaturing, 98°C/10 s denaturing, annealing temperature (depending on the specific primers (see Data S1))/15 s annealing, 72°C/30 s extension and 72°C/5 min re-extension for 35 cycles. The PCR products were subcloned into vector pcDNA3.1 (+) and sequenced to ensure the sequence was correct.

#### *In situ* hybridization

*In situ* hybridization was performed as described previously.^58,139–141^ Animals were fed *ad libitum* without fasting before sacrificing to obtain isolated ganglia. We used the full sequence of apCCK precursor (459 bp in length), the full sequence of apCCKR1 (1323 bp in length) and partial sequence of apCCKR2 (1134 bp in length, from 301 to 1725 bp) to make digoxigenin-labeled RNA probes. For the synthesis of RNA probes, the corresponding region of these cDNAs was amplified using primers (see Data S1) bearing restriction enzyme sites, with I-5 2×High-Fidelity Master Mix (TSINGKE, TP001). The resulting PCR amplicon was purified from an agarose gel using a Zymo DNA clean and concentrator (DCC) kit with agarose dissolving buffer. The purified PCR product and the pSPT18 vector were each double digested with restriction enzymes and repurified with a DCC kit. Then the restriction enzyme-digested target cDNA was ligated to the restriction enzyme-digested pSPT18 vector. After a series of molecular cloning procedures, the pSPT18 plasmids containing the target cDNAs was obtained. A region of these plasmids containing both the cDNAs as well as the T7 and SP6 promotors necessary for riboprobe synthesis was amplified by PCR using 10 μM of each of the following primers: 5^′^-TGCTTCAGTAAGCCAGATGC-3^′^, reverse 5^′^-GCTACGTGACTGGGTCATGG-3’. The amplified product was purified by DCC kit and sequenced by Sanger sequencing using the above primers to confirm the desired sequence. Next, digoxigenin-labeled RNA probes were synthesized using a DIG RNA labeling Kit (SP6/T7) (Roche, 11175025910), following the manufacturer’s instructions.

#### Immunohistochemistry

For immunohistochemistry experiments, a polyclonal antibody against apCCK1 (pQGAWSYDYGLGGGRF-NH_2_) was raised by ChinaPeptides Co.Ltd (Shanghai). Specifically, apCCK1 was conjugated to keyhole limpet hemocyanin (KLH) by introducing a cysteine residue at the N terminus of apCCK1—a well-established strategy for antibody production. The sulfhydryl group (-SH) of the added cysteine was then directly coupled to KLH. Antigen-specific antibodies were affinity-purified from the antiserum with the corresponding peptide conjugated to CNBr (Cyanogen Bromide)-activated agarose beads. Immunohistochemistry in whole mount was performed as described previously.^13,59,142^ Animals were fed *ad libitum* without fasting before sacrificing to obtain isolated ganglia. The tissue was fixed in a buffer (4% paraformaldehyde, 0.2% picric acid, 25% sucrose, and 0.1 M NaH_2_PO_4_, pH 7.6), for either 3 h at room temperature or overnight at 4°C. All subsequent incubations were done at room temperature. The tissue was washed with PBS, and was permeabilized and blocked by overnight incubation in blocking buffer (10% normal goat serum, 2% Triton X-100, 1% BSA, 154 mM NaCl, 50 mM EDTA, 0.01% thimerosal or 0.01% sodium azide, and 10 mM Na_2_HPO_4_, pH 7.4). The primary antibody was diluted 1:5000 in blocking buffer and incubated with the tissue for 4–7 days. The tissue was then washed twice per day for 2–3 days with washing buffer (2% Triton X-100, 1% BSA, 154 mM NaCl, 50 mM EDTA, 0.01% thimerosal or 0.01% sodium azide, and 10 mM Na_2_HPO_4_, pH 7.4). After washing, the tissue was incubated with a 1:500 dilution of secondary antibody (Alexa Fluor 594 goat anti-rabbit; Invitrogen: A-11012) for 2–3 days and then washed again two times with washing buffer for 1 day and four times with storage buffer (1% BSA, 154 mM NaCl, 50 mM EDTA, 0.01% thimerosal or 0.01% sodium azide, and 10 mM Na_2_HPO_4_, pH 7.4) for 1 day. The tissue was observed and photographed under fluorescence microscope or a confocal microscope (Zeiss LSM 900). Images were processed with ImageJ.

#### Backfills in conjunction with immunohistochemistry

Biocytin backfills of nerves and nerve branches were performed similarly to previous studies.^110,143^ Following anesthesia and initial dissection, the preparation of nerve or CBC and buccal ganglion or peripheral tissue was pinned on a silicon elastomer (Sylgard, Dow Chemical) lined dish. The cut nerve ending was placed in a small chamber made from the combination of a small plastic conical tube and silicon grease (High Vacuum Grease, Dow Corning). A solution of 10% of a saturated Fast Green FCF (Sigma) solution in dH_2_O was placed in the chamber to osmotically shock the nerve and to verify there were no leaks. This solution was replaced with 20 μL of 5% Biocytin (Sigma) in dH_2_O. The rest of the preparation was immersed in ASW. Following incubation at 4° C for 48 h, the tissues were rinsed in PBSe, and fixed for immunohistochemistry as described above. To visualize the biocytin, the tissues were incubated in Streptavidin-Alexa Fluor 488 (Thermo Fisher Scientific) at 1:250, at the same step as the secondary antibody in the immunohisto-chemistry protocol (see above). In all cases, the photographs are flattened images of stacks of confocal images taken of whole mounts of the walls of the gastrointestinal tract.

#### Ligations of the EN

The esophageal nerve (EN) was ligated to determine the source of apCCK-IR fibers in the nerve. An anesthetized *Aplysia* was pinned by its tentacles in an inclined dissection tray. The head remained exposed, but the rest of the animal was enclosed in a plastic bag containing cooled, aerated ASW. A short (1–1.5 cm) incision was made just above or in the foot to expose the caudal-ventral side of the buccal mass. The EN was ligated unilaterally about halfway between the buccal ganglion and the first EN branches with surgical silk (Ethicon 6.0; eSutures, Mokena, IL). In all preparations, the nerve was effectively severed. The incision was closed with sutures (Ethicon 2.0; eSutures, Mokena, IL) and sealed with cyanoacrylate glue (Krazy Glue; VWR, Radnor, PA). The animal was returned to the tank following the procedure. All animals recovered and started eating after 1 day. Initial experiments revealed no changes in apCCK-IR after 1-day survival and inconsistent results at 2 days of survival, so 3 days of survival (*n* = 5) was chosen for these experiments. Animals were re-anesthetized and the ENs and attached buccal ganglion were fixed overnight in 4% paraformaldehyde (EMS, Hatfield, PA), 0.2% picric acid, and 25% sucrose in 0.1 M Na_2_HPO_4_ prior to immunohistochemistry.

#### Structural characterization of apCCK peptides

##### Peptide measurements by LC-MS/MS

To structurally characterize actual apCCK precursor gene products in the *Aplysia* CNS, peptide extracts from individual ganglia and the EN were sequenced *de novo* by LC-MS/MS.^144,145^ Isolated and desheathed ganglia and EN extracts were homogenized using Precellys bead beater set to the “Soft” method (3 × 15 s, 5800 rpm protocol). Homogenates were collected and centrifuged at 14,000 × g for 15 min to precipitate tissue debris. Resulting supernatants were transferred into another set of vials, dried using a speed-vac, and the dry vial contents were reconstituted in 15 μL of 0.1% formic acid (FA). The insoluble portion was separated by another centrifugation at 14,000 rpm for 15 min and the resulting supernatant was used for measuring peptides through LC-MS/MS.

Native and synthetic apCCKs were measured using Bruker nanoElute LC hyphenated via CaptiveSpray nanosource with external oven to Bruker timsTOF Pro mass spectrometer (Bruker Daltonics, Billerica, MA). Samples were injected onto a Bruker PepSep Fifteen column (75 μm ID, 1.9 μm particle size, 150 mm length) equipped with a precolumn trap for desalting. The separation was performed at a uniform flow rate of 300 μL/min and a temperature of 40°C. The mobile phase consisted of solvent A (0.1% formic acid) and solvent B (acetonitrile with 0.1% formic acid). A gradient of solvent B was applied, starting from 2% and increasing to 10% in 3 min and then to 50% over next 75 min, followed by a rapid ramp to 90% for wash, and finally equilibration with starting conditions. The positive ion mass spectra were acquired in Parallel Accumulation Serial Fragmentation (PASEF) mode with a cycle time of 1.1 s and 1/k0 range 0.6–1.6 V s/cm^2^.

##### Peptide identification and PTM assignment

The acquired PASEF spectra were processed for extracted ion chromatogram with m/z values for predicted apCCKs and corresponding fragmentation patterns and 1/k0 values were compared to those of synthetic standards. Fragmentation pattern interpretation was performed using PEAKS Online 11 (Bioinformatics Solutions) against a custom list of select *Aplysia* prohormones publicly available in Uniprot. Search parameters included variable PTMs amidation, pyro-Glu from E and Q, sulfation and phosphorylation as PTMs with maximum four PTMs per peptide. Precursor and fragment ion accuracy were set as 15 ppm and 0.05 Da, respectively. Search results were filtered as 1% false discovery rate for peptide sequences and exported in .*csv* format for further analysis.

#### Behavioral experiments

Behavioral experiments were conducted in two batches, each using animals from separate shipments. In the first batch (Figure 2), animals were larger, with an average body weight of 93.3 ± 4.8 g, and were food-deprived for 7 days prior to testing. In the second batch (Figure S9), animals were smaller (average weight: 70.6 ± 4.0 g) and were food-deprived for 5 days to account for their reduced size. In each experiment, two weight-matched animals were housed in separate round net cages (17 cm diameter and 17 cm height) that were placed in a 500 L tank filled with circulating aerated ASW (14°C–16°C). To ensure that the animals were responsive to food, they were first pretested with a small piece of seaweed. After this pretest, in each pair, one animal was injected (into the hemocoel using a syringe needle) with apCCKs freshly dissolved in 1.5 mL ASW, whereas the other was injected with 1.5 mL of ASW. Three minutes later, the feeding session commenced. Animals were hand-fed seaweed (Shirako, Tokyo, Japan) cut in 1 × 10 cm strips, weighing ~0.1 g, increasing 10-fold when hydrated. This hand-feeding approach has been used in prior study of *Aplysia* feeding behavior.^146,147^ The consumed seaweed amount was converted to hydrated weight and normalized to the animal’s body weight. Each strip of seaweed was delivered to the mouth area with forceps. Feeding was terminated after 1 h or when animals stopped ingesting seaweed for 3 min. The experimenter who fed the animals did not know which animal was injected with apCCKs.

#### Receptor activation: IP1 accumulation assay

CHO-K1 cells were cultured in F-12K medium (Gibco, 21127–022) with 10% fetal bovine serum at 37°C in 5% CO_2_. Before transfection, cells were grown in 35 mm diameter tissue culture-treated dishes to 85% confluence. For transfection, 1.5 μg apCCKR plasmids or control plasmids (in pcDNA3.1(+) vector) and 1.5 μg promiscuous Gαq-family protein (in pcDNA3.1(+) vector) were mixed with 200 μL Opti-MEM (Gibco, 11058021), and 8 μL Turbofect (Thermo Fisher Scientific, R0531). This promiscuous Gαq protein contains substitutions to increase promiscuity,^148^ and could bind to most GPCRs, ensuring an IP1 response when a potential ligand binds to its specific GPCR, irrespective of the GPCR’s normal association with any specific Gα proteins. The DNA mixture was incubated at room temperature for 15 min and then added dropwise to the cells in F-12K medium with 10% FBS (no antibiotics), followed by incubation at 37°C in 5% CO_2_ overnight. The next day, cells were detached using trypsin and reseeded in white 96-well half-area tissue culture-treated plates (Corning, 3688) at a density of 20,000 cells per well in F-12K and 10% FBS, and incubated at 37°C in 5% CO_2_ overnight. Activation of putative apCCK or related receptor was detected by monitoring IP1 accumulation using IPOne Detection Kit (Cisbio, 62IPAPEB), as previously described.^64,65,141,149,150^

#### EN stimulation and releasates sampling

Electrical stimulation of the EN was performed at 2 Hz, 3 ms, 15 V. SPE C_18_ ZipTip pipettes (Millipore Co., Billerica, MA) were “wetted” by aspiration with 50% ACN (Acetonitrile) and equilibrated in 50% ASW immediately prior to use. The wetted pipettes were connected via Tygon tubing (Saint-Gobain Performance Plastics Co., Paris, FR) to a Kd Scientific syringe pump and Hamilton gastight syringe (Hamilton Company, Reno, NV). The ZipTip was mounted on a micromanipulator for precise positioning on the rostral surface of the buccal hemi-ganglion. The inner diameter of the pipette tip measured ~500 μm and was sufficient to cover the majority of the surface area of buccal hemi-ganglion. Collection was performed by running the pump in negative mode, pulling extracellular samples across the C_18_ material. Pipette collections, both prestimulation and stimulation-onset, lasted ~5.5 min, and the rate of the syringe pump was set so that ~10 μL of releasates could be collected. Following each sample collection, the ZipTip was removed, cleared of ASW, and aspirated with 0.1% formic acid in purified H_2_O (both Optima LC/MS grade, Fisher Chemical) to rinse salts. At this point, Ziptips with peptides bound to C_18_ were shipped overnight from Elizabeth C. Cropper’s lab at the Icahn School of Medicine at New York (the site of releasates collection) to Jonathan V. Sweedler’s lab at the University of Illinois at Urbana/Champaign for MS analysis.

#### Analysis of apCCKs in EN releasates by MALDI-TOF MS

To characterize apCCKs in the *Aplysia* EN releasates, we used matrix-assisted laser desorption/ionization (MALDI) time-of-flight (TOF) MS due to very small sample volume. Peptides were eluted from ZipTips with 4 μL of 70% ACN onto MALDI target plates, co-mixed with DHB matrix (DHB: 2,5-dihydroxybenzoic acid, 20 mg/mL deionized water) and allowed to dry at room temperature prior to measurements.

Peptide profiles were measured using ultrafleXtreme MALDI-TOF/TOF mass spectrometer (Bruker Daltonics, Billerica, MA) equipped with a Smartbeam II frequency tripled Nd:YAG solid-state laser. Mass spectra were manually acquired in positive reflectron mode with external calibration using Bruler Peptide Mix II as calibrant. Signals from 500 to 4000 laser shots fired at 2000 Hz frequency at multiple locations within each sample spot were summed into representative sample spectrum. Obtained mass spectra were processed using flexAnalysis 3.4 software (Bruker Daltonics, Billerica, MA). To aid interpretation of the MS results, putative peptides encoded by the apCCK precursor were predicted using the Neuropred prediction tool. Location of the putative signal peptide cleavage site was inferred using SignalP-5.0 model. Assignment of detected peptides was performed by mass match to predicted peptides as well as to peptides identified by LC-MS/MS in the buccal ganglion and nerve extracts within reasonable mass accuracy.

#### Electrophysiology

Intracellular and extracellular recordings were made as described previously.^57–59,61,151^ To prevent movements of ganglia caused by myoactive apCCK (especially [sY8]-apCCK1 and [sY6, sY8]-apCCK1) during bath application, we treated the ganglia with 0.8% protease IX for 1–2 min prior to desheathing. Subsequently, ganglia were desheathed, transferred to a recording chamber containing ~1.5 mL of ASW (460 mM NaCl, 10 mM KCl, 11mM CaCl_2_, 55 mM MgCl_2_, and 10 mM HEPES, pH 7.6), continuously perfused at 0.3 mL/min, and maintained at 14°C–17°C. The experiments examining the effects of apCCKs on excitability of B20/B34/B61/62/B65/B64 neurons were performed in a high divalent saline (368 mM NaCl, 8 mM KCl, 13.8 mM CaCl_2_, 115 mM MgCl_2_, and 10 mM HEPES, pH 7.6), which increases the spiking threshold of neurons and therefore curtails polysynaptic influences. Intracellular recordings were obtained using 5–10 MΩ sharp microelectrodes filled with an electrolyte (0.6 M K_2_SO_4_ plus 60 mM KCl). Extracellular recordings were acquired from polyethylene suction electrodes. Grass S88 and WPI Pulsemaster A300 stimulators were used to provide timing signals for intracellular and extracellular stimulation. To determine the effects of various forms of apCCKs on *Aplysia* feeding programs, we performed experiments with the cerebral and buccal ganglia. Freshly made apCCK solution in ASW was perfused into the recording chamber. Feeding-motor programs were elicited by stimulation of command-like neuron CBI-2 at 8–10 Hz or stimulation of the EN at 2 Hz, 3 ms, 15 V, and were monitored by bursting activity of the I2 nerve of the buccal ganglion.^58,59,61^ Typically, we recorded from motor neuron B8, and one interneuron, e.g., B40 or B20. To determine effects of apCCKs on synaptic strength, we stimulated presynaptic neuron, CBI-2, B34 (plus 1 nA current) or B65 at 10 Hz for 2 s for several trials and waited 1 min between each trial. The stimulation duration for each trial was set at 2 s because this allows the expression of synaptic facilitation without evoking too many spikes. Typically, the amplitude of the last EPSP at each concentration of peptides was quantified unless otherwise stated.

#### Single-cell RNA-seq using SMART-seq

The sheath covering the ganglia was removed. The neurons were initially identified based on their anatomical location and electrophysiological properties. Following identification, fast green buffer was injected into the target neuron to enhance visibility under microscopic observation. The ganglion was fixed using ethyl alcohol for approximately 1–2 min. Subsequently, the cells surrounding the target neuron were carefully removed using forceps. The target neuron was then isolated carefully using scissors and forceps. Once isolated, the single neuron was transferred into a tube containing RNAase inhibitor and lysis buffer (Takara Bio, Cat. No. 635013). The tubes were rapidly placed into liquid nitrogen for flash freezing and subsequently stored at −80°C. Single-cell RNA sequencing (SMART-seq) was performed by Quintara Biosciences Co., Ltd. or Annoroad Gene Technology (Beijing) Co., Ltd.

We aligned sequencing data from multiple batches of sequencing using software HISAT2 and the reference genome AplCal3.0 (GCF_000002075.1), and obtained read counts for each gene sample using the featureCounts tool.^152,153^ Subsequently, we employed “sva” package in R version 4.1.1 to eliminate batch effects from the read counts of the multi-batch samples.^154,155^ The “relative counts” in the figures refer to the read counts of each sample after removal of batch effects.

### QUANTIFICATION AND STATISTICAL ANALYSIS

Dose-response curves and bar graphs for experimental data were plotted using Prism software (GraphPad version 8). Data are presented as mean ± SEM of at least three independent experiments, except that LogEC_50_ values reported in the main text are the mean ± standard deviation (SD) from at least three independent experiments. Statistical tests were performed using Prism software. They included Student’s t-test, one-way or two-way ANOVA, as appropriate. Data that showed significant effects in one-way ANOVA or two-way ANOVA were further analyzed in post-hoc individual comparisons with appropriate corrections. Electrophysiological recordings were digitized online using AxoScope software version 10.7 (Molecular Devices, LLC, Sunnyvale, CA) and were plotted by CorelDraw version 2018 (Corel Corp., Ottawa, Canada).

## Supplementary Material

SI1

SI2

SI3

SI4

SI5

SI6

SI7

SI8

SI9

SI10

Supplemental information can be found online at https://doi.org/10.1016/j.celrep.2025.116049.

## Figures and Tables

**Table:** KEY RESOURCES TABLE

REAGENT or RESOURCE	SOURCE	IDENTIFIER
Antibodies		
apCCK1 antibody	ChinaPeptides Co. Ltd	RRID: AB_3711288
Alexa Fluor 594 goat anti-rabbit	Invitrogen	CAS NO. A-11012; RRID: AB_2534079
Chemicals, peptides, and recombinant proteins		
apCCK	GuoPing Pharmaceutical Co., Ltd; Sangon Biotech (Shanghai) Co., Ltd; The lab of James W. Checco at the University of Nebraska-Lincoln	N/A
Fmoc-Tyr (SO_3_nP)	Novabiochem	CAS NO. 8.52347
NovaPEG rink amide resin	Novabiochem	CAS NO. 855047
NaCl	Sigma-Aldrich	CAS NO. S5886
MgCl_2_	Sigma-Aldrich	CAS NO. 208337
KCl	Sigma-Aldrich	CAS NO. P9541
CaCl_2_	Sigma-Aldrich	CAS NO. C4901
HEPES	Sigma-Aldrich	CAS NO. 54457
TRIzol	Sigma-Aldrich	CAS NO. T9424
chloroform	Sigma-Aldrich	CAS NO. 319988
isopropanol	Sigma-Aldrich	CAS NO. 563935
ethanol	Sigma-Aldrich	CAS NO. 493511
paraformaldehyde	Electron Microscopy Sciences	CAS NO. 15710
picric acid	Sigma-Aldrich	CAS NO. 41506
sucrose	Sigma-Aldrich	CAS NO. S5016
NaH_2_PO_4_	Sigma-Aldrich	CAS NO. S0751
Normal goat serum	Sigma-Aldrich	CAS NO. NS02L
Triton X-100	Sigma-Aldrich	CAS NO. 93443
BSA	Sigma-Aldrich	CAS NO. A1933
EDTA	Sigma-Aldrich	CAS NO. E8008
sodium azide	Sigma-Aldrich	CAS NO. 8.22335
Fast Green FCF	Sigma-Aldrich	CAS NO. F7252
Biocytin	Sigma-Aldrich	CAS NO. B4261
Streptavidin-Alexa Fluor 488	Thermo Fisher Scientific	CAS NO. S32354
formic acid	Sigma-Aldrich	CAS NO. F0507
trifluoroacetic acid	Sigma-Aldrich	CAS NO. 8.08260
triisopropylsilane	Sigma-Aldrich	CAS NO. 8.41359
acetonitrile	Sigma-Aldrich	CAS NO. 34851
F-12K medium	Gibco	CAS NO. 21127-022
Fetal bovine serum	Gibco	CAS NO. A5670701
Opti-MEM	Gibco	CAS NO. 11058021
Turbofect	Thermo Fisher Scientific	CAS NO. R0531
protease IX	Sigma-Aldrich	CAS NO. P6141
lysis buffer	Takara Bio	CAS NO. 635013
Critical commercial assays		
PrimeScript^™^ RT Master Mix Kit	Takara	CAS NO. RR036A
I-5^™^ 2×High-Fidelity Master Mix	TSINGKE	CAS NO. TP001
DNA clean and concentrator (DCC) kit	ZYMO	CAS NO. D4014
DIG RNA labeling Kit	Roche	CAS NO. 11175025910
IPOne Detection Kit	Cisbio	CAS NO. 62IPAPEB
Single-cell RNA sequencing	Quintara Biosciences Co., Ltd/Annoroad Gene Technology (Beijing) Co., Ltd	N/A
Deposited data		
apCCK precursor sequence	This paper	NCBI: PV530374
apCCKR1 sequence	This paper	NCBI: PV530375
apCCKR2 sequence	This paper	NCBI: PV530376
B20 and B34 RNA sequencing data	This paper	NCBI: GSE296741
Experimental models: Cell lines		
CHO-K1	Procell Life Science &Technology	CAS NO. CL-0062; RRID: CVCL_0214
Experimental models: Organisms/strains		
*Aplysia californica*	Marinus Scientific/RSMAS National Resource for Aplysia	RRID: nif-0000-00530
Oligonucleotides		
ATGGAACCCCAACTTCTCAGCGTCGTCATT	Sangon Biotech	N/A
TCATGACGTAATCTCATTCTCGGTGGCGTC	Sangon Biotech	N/A
ATGACCTGGGAAGTGTGCGAGCAGGAGTTC	Sangon Biotech	N/A
CTAACGGTCTCTCTTCCAGGTGATGGTGTA	Sangon Biotech	N/A
ATGGCAGTCCTAGGAGACGTCTCGAGGAATGC	Sangon Biotech	N/A
TCAGTCGGAAGCGCTGGTGGTATCCTCTTCC	Sangon Biotech	N/A
ATGGCTGCTGGGATCACGAACC	Sangon Biotech	N/A
TCAGGTGTCCGAGTGACTGCTG	Sangon Biotech	N/A
ATGGAAGTGACAACATCCACAGTGTCAAAAC	Sangon Biotech	N/A
CTAATGCCAGTTTCCTTTTCCTTCGACCTT	Sangon Biotech	N/A
CCGGAATTCATGGAACCCCAACTTCTCA	Sangon Biotech	N/A
CCCAAGCTTTCATGACGTAATCTCATTCTC	Sangon Biotech	N/A
CCGAATTCATGACCTGGGAAGTGTG	Sangon Biotech	N/A
CGGGATCCCTAACGGTCTCTCTTCC	Sangon Biotech	N/A
CCGGAATTCATGTGTGTCTTCATCC	Sangon Biotech	N/A
AACAAGCTTTCAGTCGGAAGCGCTGGT	Sangon Biotech	N/A
TGCTTCAGTAAGCCAGATGC	Sangon Biotech	N/A
GCTACGTGACTGGGTCATGG	Sangon Biotech	N/A
Recombinant DNA		
pcDNA3.1(+) vector	NovoPro	CAS NO. V012531
pSPT18 vector	NovoPro	CAS NO. V013001
Software and algorithms		
ORF Finder	https://www.ncbi.nlm.nih.gov/orffinder/	N/A
SignalP-5.0	DTU Health Tech	http://www.cbs.dtu.dk/services/SignalP/
NeuroPred	UIUC Center for Neuroproteomics	http://stagbeetle.animal.uiuc.edu/cgi-bin/neuropred.py
BioEdit	https://thalljiscience.github.io/	N/A
DeepTMHMM1.0.12	DTU Health Tech	https://dtu.biolib.com/DeepTMHMM
Pfam	Mistry et al.^137^	http://pfam.xfam.org/search#tabview=tab1
MEGA X	Kumar et al.^138^	https://www.megasoftware.net/
flexAnalysis 3.4	Bruker Daltonics	N/A
HISAT2	https://daehwankimlab.github.io/hisat2/	N/A
GraphPad version 8	GraphPad Software	https://www.graphpad.com/
AxoScope software version 10.7	Molecular Devices	N/A
CorelDraw version 2018	Corel Corp	N/A

## Highlights

- *Aplysia* CCK (apCCK) precursor, multiple apCCKs, and two receptors are characterized
- apCCKs are expressed in both the CNS and the gut and suppress food intake *in vivo*
- Peripheral apCCK-expressing cells project to the CNS and trigger central apCCK release
- apCCKs modulate feeding circuits via both intrinsic and synaptic plasticity mechanisms
