# Supplementary material for: Synaptic and intrinsic plasticity mediated by CCK-type signaling coordinates behavioral changes during motivational state shifts
Source: Cell Rep. Author manuscript; Available in PMC 2025 Oct 18. (PMC12535377; doi:10.1016/j.celrep.2025.116049)
Supplement: SI1 [file NIHMS2116669-supplement-SI1.pdf]

Cell Reports, Volume *44*

Supplemental information

Synaptic and intrinsic plasticity mediated by CCK-type signaling coordinates behavioral changes during motivational state shifts

Guo Zhang, Xue-Ying Ding, Elena V. Romanova, Cui-Ping Liu, Michael A. Barry, Alisha Doda, Qian-Xue Chen, Carrie Reaver, Qing-Chun Jin, Stanislav S. Rubakhin, Fan Li, Yu-Fei Jin, Yan-Sheng Kan, Yu-Ling Liu, Shi-Qi Guo, Ying-Yu Xue, Yu-Shuo Mei, Ping Fu, Ju-Ping Xu, Rui-Ting Mao, Cheng-Yi Liu, Yan-Chu-Fei Zhang, Yi-Long Zhang, Jian-Hui Chang, Shao-Qian Wu, Hui-Ying Wang, Wei-Jia Liu, Ping Chen, Zhen Zhou, Hai-Bo Zhou, Quan Yu, James W. Checco, Jonathan V. Sweedler, Elizabeth C. Cropper, and Jian Jing

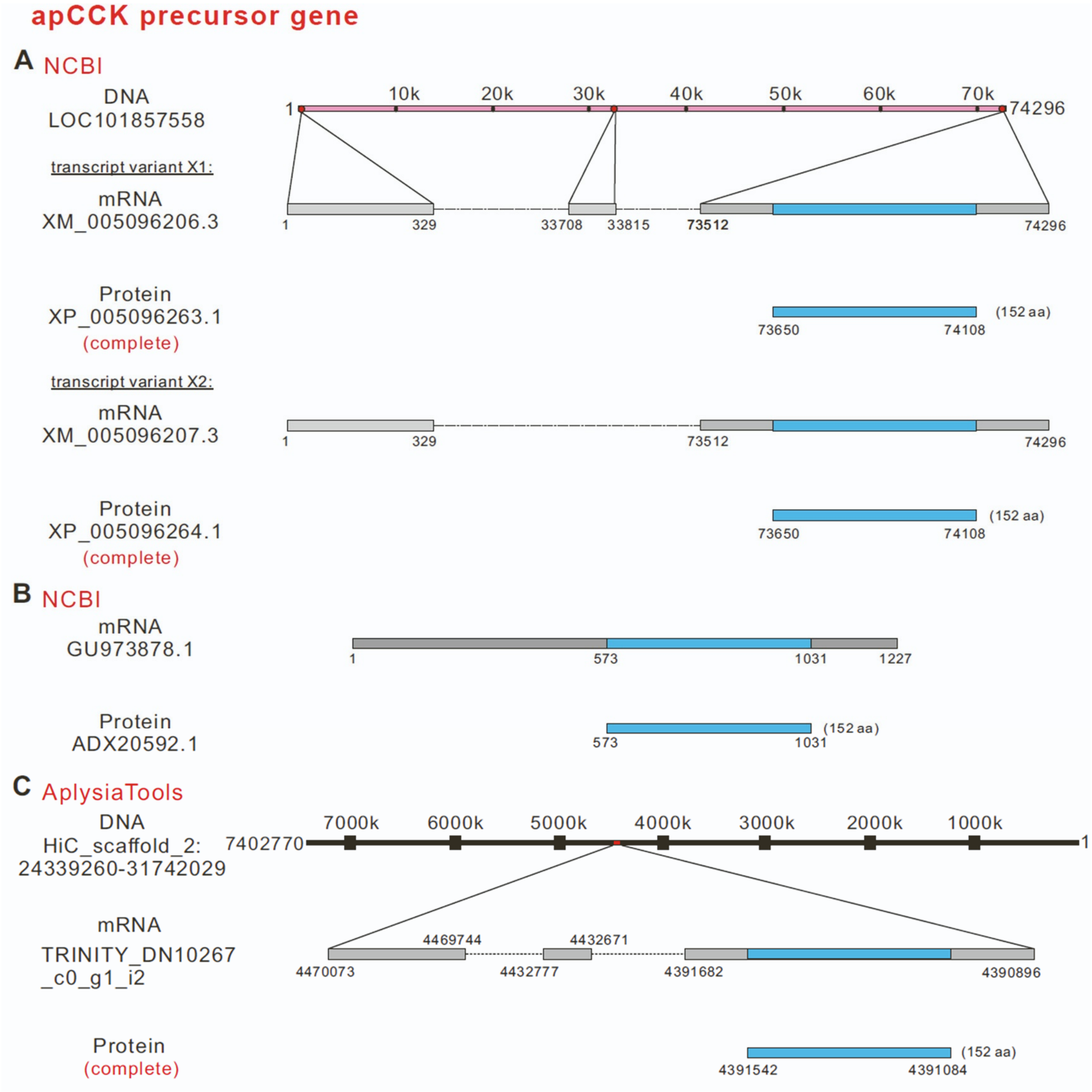

Figure S1: Gene expression mapping of *Aplysia CCK precursor*. Related to Figure 1. (A) The gene (LOC101857558) corresponds to two transcript variant mRNAs, one is XM_005096206.3, with two introns between 329 to 33708 bp and between 33815 to 73512 bp, which produces an uncharacterized protein XP_005096263.1. The other one is XM_005096207.3, with an intron between 329 to 73512 bp, which produces an uncharacterized protein XP_005096264.1. The two proteins are identical, and are similar to CCK precursors from other mollusks. **(B)** A mRNA from NCBI (GU973878.1), named *Aplysia* betsin mRNA, complete CDS, was submitted by Moroz et al, but the related paper was not published. It produces a protein (ADX20592.1), which is identical to XP_005096263.1 and XP_005096264.1 in (A). **(C)** DNA HiC_scaffold_2:24339260-31742029 (Note that the nucleotide number on top starts from the right) from *Aplysia* gene nucleotide databases (the AplysiaTools) expresses the similar mRNA as in (A) and (B), and the protein generated from this mRNA is the same as those in (A, B). Note that the numbers for the proteins refer to base pairs of corresponding mRNAs, not amino acids.

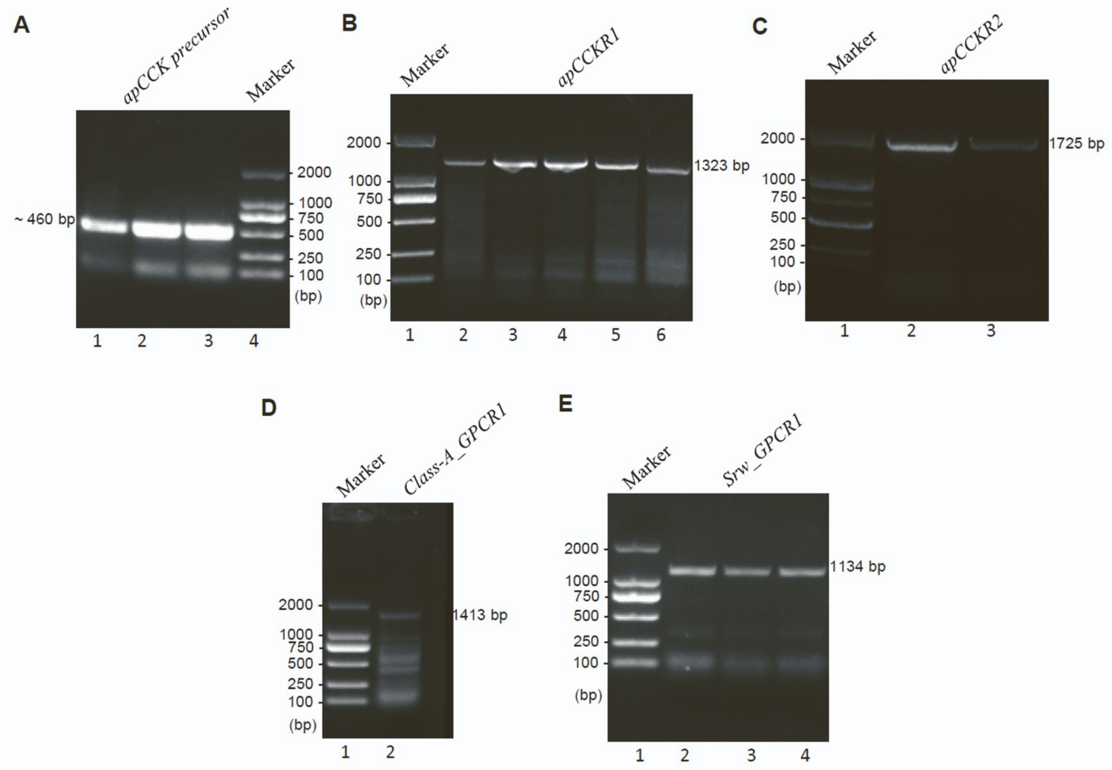

Figure S2: Cloning of *Aplysia* CCK precursor and putative receptors. Related to Figure 1. (A) A PCR product for *Aplysia CCK precursor* (*apCCK pre*) gene with a length of 459 bp. **(B)** A PCR product for a putative receptor (*apCCKR1*) with a length of 1323 bp. **(C)** A PCR product for a putative receptor (*apCCKR2*) with a length of 1725 bp. **(D)** A PCR product for a putative receptor (*Class-A_GPCR1*) with a length of 1413 bp. **(E)** A PCR product for a putative receptor (*Srw_GPCR1*) with a length of 1134 bp. All the above mRNA sequences have been verified by sequencing.

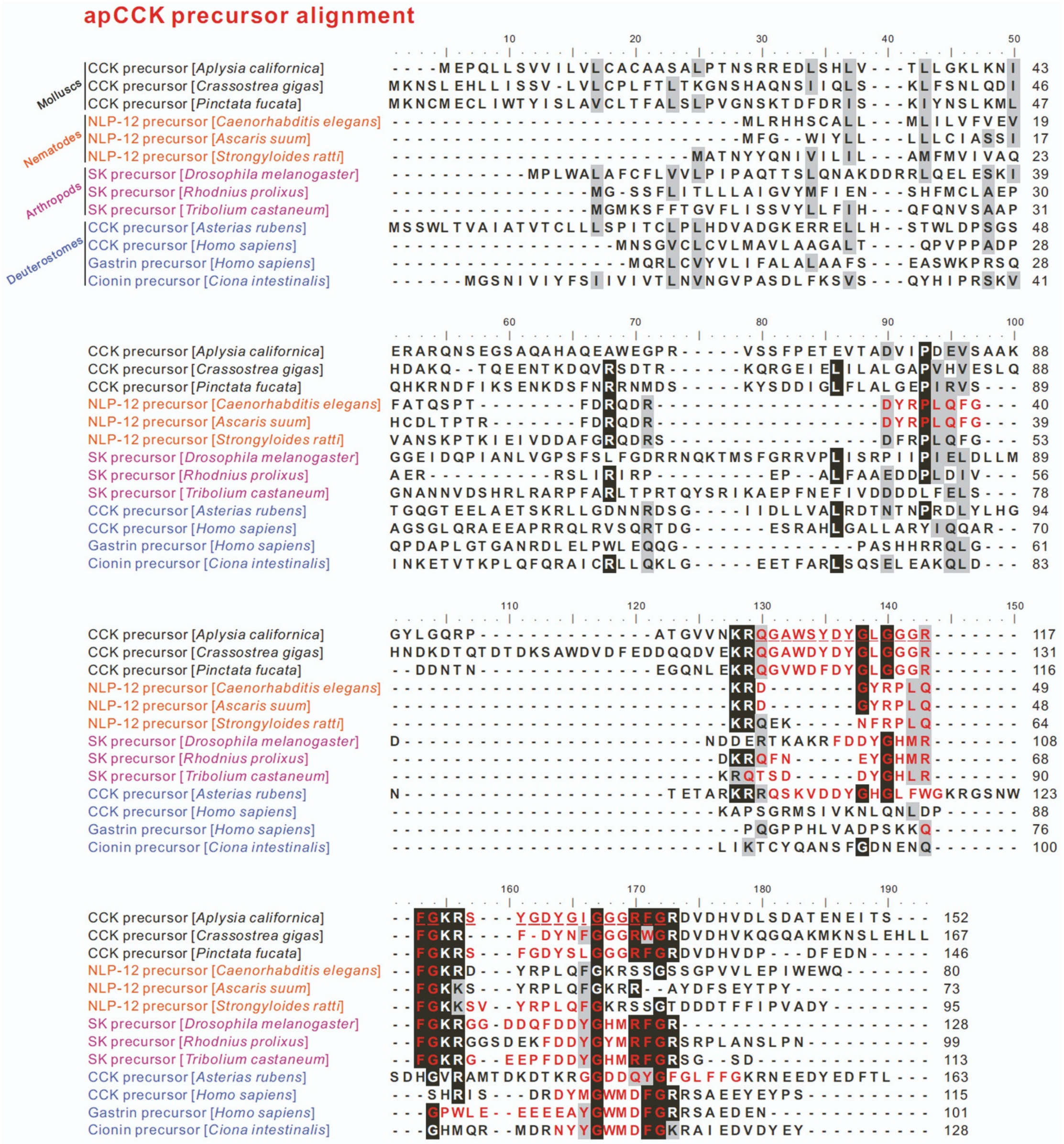

Figure S3: Comparison of *Aplysia* CCK precursor vs. similar precursors in protostomes and deuterostomes using BioEdit (ClustalW Multiple Alignment-Graphic View). Related to Figure 1. See Dataset S3 for the sources of the sequences used in this figure. The underlined resides (also shown in red) are the predicted *Aplysia* CCK peptide sequences. The CCKs generated from other species are also shown in red.

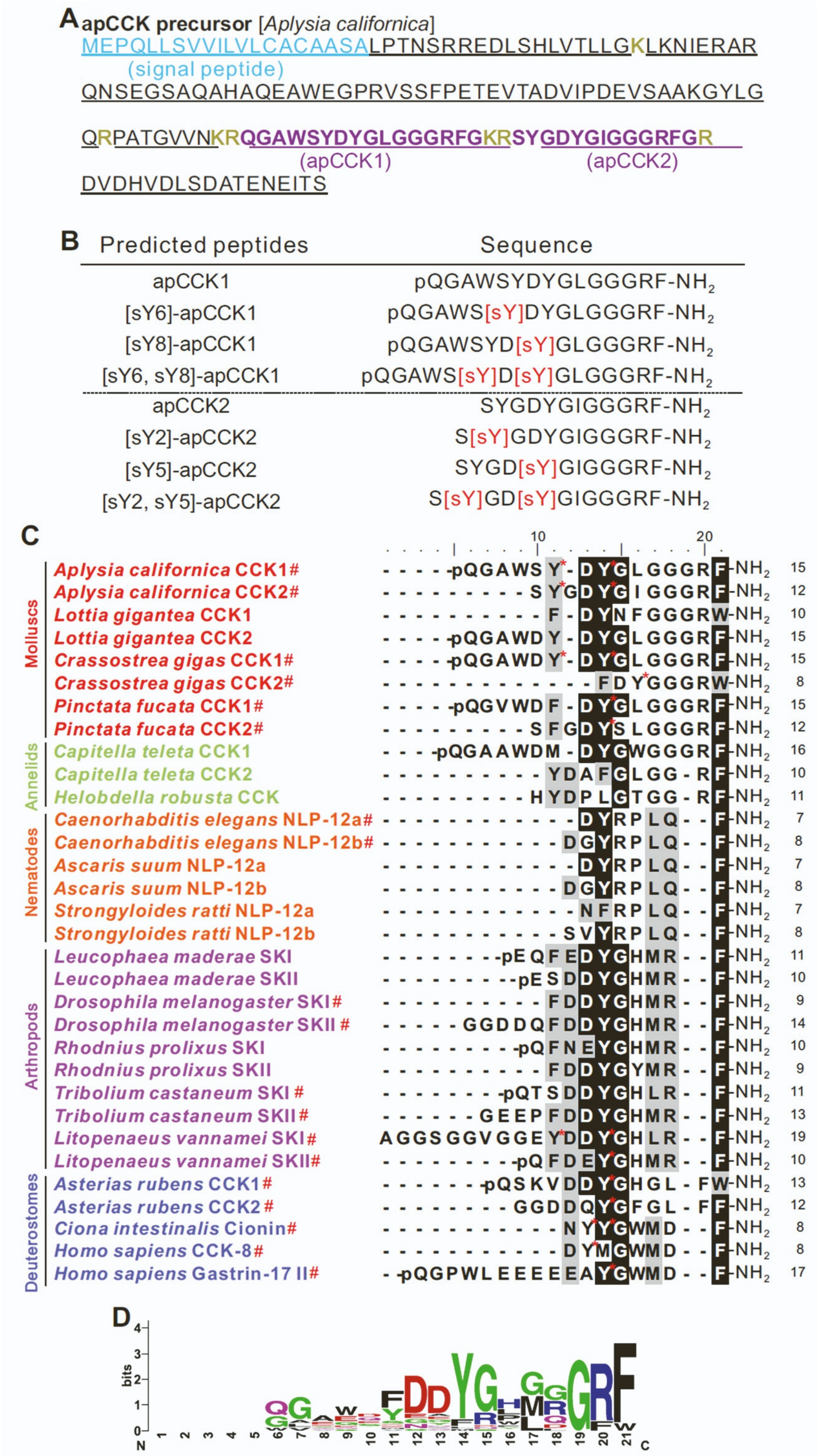

Figure S4: ***Aplysia*** **CCK precursor and comparison of CCKs from protostomes and deuterostomes. Related to Figure 1. (A)** The complete protein sequence of *Aplysia* CCK precursor illustrating the signal peptide and two predicted peptides: apCCK1 and apCCK2 (G at the C-terminus is typically amidated as the final product). K, R, KR (in light green) are potential basic cleavage sites. **(B)** The predicted apCCK peptides generated from *Aplysia* CCK precursor by NeuroPred website. **(C)**

Comparison of selected CCK peptides from protostomes and deuterostomes using BioEdit (ClustalW Multiple Alignment-Graphic view). # (to the right of peptide name) indicates that the sequence has been verified or studied before. * indicates the modification of the amino acid tryrosine with sulfation has been identified. pQ indicates pyro-glutamic acid. The first modified amino acid (pyro-glutamic acid) in some CCK sequences (*Lottia gigantea* CCK2, *Crassostrea gigas* CCK1, *Capitella teleta* CCK1, *Litopenaeus vannamei* SKII, *Homo sapiens* Gastrin-17 II) were indicated as pE in the paper of Schwartz et al., 2018. **(D)** A frequency plot for CCK sequences using Weblogo v2.8.2 (http://weblogo.berkeley.edu/logo.cgi). C-terminal RF-NH_2 and Y in the middle are highly conserved across species. See Dataset S4 for the source of the sequences.

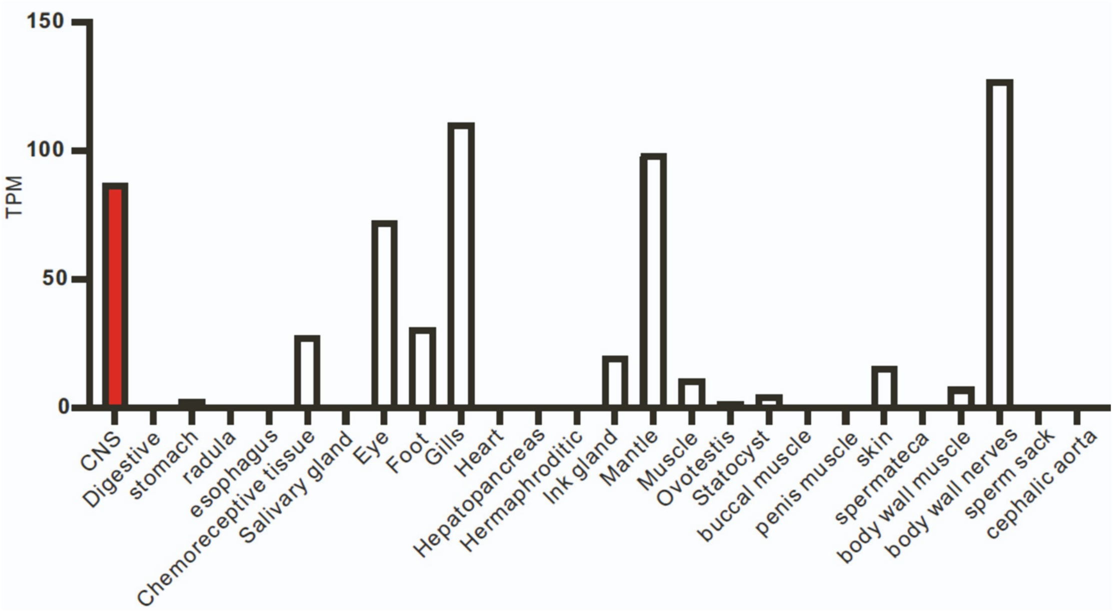

Figure S5: Relative expression levels of *Aplysia* CCK precursor from RNA profiling data. Related to Figure 1. Quantification of the expression of *apCCK precursor* (NCBI accession number: XM_005096206.2, now updated as XM_005096206.3) from RNA profiling data. The RNA profiling data was derived from NCBI website (GEO accession number: GSE79231). TPM: transcript per million.

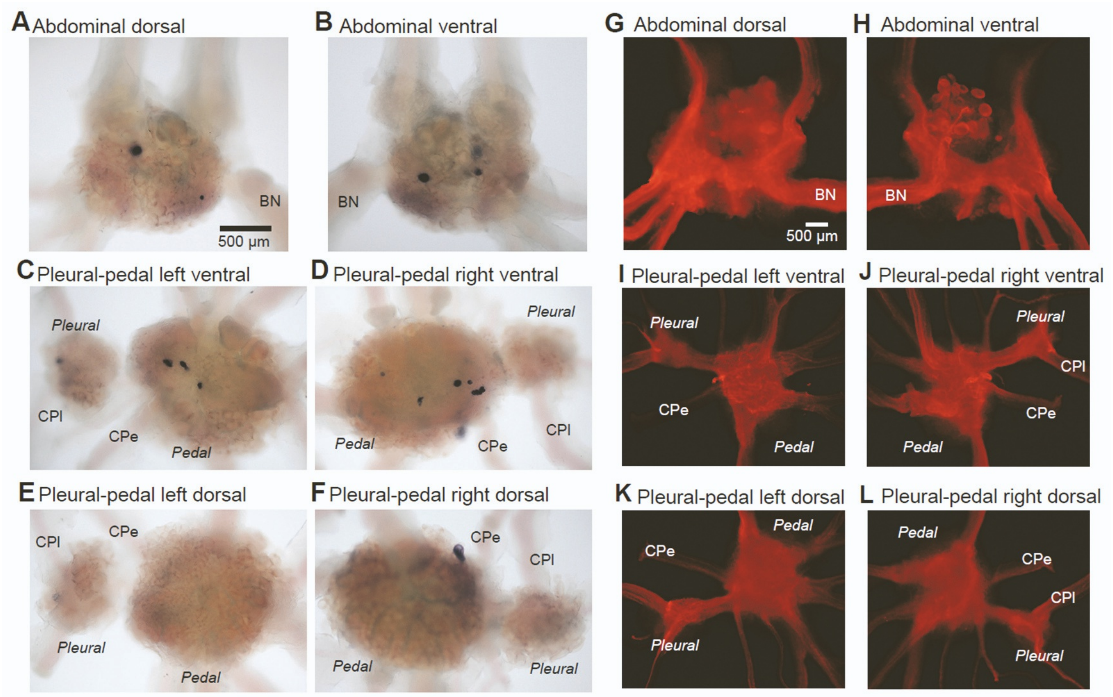

Figure S6: Distribution of apCCK-positive neurons in the abdominal and pleural-pedal ganglia (whole mounts) by in situ hybridization and immunohistochemistry. Related to Figure 1. (A-F) In situ hybridization of abdominal and pleural-pedal ganglia by utilizing the full length of apCCK precursor gene as a probe. **(A)** The dorsal surface of abdominal ganglia. **(B)** The ventral surface of abdominal ganglia. **(C)** Left ventral pleural-pedal ganglia. **(D)** Right ventral pleural-pedal ganglia. **(E)** Left dorsal pleural-pedal ganglia. **(F)** Right dorsal pleural-pedal ganglia. It is noted that there are few positive neurons in the dorsal and ventral surface of abdominal ganglia and several positive neurons are located on the ventral surface of pleural-pedal ganglia. Scale bars for (A-F), 500 µm in (A). **(G-L)** The apCCK immunohistochemistry of abdominal and pleural-pedal ganglia. **(G)** The dorsal surface of abdominal ganglia. **(H)** The ventral surface of abdominal ganglia. **(I)** Left ventral pleural-pedal ganglia. **(J)** Right ventral pleural-pedal ganglia. **(K)** Left dorsal pleural-pedal ganglia. **(L)** Right dorsal pleural-pedal ganglia. It is noted that the pattern of staining in immunohistochemistry is similar with that by *in situ* hybridization. Scale bars for (G-L), 500 µm in (G). Abbreviations are as follows. BN, branchial nerve; CPe, cerebral-pedal connective nerve; CPl, cerebral-pleural connective nerve.

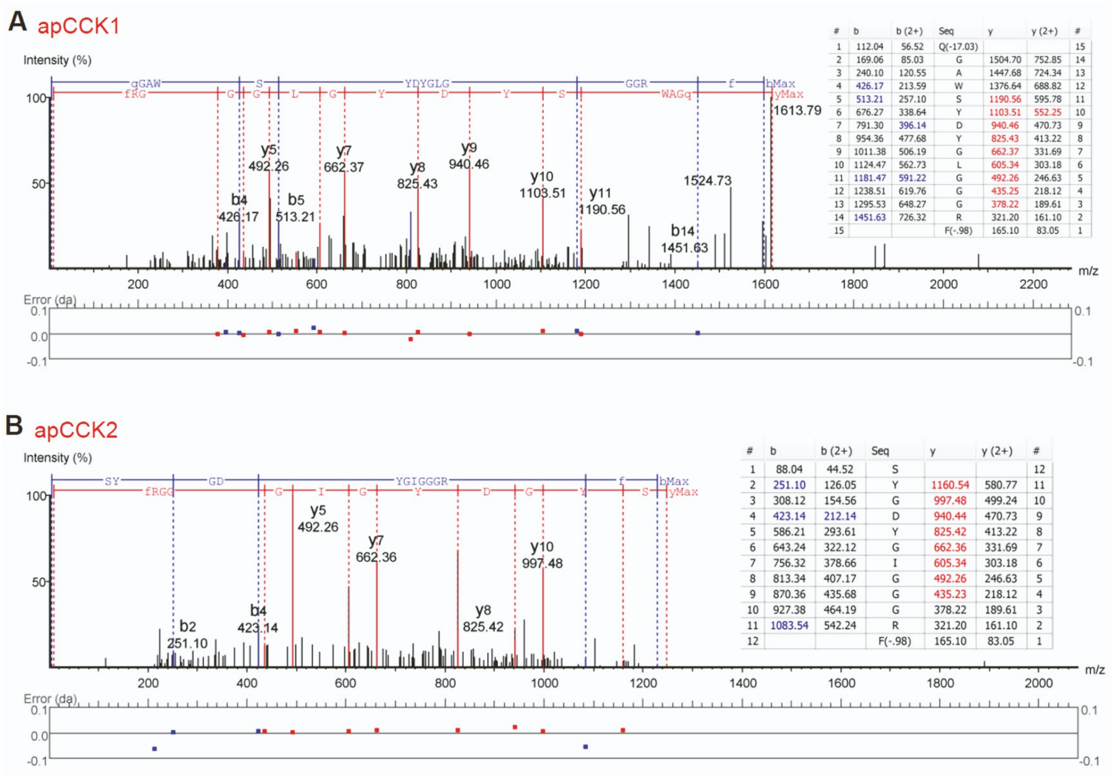

#	b	b (2+)	Seq	y	y (2+)	#
1	112.04	56.52	Q(-17.03)			15
2	169.06	85.03	G	1504.70	752.85	14
3	240.10	120.55	A	1447.68	724.34	13
4	426.17	213.59	W	1376.64	688.82	12
5	513.21	257.10	S	1190.56	595.78	11
6	676.27	338.64	Y	1103.51	552.25	10
7	791.30	396.14	D	940.46	470.73	9
8	954.36	477.68	Y	825.43	413.22	8
9	1011.38	506.19	G	662.37	331.69	7
10	1124.47	562.73	L	605.34	303.18	6
11	1181.47	591.22	G	492.26	246.63	5
12	1238.51	619.76	G	435.25	218.12	4
13	1295.53	648.27	G	378.22	189.61	3
14	1451.63	726.32	R	321.20	161.10	2
15			F(-.98)	165.10	83.05	1

#	b	b (2+)	Seq	y	y (2+)	#
1	88.04	44.52	S			12
2	251.10	126.05	Y	1160.54	580.77	11
3	308.12	154.56	G	997.48	499.24	10
4	423.14	212.14	D	940.44	470.73	9
5	586.21	293.61	Y	825.42	413.22	8
6	643.24	322.12	G	662.36	331.69	7
7	756.32	378.66	I	605.34	303.18	6
8	813.34	407.17	G	492.26	246.63	5
9	870.36	435.68	G	435.23	218.12	4
10	927.38	464.19	G	378.22	189.61	3
11	1083.54	542.24	R	321.20	161.10	2
12			F(-.98)	165.10	83.05	1

Figure S7: Sequence conformation of the *Aplysia* CCK peptides as detected by LC-MS in central ganglia. Related to Figure 1. (A) apCCK1: Q(-17.03)GAWSYDYGLGGGRF(-0.98). **(B)** apCCK2: SYGDYGIGGGRF(-0.98). Q(-17.03) is equivalent to [p-]Q; F(-0.98) is equivalent to F-NH_2.

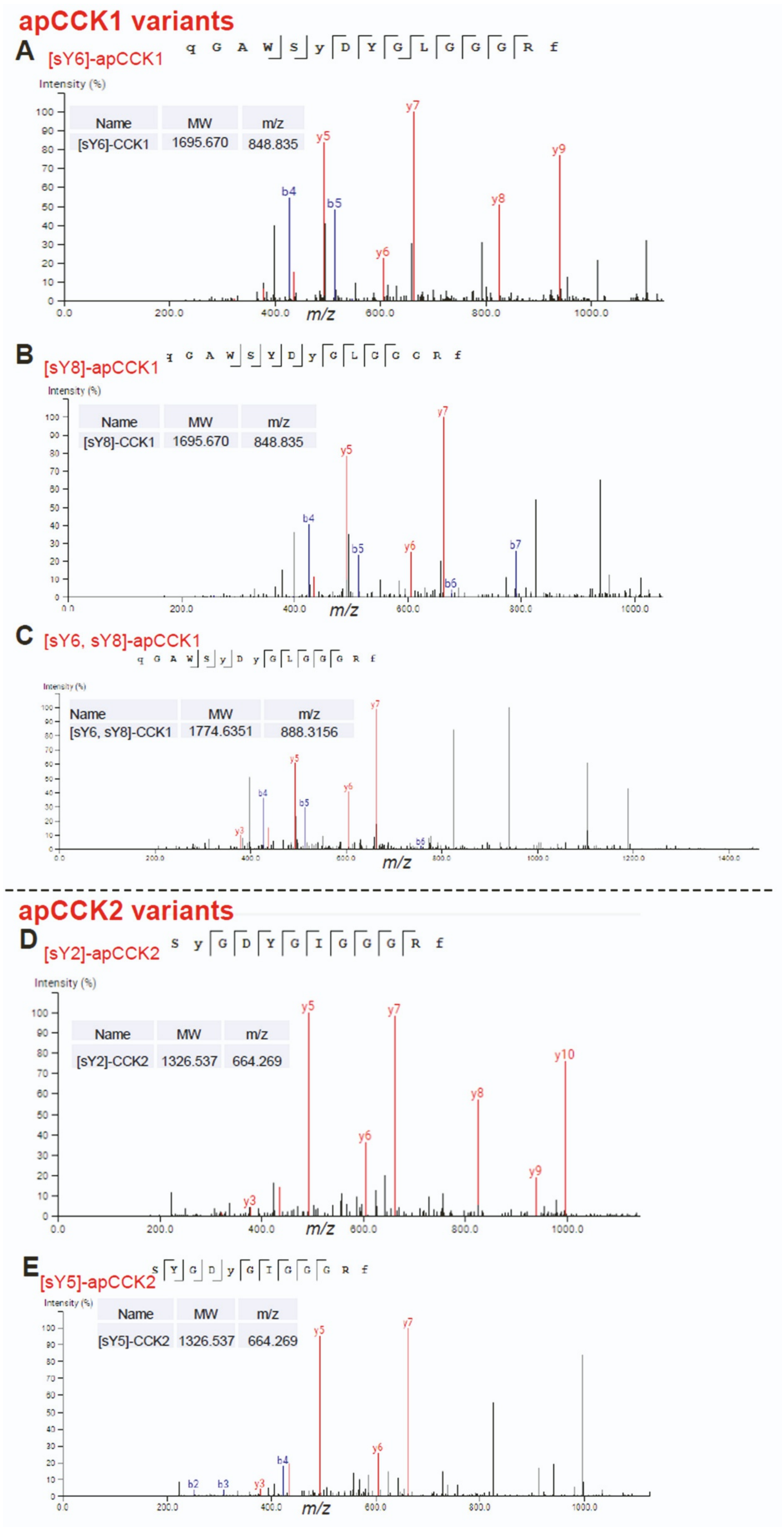

Figure S8: Sequence verification and PTM localization on detected apCCK peptides by tandem mass spectrometry. Related to Figure 1. Ion series are color coded: b-ions are blue and y-ions are red; vertical lines represent confirmed residues**.** **(A)** [sY6]-apCCK1: qGAWSyDYGLGGGRf. **(B)** [sY8]-apCCK1: qGAWSYDyGLGGGRf. **(C)** [sY6, sY8]-apCCK1: qGAWSyDyGLGGGRf. **(D)**

[sY2]-apCCK2: SyGDYGIGGGRf. **(E)** [sY5]-apCCK2: SYGDyGIGGGRf. q: pQ. f: F-NH_2.

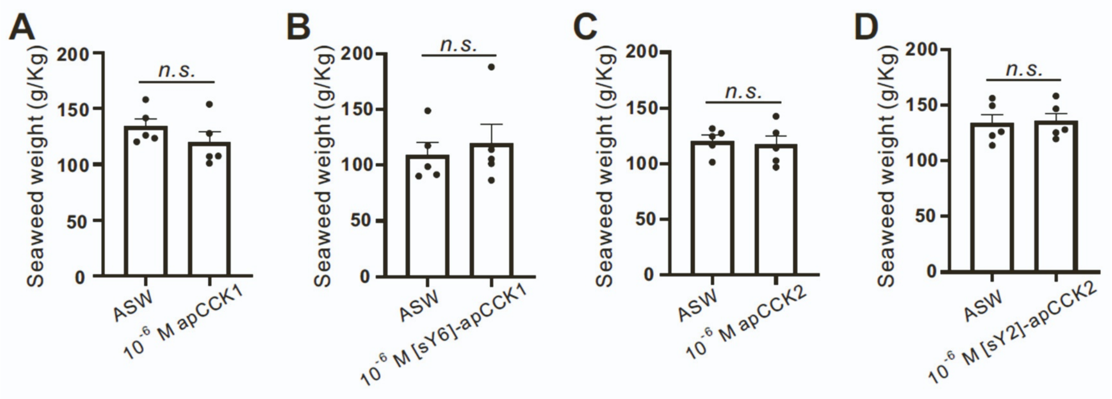

Figure S9: apCCK peptides without sulfation modification or mono-sulfated modification located toward the N-terminus had no significant effects on feeding behavior. Related to Figure 2. (A) apCCK1. Paired *t* test, $t_4 = 2.235$, $P = 0.0891$. **(B)** [sY6]-apCCK1. Paired *t* test, $t_4 = 1.036$, $P = 0.3588$. (**C**) apCCK2. Paired *t* test, $t_4 = 0.4378$, $P = 0.6842$. **(D)** [sY2]-apCCK2. Paired *t* test, $t_4 = 0.4179$, $P = 0.6975$. n = 5 for all cases. Error bar: SEM. *n. s.*: $P > 0.05$

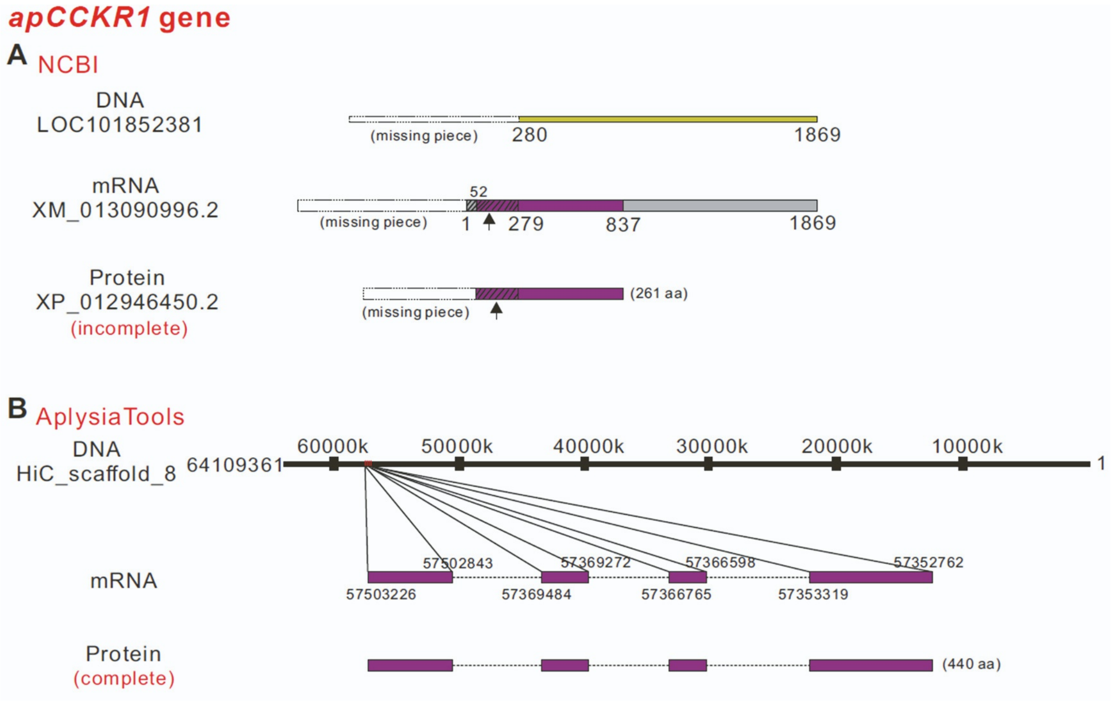

Figure S10: Gene expression mapping of *apCCKR1*. Related to Figure 3. (A) The gene (LOC101852381) corresponds to one mRNA XM_013090996.2, with two introns (colored in grey), which produces a protein XP_012946450.2, named *Aplysia* californica cholecystokinin receptor type A. The protein exhibits similarity to CCK receptors found in other mollusks. However, it is incomplete and lacks the N-terminal sequence (537 bp), as indicated by the dashed box in the figure. The arrows indicate that 279 bases not found in genome assembly. **(B)** DNA HiC_scaffold_8 (Note that the nucleotide number on top starts from the right) from *Aplysia* gene nucleotide databases (the AplysiaTools) expresses the similar but shorter mRNA than that in (A). However, the protein generated from this mRNA is longer than that in (A). This protein is complete and consists of seven transmembrane domains.

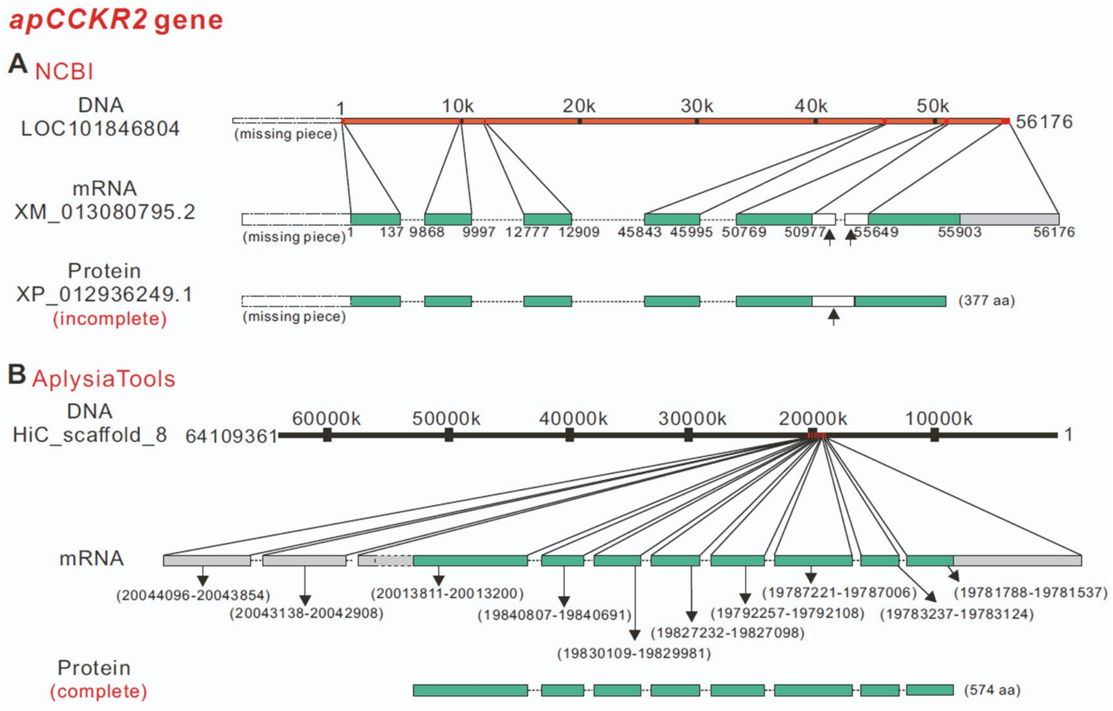

Figure S11: Gene expression mapping of *Aplysia CCKR2*. Related to Figure 3. (A) The gene (LOC101846804) corresponds to one mRNA XM_013080795.2, with an intron between 55903 to 56176 bp, which produces a protein XP_012936249.1 (the arrows indicate 117 bases that could not be found in genome assembly), named *Aplysia* californica cholecystokinin receptor. The protein is similar to CCK receptors from other mollusks. However, it is incomplete and lacks some sequences (591 bp) in the N-terminus, as indicated by the dashed box in the figure. **(B)** DNA HiC_scaffold_8 (Note that the nucleotide number on top starts from the right) from *Aplysia* gene nucleotide databases (the AplysiaTools) expresses a similar but longer mRNA than that in (A). This protein is complete and consists of seven transmembrane domains.

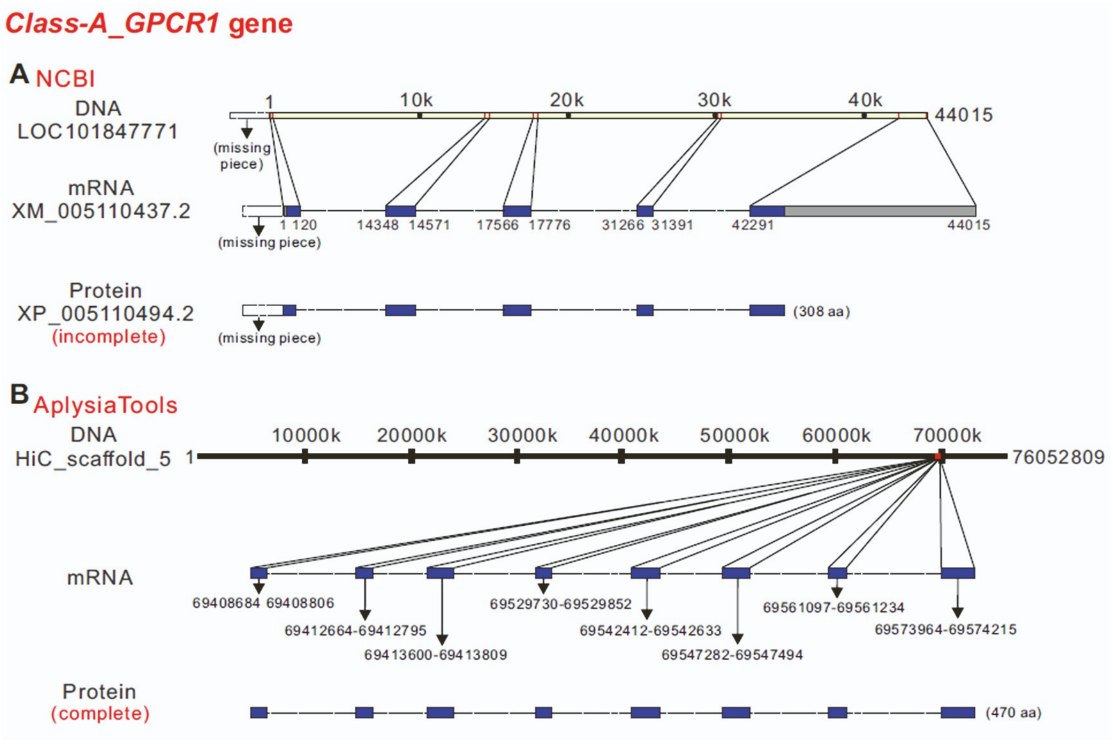

Figure S12: Gene expression mapping of *Aplysia class-A_GPCR1*. Related to Figure 3. (A) The gene LOC101847771 corresponds to one mRNA XM_005110437.2, with two introns (colored in grey), which produces a protein XP_005110494.2, named *Aplysia* californica cholecystokinin receptor type A-like. This protein is incomplete and lacks some sequence (486 bp) in N-terminus (labeled in dashed box). **(B)** DNA HiC_scaffold_5 from *Aplysia* gene nucleotide databases (the AplysiaTools) expresses a similar but shorter mRNA than that in (A). However, the protein generated from this mRNA is longer than that in (A). This protein is complete and consists of seven transmembrane domains.

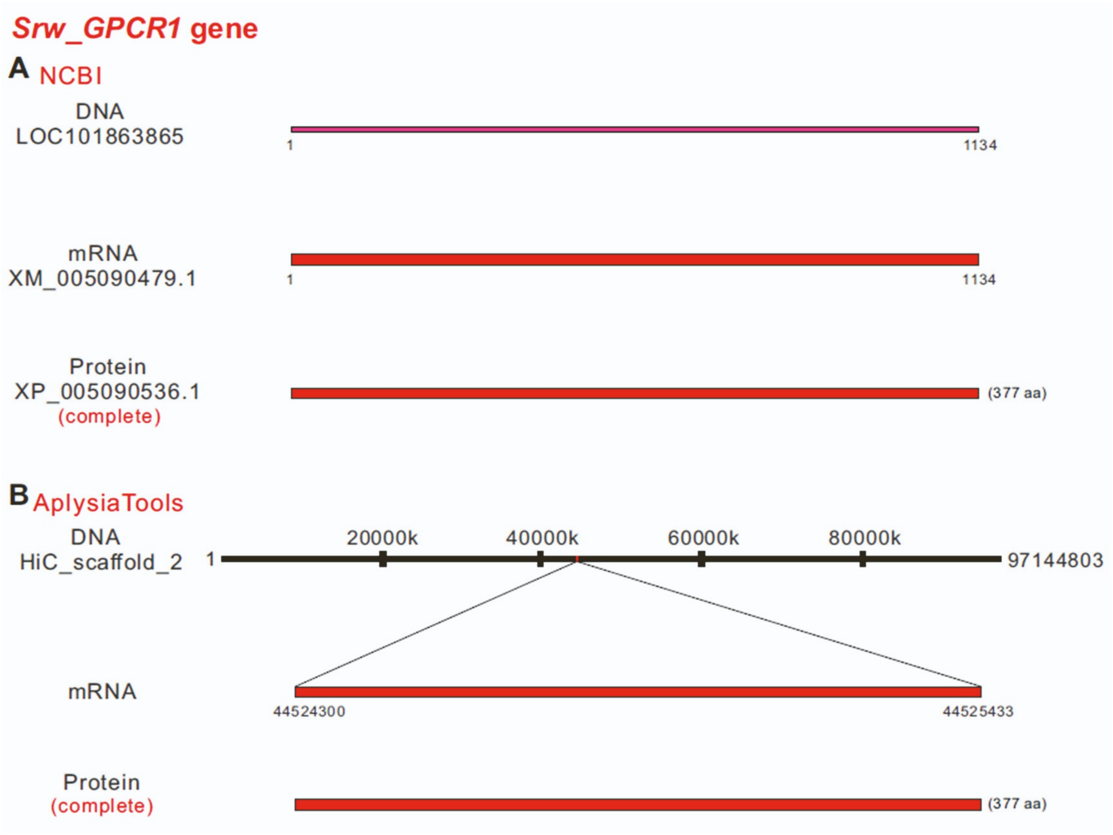

Figure S13: Gene expression mapping of *Aplysia Srw_GPCR1*. Related to Figure 3. (A) The gene LOC101863865 corresponds to one mRNA XM_005090479.1, with no intron, which produces a protein XP_005090536.1, named *Aplysia* californica cholecystokinin receptor type A-like. The sequence is complete. **(B)** DNA HiC_scaffold_2 from *Aplysia* gene nucleotide databases (the AplysiaTools) expresses the identical mRNA as in (A), and the protein generated from this mRNA is the same as that in (A).

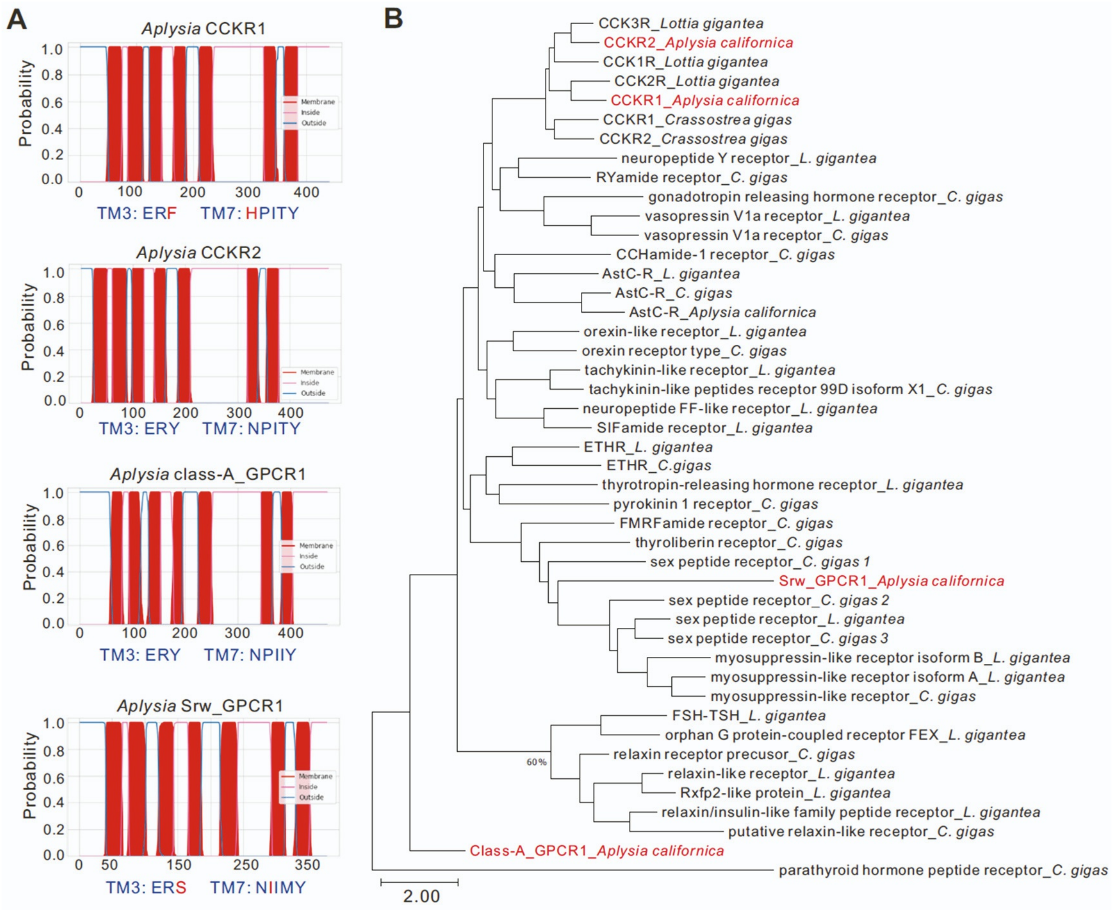

Figure S14: Bioinformatics of putative *Aplysia* CCK receptors. Related to Figure 3. (A) Prediction of seven transmembrane domains of putative receptors: CCKR1, CCKR2, Class-A_GPCR1, and Srw_GPCR1 using TMHMM. Conserved motifs in transmembrane domain 3 (TM3, D/ERY) and TM7 (NPXXY) are shown. The amino acids different from the conserved motifs are shown in red. **(B)** A phylogenetic tree of four *Aplysia* proteins, CCKR1, CCKR2, Class-A_GPCR1, and Srw_GPCR1 with *A. californica*, *L. gigantea* and *C. gigas* Class A GPCR sequences from Jiang (see the Results and Dataset S5) using MEGA X. A Class-B GPCR, parathyroid hormone peptide receptor_*C. gigas*, was used as an outgroup. The tree suggests that *Aplysia* CCKR1 and CCKR2 are likely *Aplysia* CCK receptors, whereas *Aplysia* Class-A_GPCR1 and Srw_GPCR1 are not. The tree is drawn to scale, with branch lengths measured in the number of substitutions per site. Number at the nodes are bootstrap values as percentage. Only bootstrap values greater than 50 are shown.

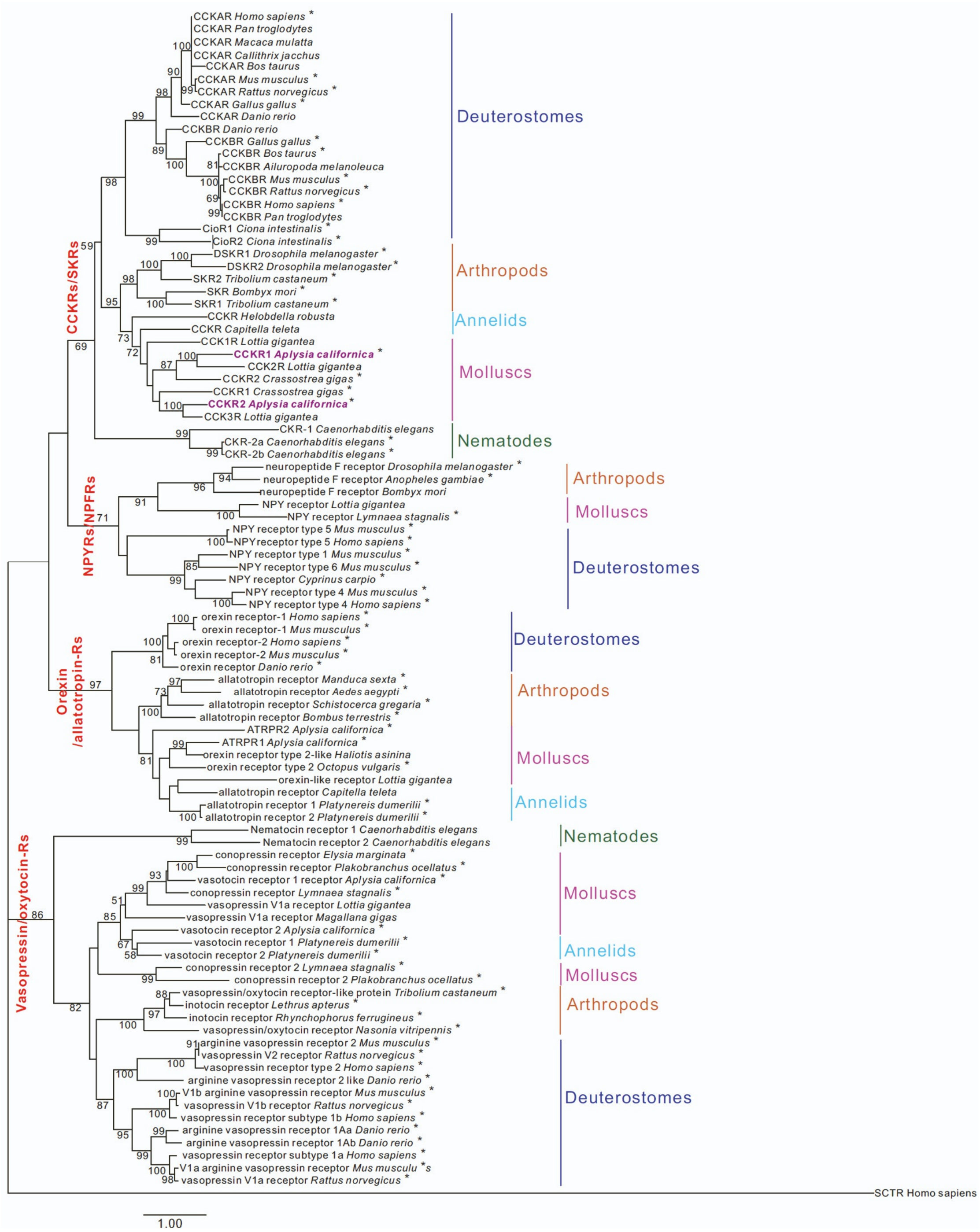

Figure S15: A phylogenetic tree of CCK receptors in *Aplysia* with predicted or verified CCK/SK receptors, NPY/NPF receptors, orexin/allatotropin receptors, and vasopressin/oxytocin receptors in protostomes and deuterostomes. Related to Figure 3. The tree was generated using MEGA X with 1000 replicates (See the bioinformatic section in Methods for more detailed and Dataset S6 for information on the sequences). This phylogenetic tree indicated that all CCKRs/SKRs including molluscan ones, form one clad. CCK/SK receptors are closely related to NPY/NPF receptor, which may result from their ligands generally share the common C-terminal residues of "RF". Orexin/allatotropin-Rs and vasopressin/oxytocin-Rs are more distantly related. Within CCKR/SKRs, *Aplysia* CCKRs were more closely related to

the CCKR sequences in molluscan *Lottia gigantea*. * indicates that the receptor has been verified or studied. “SCTR Homo sapiens” is a Class-B GPCR used as an outgroup. Numbers at the nodes are bootstrap values as percentage. Only bootstrap values greater than 50 are shown.

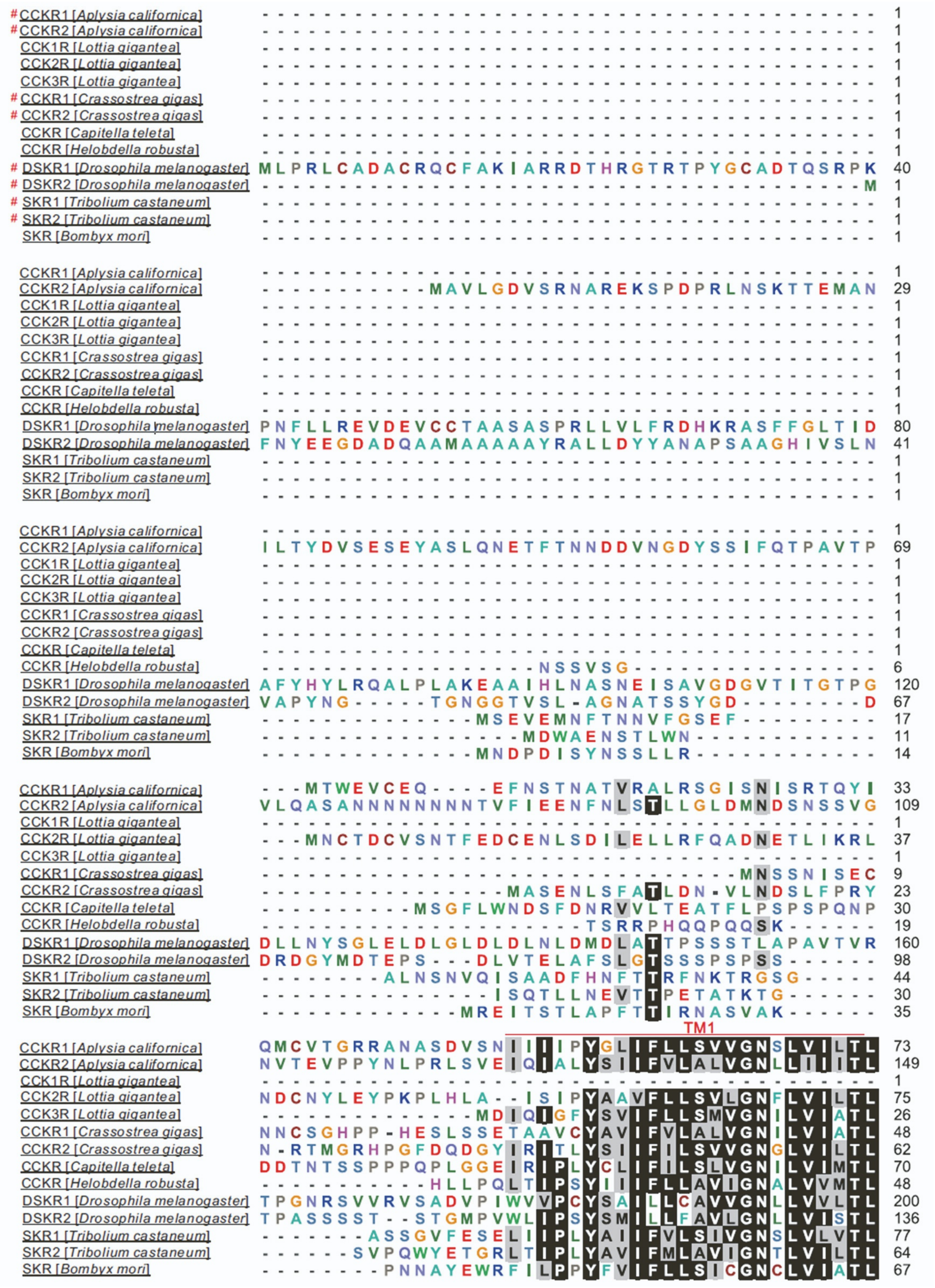

#CCKR1 [Aplysia californica]
#CCKR2 [Aplysia californica]
CCK1R [Lottia gigantea]
CCK2R [Lottia gigantea]
CCK3R [Lottia gigantea]
#CCKR1 [Crassostrea gigas]
#CCKR2 [Crassostrea gigas]
CCKR [Capitella teleta]
CCKR [Helobdella robusta]
DSKR1 [Drosophila melanogaster]
DSKR2 [Drosophila melanogaster]
SKR1 [Tribolium castaneum]
SKR2 [Tribolium castaneum]
SKR [Bombyx mori]
TM1

(page 1)

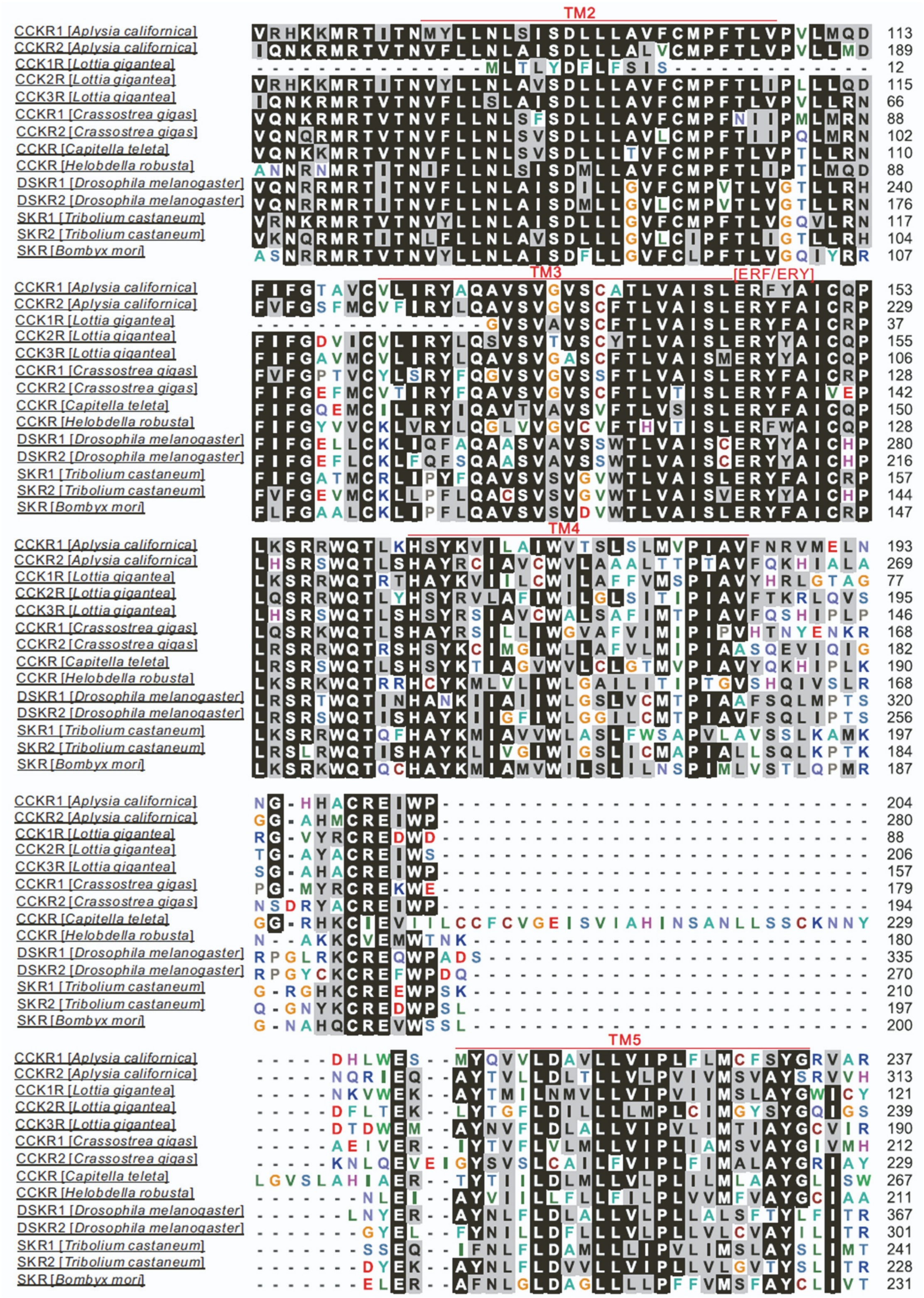
TM2
TM3
[ERF/ERY]
TM4
TM5
CCKR1 [Aplysia californica]
CCKR2 [Aplysia californica]
CCK1R [Lottia gigantea]
CCK2R [Lottia gigantea]
CCK3R [Lottia gigantea]
CCKR1 [Crassostrea gigas]
CCKR2 [Crassostrea gigas]
CCKR [Capitella teleta]
CCKR [Helobdella robusta]
DSKR1 [Drosophila melanogaster]
DSKR2 [Drosophila melanogaster]
SKR1 [Tribolium castaneum]
SKR2 [Tribolium castaneum]
SKR [Bombyx mori]

(page 2)

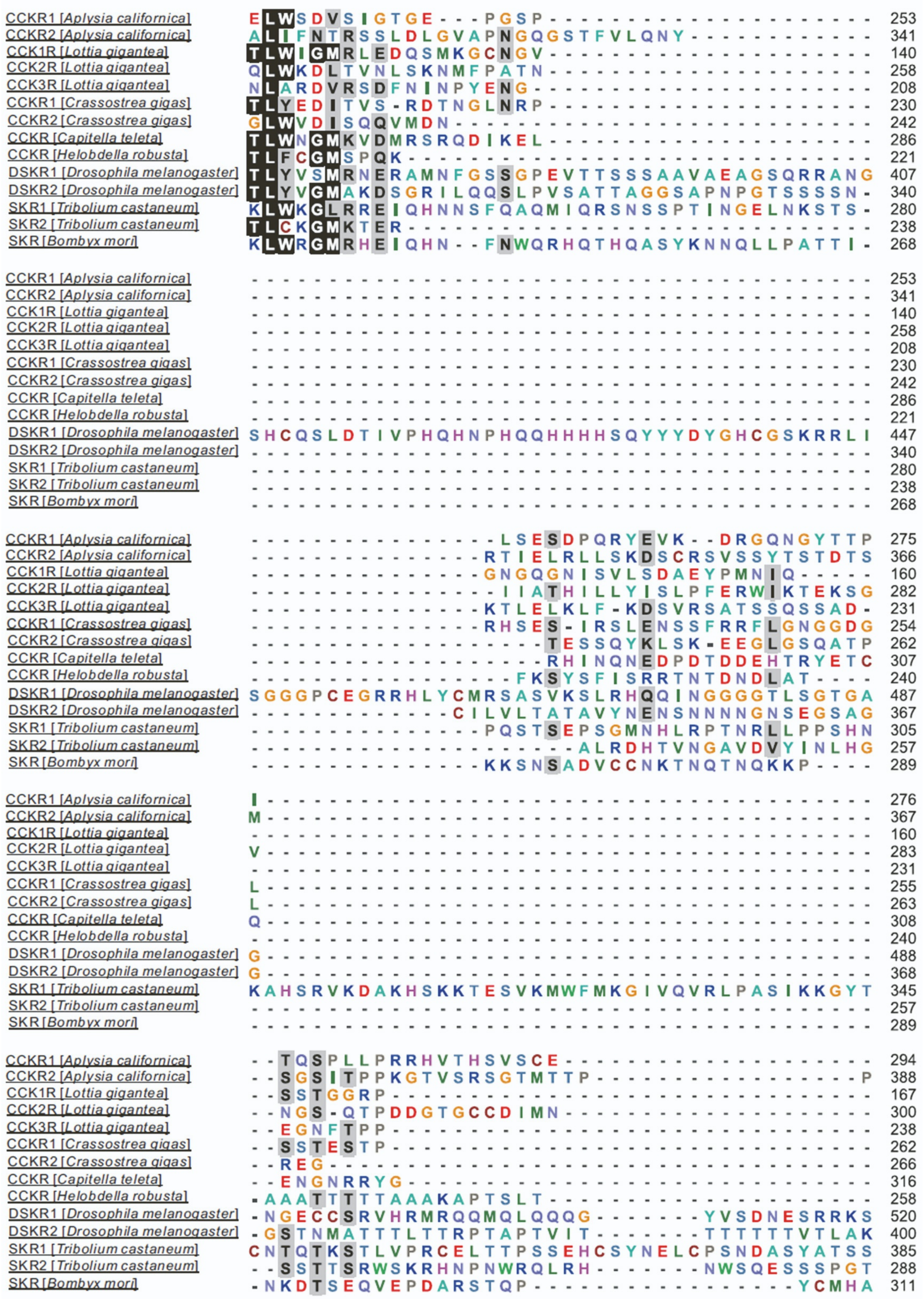
CCKR1 [Aplysia californica]
CCKR2 [Aplysia californica]
CCK1R [Lottia gigantea]
CCK2R [Lottia gigantea]
CCK3R [Lottia gigantea]
CCKR1 [Crassostrea gigas]
CCKR2 [Crassostrea gigas]
CCKR [Capitella teleta]
CCKR [Helobdella robusta]
DSKR1 [Drosophila melanogaster]
DSKR2 [Drosophila melanogaster]
SKR1 [Tribolium castaneum]
SKR2 [Tribolium castaneum]
SKR [Bombyx mori]

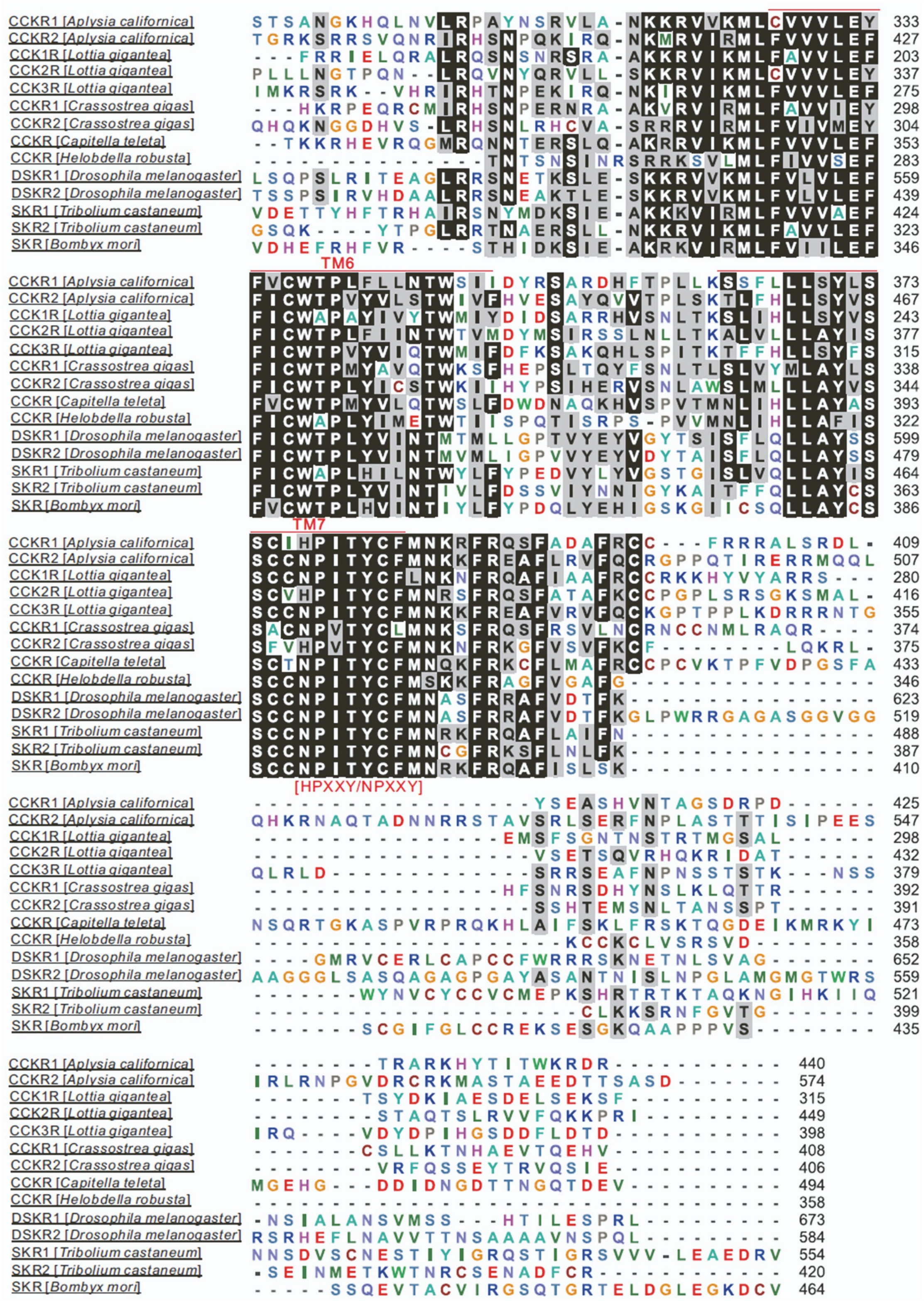

(page 4)

Figure S16: The alignment of CCK receptors from several protostomes including mollusks (*Aplysia californica, Lottia gigantea, Crassostrea gigas*), annelids (*Capitella teleta, Helobdella robusta*), and arthropods (*Drosophila melanogaster, Tribolium castaneum, Bombyx mori*) using BioEdit (ClustalW Multiple

Alignment-Graphic view). Related to Figure 3. The red lines show the locations of the seven transmembrane domains. The locations of conserved motifs of TM3 (D/ERY) and TM7 in GPCR (NPXXY) are indicated in the figure. #: the CCK receptors have been identified.

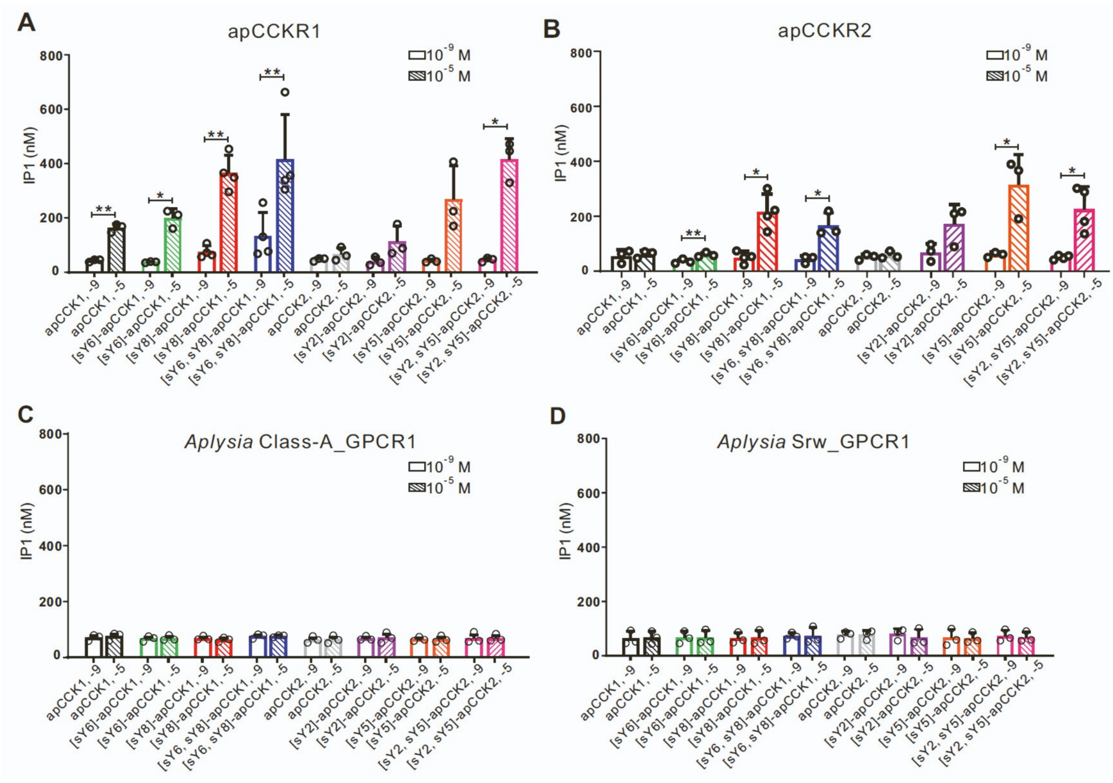

Figure S17: Screening of potential activation of peptide ligands on *Aplysia* putative CCK receptors using two concentrations by IP1 accumulation assay in CHO cells. Related to Figure 3. (A) Screening of potential activation of peptide ligands on putative receptor apCCKR1 using two concentrations: 10^{-9} M and 10^{-5} M. Paired *t* test, *, $P < 0.05$, **, $P < 0.01$, error bar: SEM. **(B)** Screening of potential activation of peptide ligands on putative receptor apCCKR2 using two concentrations: 10^{-9} M and 10^{-5} M. Paired *t* test, *, $P < 0.05$, **, $P < 0.01$, error bar: SEM. **(C)** Screening of potential activation of peptide ligands on putative receptor Class-A_GPCR1 using two concentrations: 10^{-9} M and 10^{-5} M. Error bar: SEM. **(D)** Screening of potential activation of peptide ligands on putative receptor Srw_GPCR1 using two concentrations: 10^{-9} M and 10^{-5} M. Error bar: SEM.

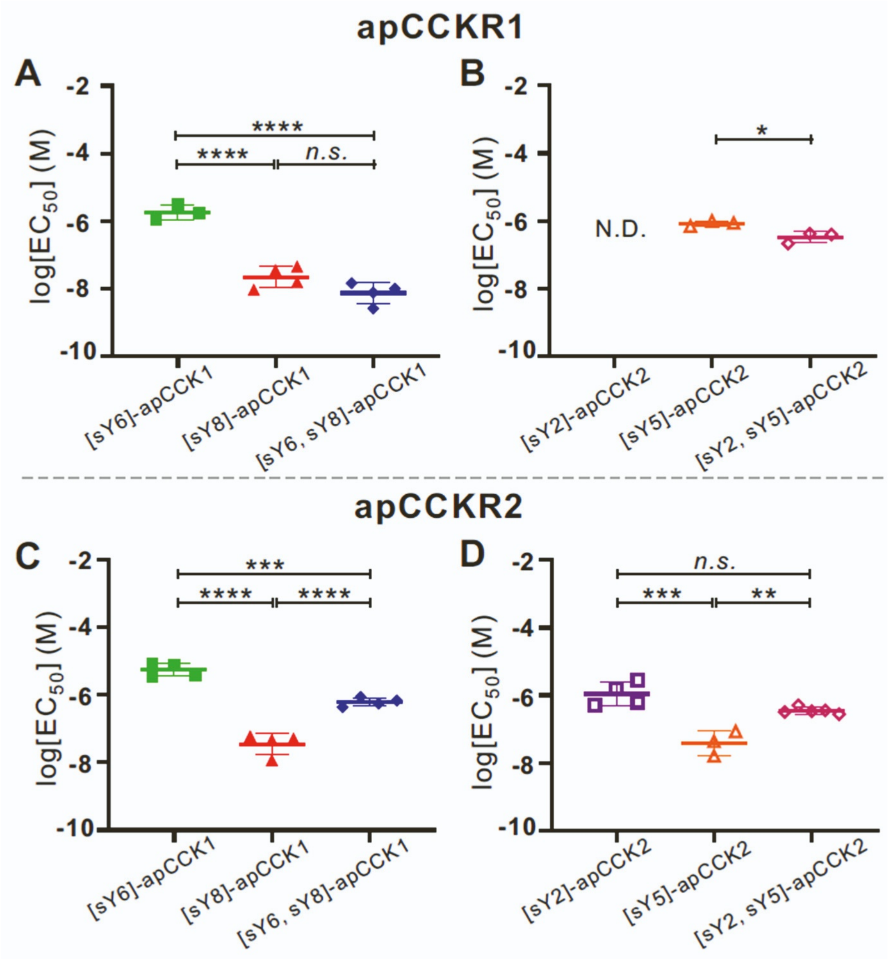

Figure S18: Most of mono-sulfated apCCKs located toward the C-terminus exhibited stronger activation of apCCKRs by IP1 accumulation assay in CHO cells. Related to Figure 3. (A) The peptides [sY8]-apCCK1 and [sY6, sY8]-apCCK1 exhibited stronger activities on apCCKR1 compared to the peptide [sY6]-apCCK1(one-way ANOVA, F (2, 8) = 58.94, $P < 0.0001$). **(B)** The peptide [sY2, sY5]-apCCK2 had stronger activities on apCCKR1 than the peptide [sY5]-apCCK2 (unpaired t test, $t_4 = 3.892$, $P = 0.0177$). **(C)** The peptide [sY8]-apCCK1 showed stronger activity on apCCKR2 than the peptides [sY6]-apCCK1 and [sY6, sY8]-apCCK1 (one-way ANOVA, F (2, 9) = 91.8, $P < 0.0001$). **(D)** The peptide [sY5]-apCCK2 showed stronger activity on apCCKR2 compared to the peptides [sY2]-apCCK2 and [sY2, sY5]-apCCK2 (one-way ANOVA, F (2, 9) = 25.19, $P = 0.0002$). Non-sulfated apCCK1 and apCCK2 weakly activated apCCKRs and were not included in this analysis. Tukey post hoc test: * $P < 0.05$; ** $P < 0.01$; *** $P < 0.001$; **** $P < 0.0001$; *n.s.*, $P > 0.05$. Error bar: SD. N.D.: not determined.

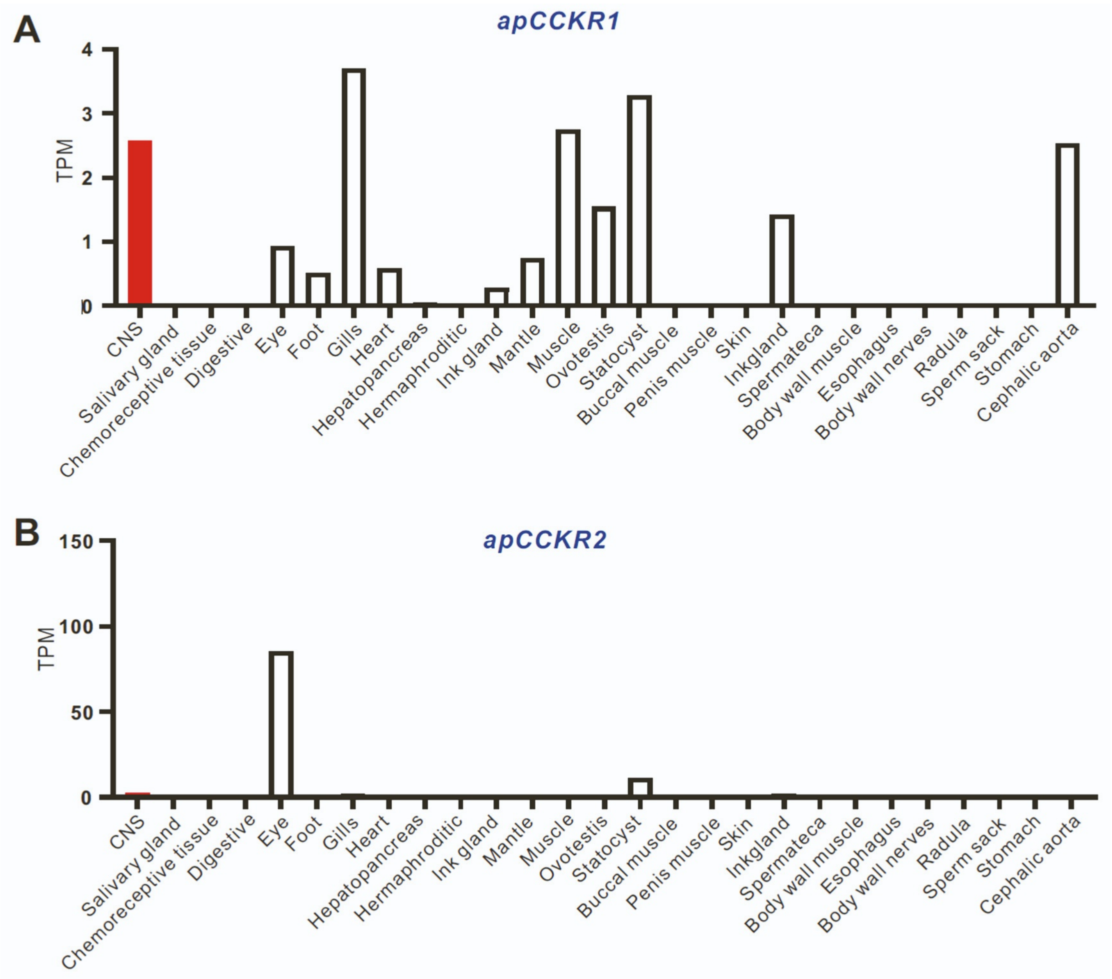

Figure S19: Relative expression levels of *apCCKR1* (NCBI accession number: XM_013090996.1, as indicated in GSE79231) and *apCCKR2* (NCBI accession number: XM_013080795.1, as indicated in GSE79231) from RNA profiling data. Related to Figure 3. The RNA profiling data was derived from NCBI website (GEO accession number: GSE79231). Note that XM_013090996.1 and XM_013080795.1 have been updated as XM_013090996.2 and XM_013080795.2 in NCBI, respectively. TPM: transcript per million.

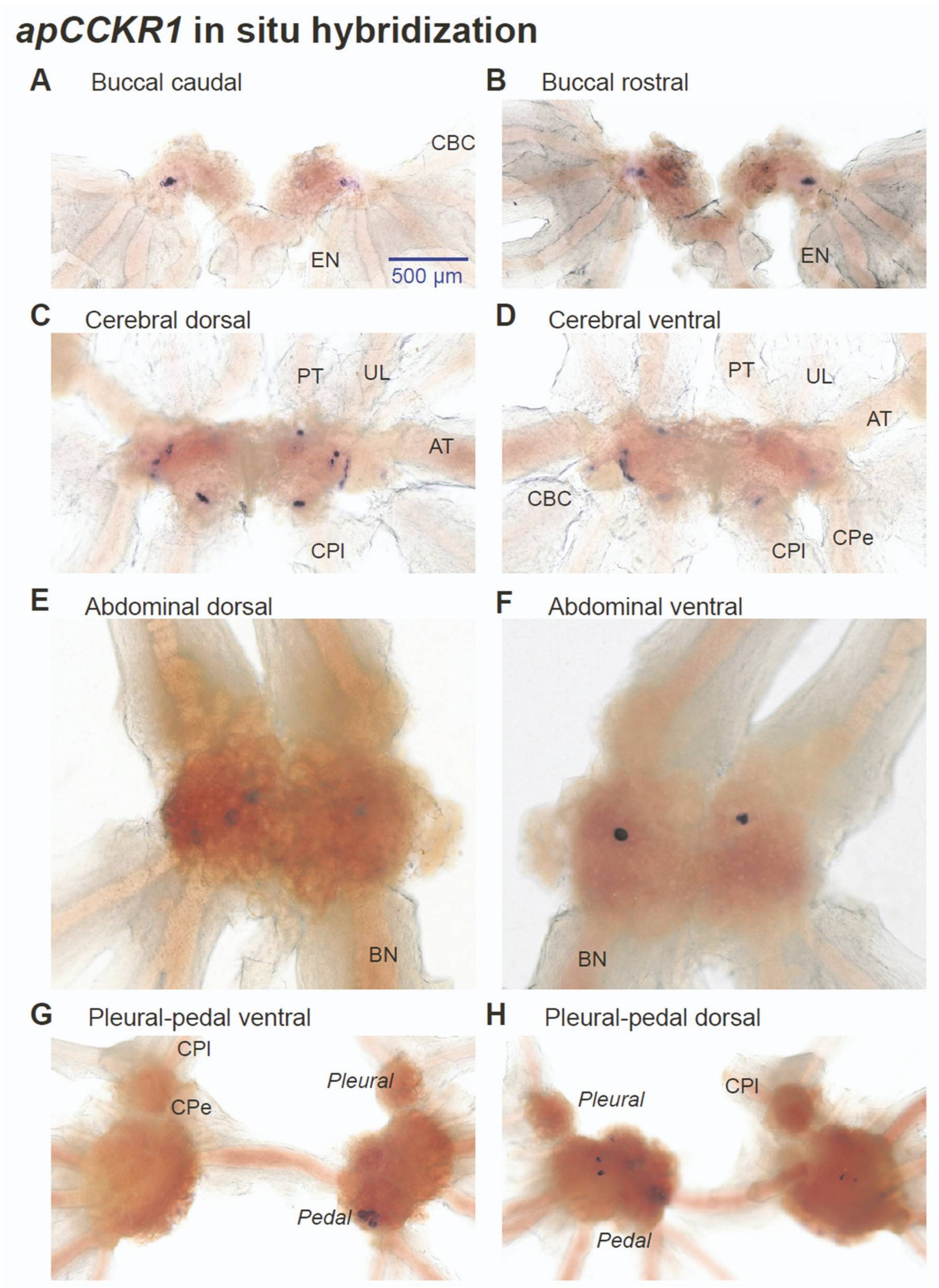

Figure S20: Distribution of *apCCKR1*-positive neurons in the buccal, cerebral, abdominal and pleural-pedal ganglia (whole mounts) by in situ hybridization. Related to Figure 3. (A, B) Caudal (A) and rostral (B) buccal ganglia. **(C, D)** Dorsal (C) and ventral (D) cerebral ganglia. **(E, F)** Dorsal (E) and ventral (F) abdominal ganglia. **(G, H)** Ventral (G) and dorsal (H) pleural-pedal ganglia. Scale bar, 500 µm in (A). Buccal abbreviations are as follows. EN, esophageal nerve; CBC, cerebral-buccal connective nerve. Cerebral abbreviations are as follows. UL, upper labial nerve; PT, posterior tentacular nerve; AT, anterior tentacular nerve; CPe, cerebral-pedal connective nerve; CPl, cerebral-pleural connective nerve. Abdominal abbreviations are as follows. BN, branchial nerve.

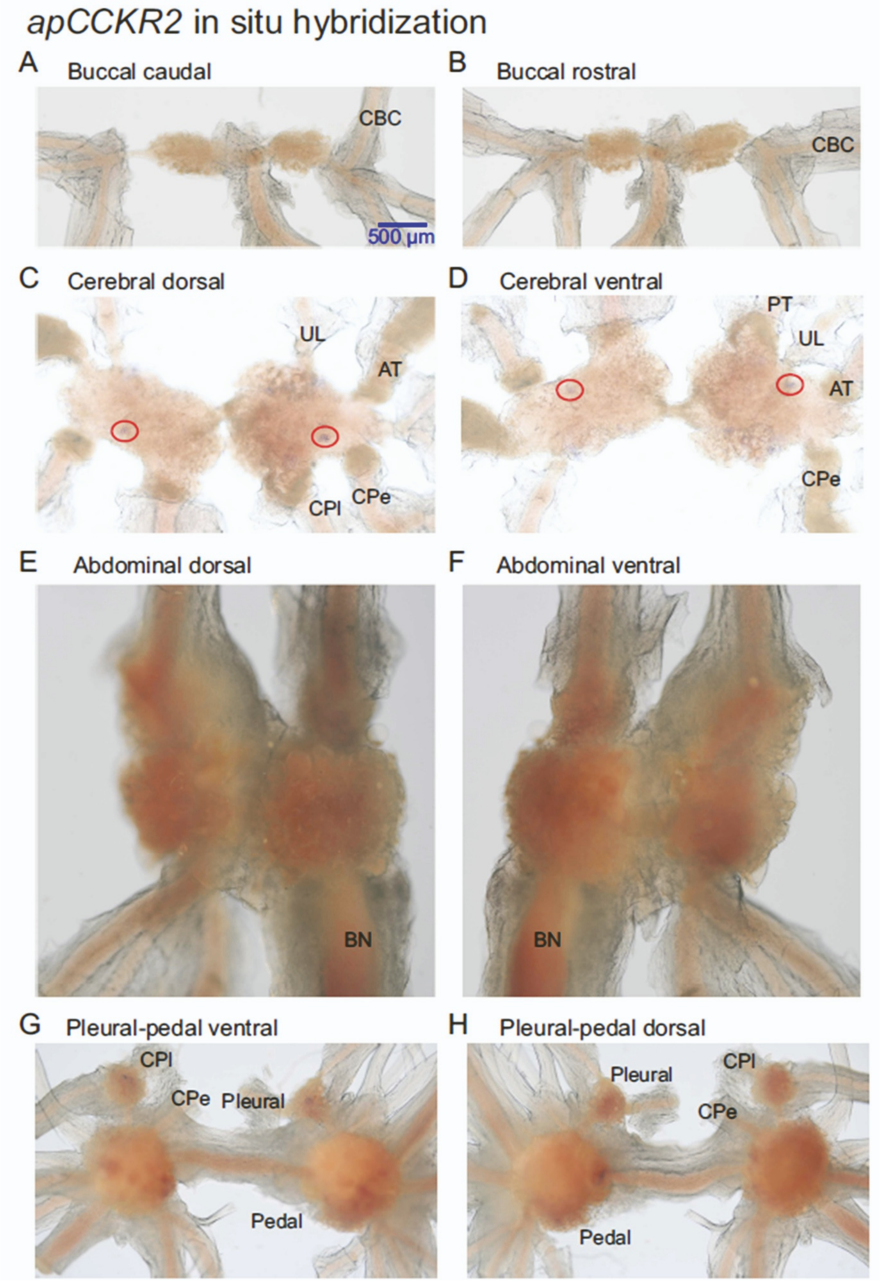

Figure S21: Distribution of *apCCKR2*-positive neurons in the buccal, cerebral, abdominal and pleural-pedal ganglia (whole mounts) by in situ hybridization. Related to Figure 3. (A, B) Caudal (A) and rostral (B) buccal ganglia. **(C, D)** Dorsal (C) and ventral (D) cerebral ganglia. **(E, F)** Dorsal (E) and ventral (F) abdominal ganglia. **(G, H)** Ventral (G) and dorsal (H) pleural-pedal ganglia. There appear to be two cells weakly stained with apCCKR2 on the dorsal and ventral surfaces of the cerebral ganglion (red circle). Scale bar, 500 µm in (A). Buccal abbreviations are as follows. CBC, cerebral-buccal connective nerve. Cerebral abbreviations are as follows. UL, upper labial nerve; PT, posterior tentacular nerve; AT, anterior tentacular nerve; CPe, cerebral-pedal connective nerve; CPl, cerebral-pleural connective nerve. Abdominal abbreviations are as follows. BN, branchial nerve.

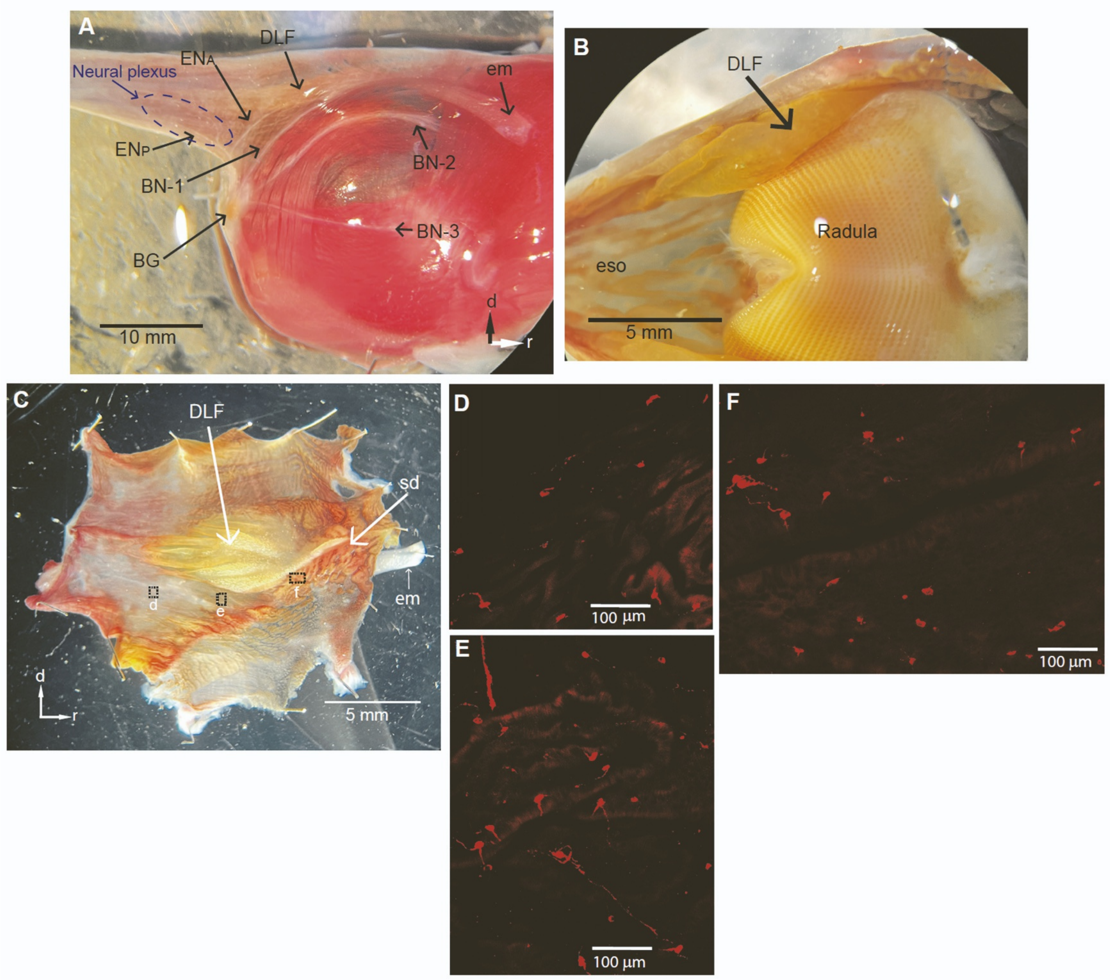

Figure S22: The anatomical location of dorsal longitudinal folds (DLF) and apCCK-positive neurons in the esophagus and pharynx by immunohistochemistry. Related to Figure 4. (A) An external photomicrograph of the posterior 2/3 of the buccal mass and anterior esophagus. **(B)** A view (in a different preparation) from the dorsal side where the esophagus (eso) and lining of the buccal mass has been cut along the midline, revealing the inner lining of cavities and the radula which has been pinned in the extreme retracted position. The DLF clearly frames the opening of the radula in this position. **(C)** The DLF from the same animal as the panel A, is shown in this stretched-out preparation of the lining of the esophagus and posterior buccal cavity (pharynx), with the endothelial side facing the camera. The positions and sizes of panels D, E, and F are indicated by the labeled dashed boxes (d, e, and f) in panel C. **(D-F)** apCCK-IR neurons are shown in the anterior esophagus **(D)**, and the pharynx **(E, F)**. In addition to apCCK-IR neurons, there is some background staining of the endothelium and in panel F, the denticles characteristic of much of the DLF. In all the panels, but particularly panel E, the apCCK-IR cells often are seen to have a process that extends through the endothelial layer. The densest population of apCCK-IR neurons is located ventral to the DLF, and

extends from the anterior (rostral) part of the esophagus (box d) to between box f and the salivary duct (sd). There is also a moderately dense concentration of apCCK-IR cells in a narrow strip located just dorsal to the DLF as well as scattered cells in other areas except the DLF. BG, buccal ganglion; BN-1, buccal nerve 1; BN-2, buccal nerve 2; BN-3 buccal nerve-3; d, dorsal; EN_A, anterior branch esophageal nerve; EN_P, posterior branch esophageal nerve; em, extrinsic muscle.

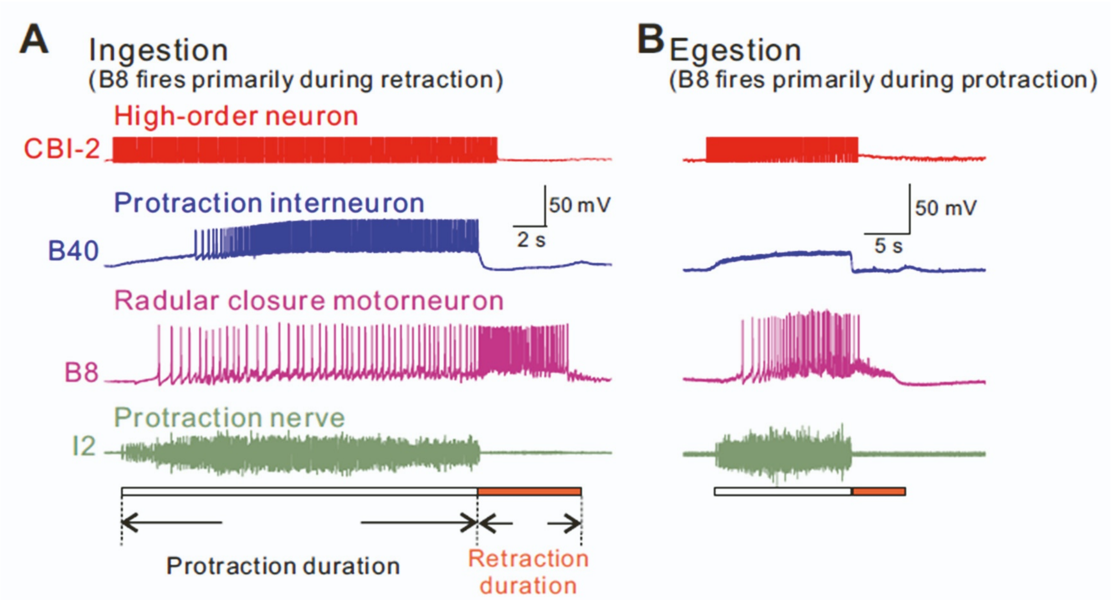

Figure S23: **Representative examples of ingestive programs and egestive programs elicited by the high-order neuron CBI-2 illustrating how these two types of motor programs are distinguished. Related to Figure 5.** In ingestive programs **(A)**, the radular closure motoneuron B8 fires primarily during retraction to pull food in, while in egestive programs **(B)**, B8 fires primarily during protraction to push food out.

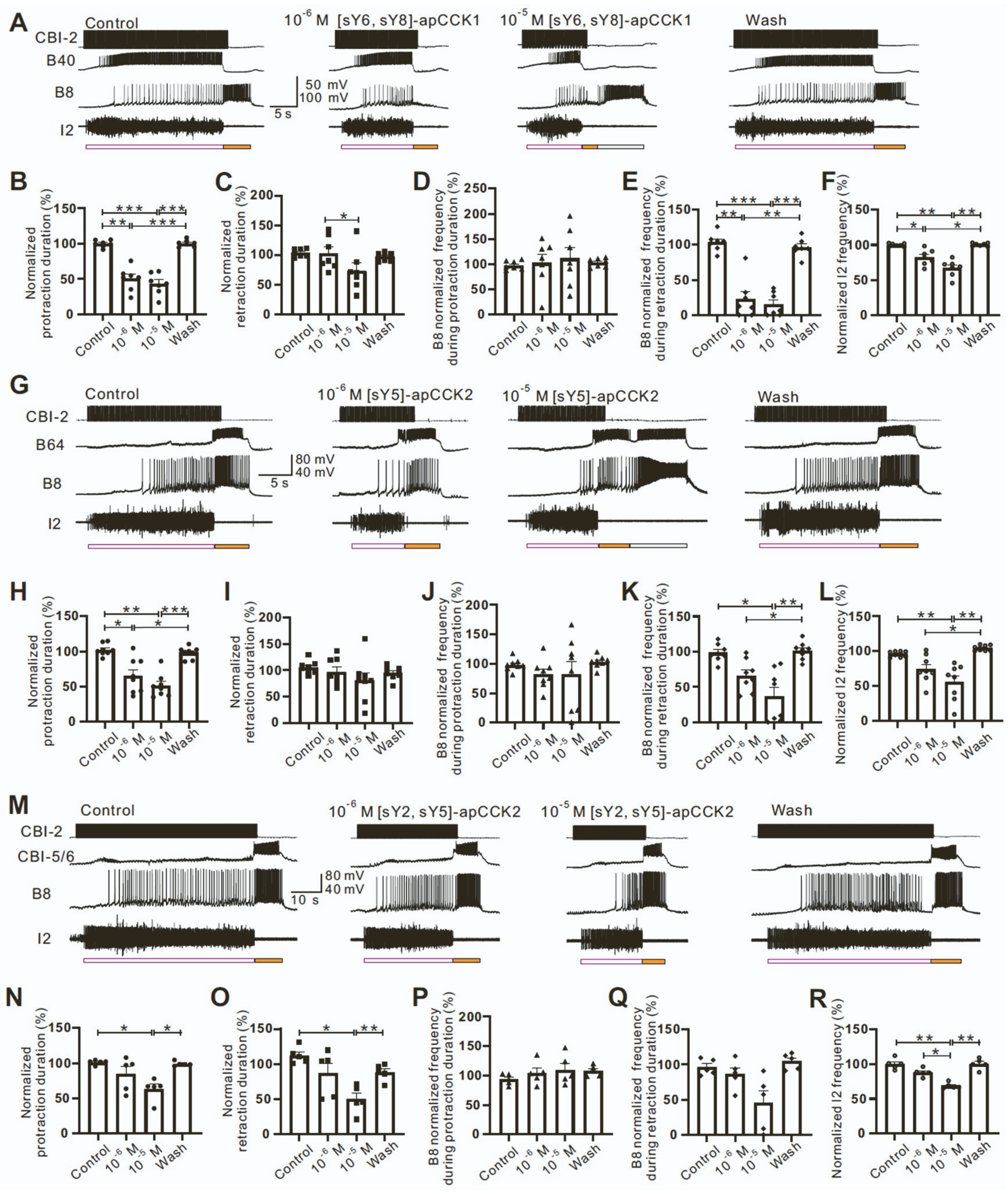

Figure S24: The effects of di-sulfated [sY6, sY8]-apCCK1, mono-sulfated [sY5]-apCCK2, and di-sulfated [sY2, sY5]-apCCK2 on feeding motor programs elicited by CBI-2. Related to Figure 5. A single cycle of a motor program was elicited by stimulating CBI-2 until the end of the protraction phase, represented by the onset of the sharp synaptic inhibition of CPG neuron B40 or synaptic excitation of CPG neuron B64 or CBI-5/6 and the abrupt ending of I2 nerve activity. Protraction phase (open bar) is defined by the activity in the I2 nerve. Retraction phase (filled bar) is defined by a period of hyperpolarization of B40 or depolarization of B64 or CBI-5/6 after the protraction phase. Upon wash, protraction duration and retraction duration return to their control values. **(A-F)** [sY6, sY8]-apCCK1 shortened

protraction duration **(B)** (one-way ANOVA, F (3, 18) = 55.99, $P < 0.0001$) in a concentration-dependent manner, while it had no significant effect on retraction duration **(C)** (one-way ANOVA, F (3, 18) = 3.646, $P = 0.0802$). In addition, [sY6, sY8]-apCCK1 had no significant effect on B8 activity during protraction phase **(D)** (one-way ANOVA, F (3, 18) = 0.2233, $P = 0.7531$), while it reduced B8 activity during retraction phase **(E)** (one-way ANOVA, F (3, 18) = 41.44, $P < 0.0001$). Furthermore, [sY6, sY8]-apCCK1 reduced I2 frequency **(F)** (one-way ANOVA, F (3, 18) = 28.63, $P < 0.0001$). **(A)** Representative example; **(B-F)** Group data. **(G-L)** [sY5]-apCCK2 shortened protraction duration in a concentration-dependent manner **(H)** (one-way ANOVA, F (3, 21) = 20.51, $P < 0.0001$), while it had no significant effect on retraction duration **(I)** (one-way ANOVA, F (3, 21) = 1.349, $P = 0.2907$). In addition, [sY5]-apCCK2 had no significant effect on B8 activity during protraction phase **(J)** (one-way ANOVA, F (3, 21) = 0.9194, $P = 0.3976$), while it reduced B8 activity during retraction phase **(K)** (one-way ANOVA, F (3, 21) = 15.74, $P = 0.0002$). Furthermore, [sY5]-apCCK2 reduced I2 frequency **(L)** (one-way ANOVA, F (3, 21) = 17.65, $P = 0.0006$). **(G)** Representative example; **(H-L)** Group data. **(M-R)** [sY2, sY5]-apCCK2 shortened protraction duration **(N)** (one-way ANOVA, F (3, 12) = 8.51, $P = 0.0156$) and retraction duration **(O)** (one-way ANOVA, F (3, 12) = 6.966, $P = 0.0307$) in a concentration-dependent manner. In addition, [sY2, sY5]-apCCK2 had no significant effect on B8 activity during protraction phase **(P)** (one-way ANOVA, F (3, 12) = 0.7423, $P = 0.4865$), while it weakly reduced B8 activity during retraction phase without significant effect **(Q)** (one-way ANOVA, F (3, 12) = 6.138, $P = 0.0276$). Furthermore, [sY2, sY5]-apCCK2 significantly reduced I2 frequency **(R)** (one-way ANOVA, F (3, 12) = 20.51, $P = 0.0024$). **(M)** Representative example; **(N-R)** Group data. Tukey post hoc test: * $P < 0.05$; ** $P < 0.01$; *** $P < 0.001$; Error bar: SEM.

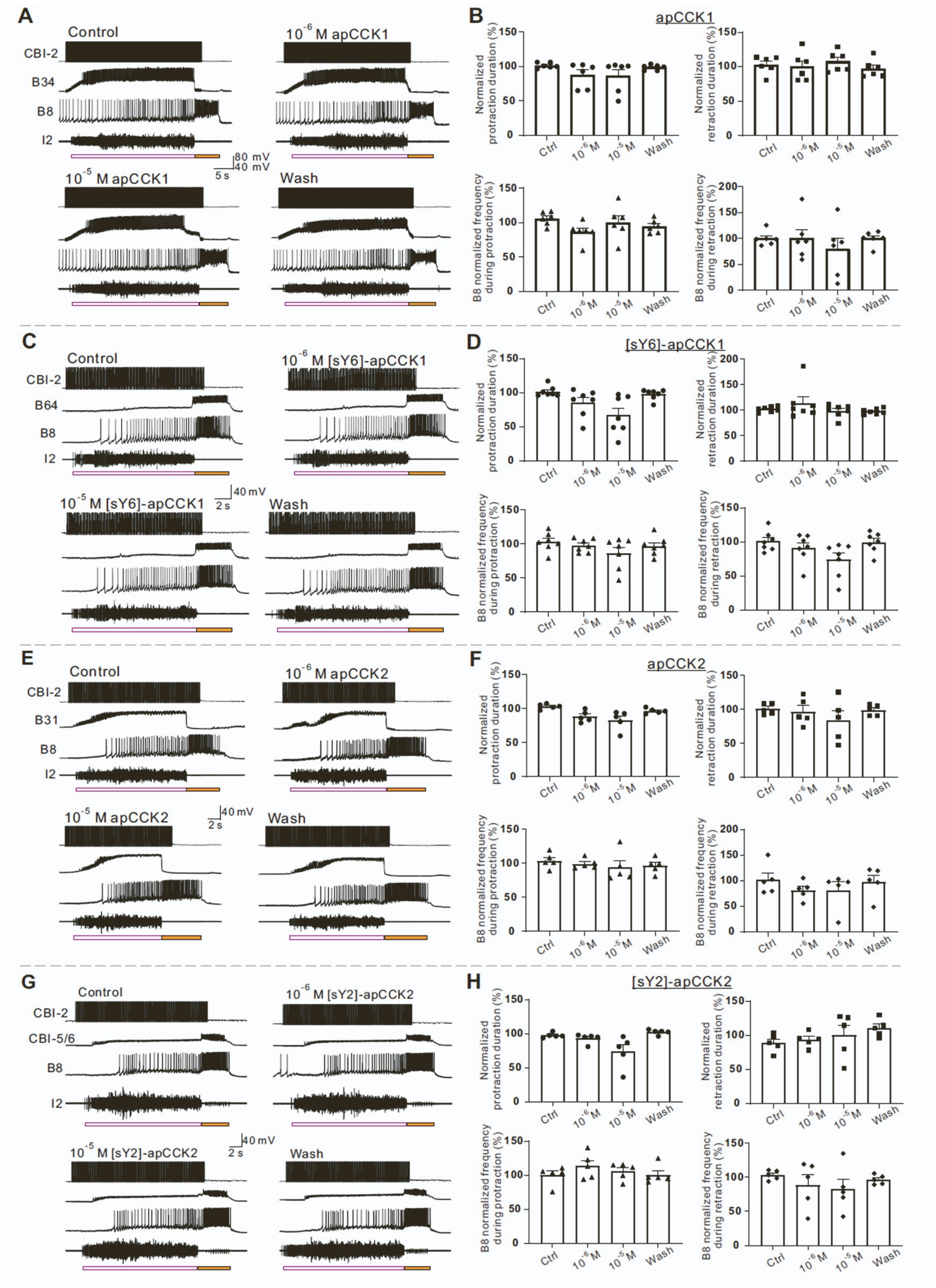

Figure S25: apCCKs with non-sulfated and mono-sulfated tyrosine located toward the N-terminus had no significant effect on feeding motor programs elicited by CBI-2. Related to Figure 5. (A, B) apCCK1. **(A)** Representative example; **(B)** Group data. Protraction duration: one-way ANOVA, F (3, 15) = 2.268, *P* = 0.1841; retraction duration: one-way ANOVA, F (3, 15) = 0.4704, *P* = 0.6491; B8

activity during protraction: one-way ANOVA, $F(3, 15) = 2.037$, $P = 0.1963$; B8 activity during retraction: one-way ANOVA, $F(3, 15) = 0.6433$, $P = 0.5442$. **(C, D)** [sY6]-apCCK1. (**C**) Representative example; **(D)** Group data. Protraction duration: one-way ANOVA, $F(3, 18) = 6.264$, $P = 0.0129$; retraction duration: one-way ANOVA, $F(3, 18) = 1.133$, $P = 0.3422$; B8 activity during protraction: one-way ANOVA, $F(3, 18) = 1.201$, $P = 0.3294$; B8 activity during retraction: one-way ANOVA, $F(3, 18) = 3.107$, $P = 0.1021$. **(E, F)** apCCK2. **(E)** Representative example; **(F)** Group data. Protraction duration: one-way ANOVA, $F(3, 12) = 6.127$, $P = 0.0328$; retraction duration: one-way ANOVA, $F(3, 12) = 0.9616$, $P = 0.4093$; B8 activity during protraction: one-way ANOVA, $F(3, 12) = 0.3811$, $P = 0.6094$; B8 activity during retraction: one-way ANOVA, $F(3, 12) = 0.6232$, $P = 0.4884$. **(G, H)** [sY2]-apCCK2. **(G)** Representative example; **(H)** Group data. Protraction duration: one-way ANOVA, $F(3, 12) = 4.872$, $P = 0.0781$; retraction duration: one-way ANOVA, $F(3, 12) = 1.093$, $P = 0.3788$; B8 activity during protraction: one-way ANOVA, $F(3, 12) = 0.7065$, $P = 0.4771$; B8 activity during retraction: one-way ANOVA, $F(3, 12) = 0.8565$, $P = 0.4513$. Error bar: SEM.

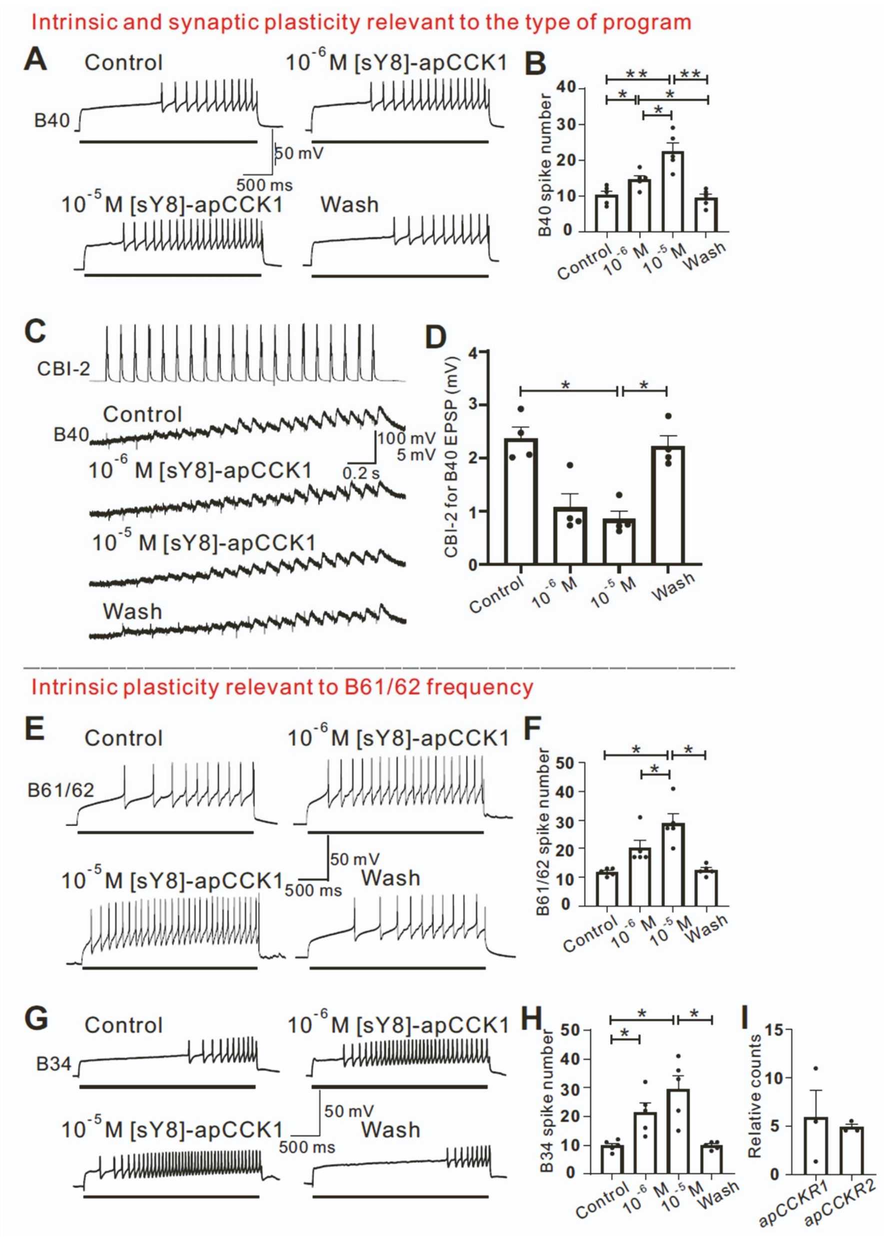

Figure S26: The effect of [sY8]-apCCK1 on the intrinsic and synaptic plasticity of interneurons B40, B61/62 and B34. Related to Figure 6. (**A, B**) [sY8]-apCCK1 enhanced the excitability of B40 neuron. **(A)** Representative examples; **(B)** Group data. One-way ANOVA, F (3, 12) = 51.17, *P* = 0.0002. **(C, D)** [sY8]-apCCK1 decreased the synaptic strength between CBI-2 and B40. **(C)** Representative examples; **(D)** Group data. One-way ANOVA, F (3, 9) = 20.65, *P* = 0.0155. **(E, F)** [sY8]-apCCK1 enhanced the excitability of B61/62 neuron. **(E)** Representative examples; **(F)** Group data. One-way ANOVA, F (3, 12) = 23.44, *P* = 0.0044. **(G, H)** [sY8]-apCCK1 enhanced the excitability of B34 neuron. **(G)** Representative examples; **(H)** Group data. One-way ANOVA, F (3, 12) = 18.19, *P* = 0.0087. **(I)** Smart single cell sequencing showed the expression of *apCCKR1* and *apCCKR2* gene in B34 neurons. Relative counts are obtained by removing batch effects. Tukey post hoc test: **P* < 0.05; ***P* < 0.01. Error bar: SEM.

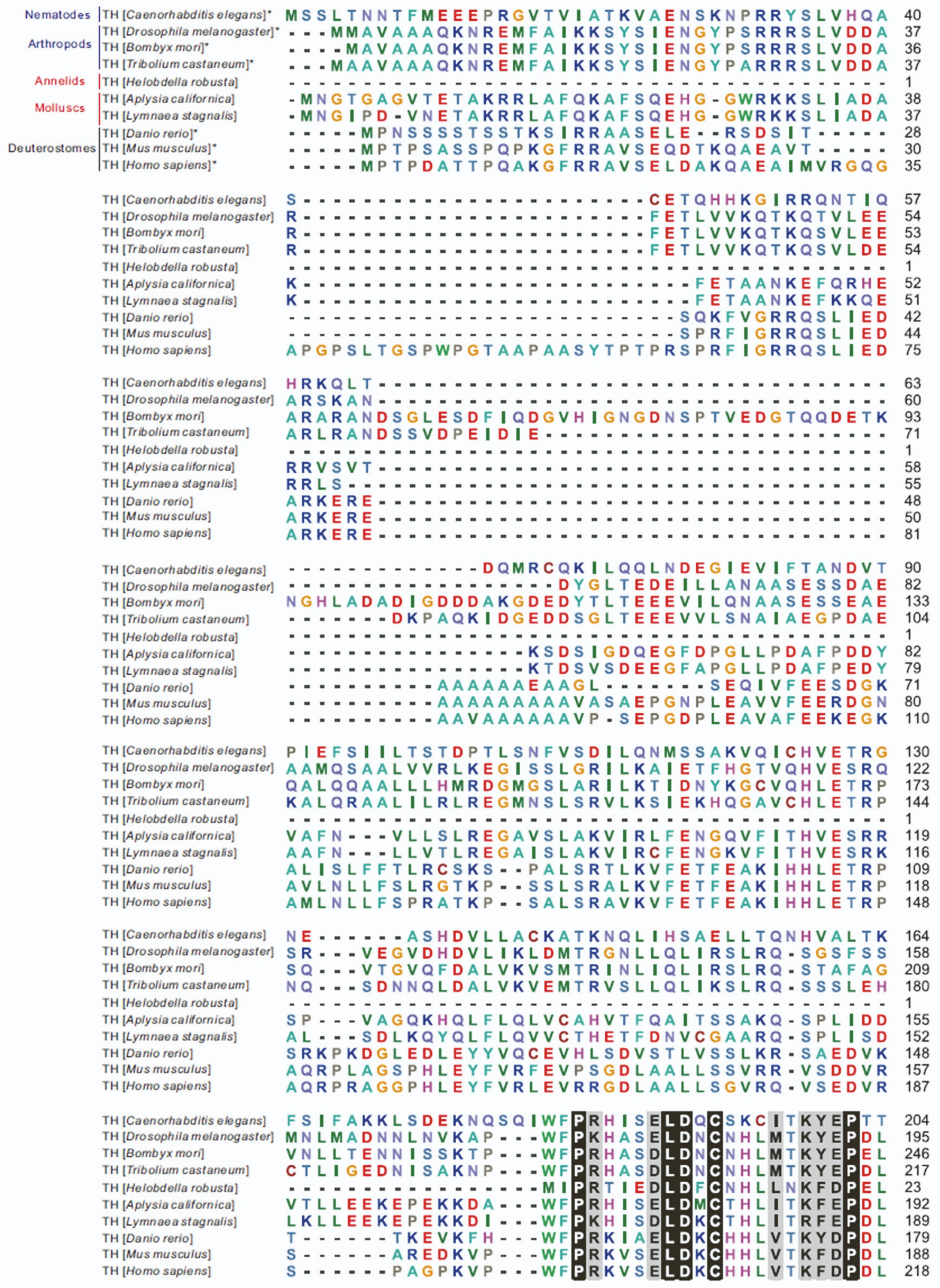
Nematodes
Arthropods
Annelids
Molluscs
Deuterostomes
TH [Caenorhabditis elegans]*
TH [Drosophila melanogaster]*
TH [Bombyx mori]*
TH [Tribolium castaneum]*
TH [Helobdella robusta]
TH [Aplysia californica]
TH [Lymnaea stagnalis]
TH [Danio rerio]*
TH [Mus musculus]*
TH [Homo sapiens]*

(page 1)

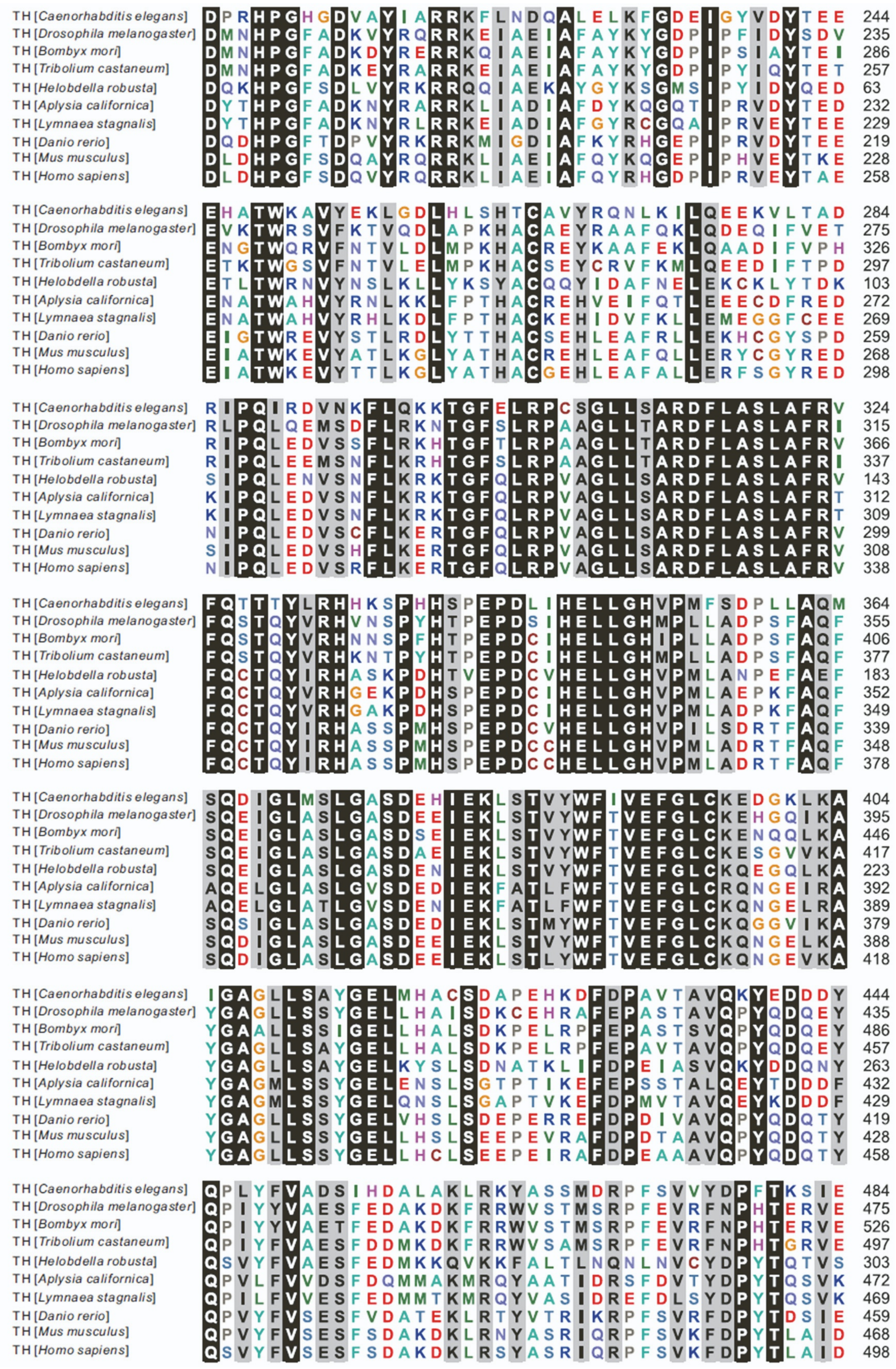
TH [Caenorhabditis elegans] DPRHPGHGDVAYIARRKFLNDQALELKFGDEIGYVDYTEE 244
TH [Drosophila melanogaster] DMNHPGFADKVYRQRRKEIAEIAFAYKYGDPIPFIDYSDV 235
TH [Bombyx mori] DMNHPGFADKDYRERRKQIAEIAFAYKYGDPIPSIAYTEI 286
TH [Tribolium castaneum] DMNHPGFADKEYRARRKEIAEIAFAYKYGDPIPYIQYTET 257
TH [Helobdella robusta] DQKHPGFSDLVYRKRRQQIAEKAYGYKSGMSIPYIDYQED 63
TH [Aplysia californica] DYTHPGFADKNYRARRKLIADIAFDYKQGQTIPRVDYTED 232
TH [Lymnaea stagnalis] DYTHPGFADKNYRLRRKEIADIAFGYRCGQAIPRVEYTEE 229
TH [Danio rerio] DQDHPGFTDPVYRKRRKMIGDIAFKYRHGEPIPRVDYTEE 219
TH [Mus musculus] DLDHPGFSDQAYRQRRKLIAEIAFQYKQGEPIPHVEYTKE 228
TH [Homo sapiens] DLDHPGFSDQVYRQRRKLIAEIAFQYRHGDPIPRVEYTAE 258
TH [Caenorhabditis elegans] EHATWKAVYEKLGDLHLSHTCAVYRQNLKILQEEKVLTAD 284
TH [Drosophila melanogaster] EVKTWRSVFKTVQDLAPKHACAEYRAAFQKLQDEQIFVET 275
TH [Bombyx mori] ENGTWQRVFNTVLDLMPKHACREYKAAFEKLQAADIFVPH 326
TH [Tribolium castaneum] ETKTWGSVFNTVLELMPKHACSEYCRVFKMLQEEDIFTPD 297
TH [Helobdella robusta] ETLTWRNVYNSLKLLYKSYACQQYIDAFNELEKCKLYTDK 103
TH [Aplysia californica] ENATWAHVYRNLKKLFPTHACREHVEIFQTLEEECDFRED 272
TH [Lymnaea stagnalis] ENATWAHVYRHLKDLFPTHACKEHIDVFKLLEMEGGFCEE 269
TH [Danio rerio] EIGTWREVYSTLRDLYTTHACSEHLEAFRLLEKHCGYSPD 259
TH [Mus musculus] EIATWKEVYATLKGLYATHACREHLEAFQLLERYCGYRED 268
TH [Homo sapiens] EIATWKEVYTTLKGLYATHACGEHLEAFALLERFSGYRED 298
TH [Caenorhabditis elegans] RIPQIRDVNKFLQKKTGFELRPCSGLLSARDFLASLAFRV 324
TH [Drosophila melanogaster] RLPQLQEMSDFLRKNTGFSLRPAAGLLTARDFLASLAFRI 315
TH [Bombyx mori] RIPQLEDVSSFLRKHTGFTLRPAAGLLTARDFLASLAFRV 366
TH [Tribolium castaneum] RIPQLEEMSNFLKRHTGFSLRPAAGLLTARDFLASLAFRI 337
TH [Helobdella robusta] SIPQLENVSNFLKRKTGFQLRPVAGLLSARDFLASLAFRV 143
TH [Aplysia californica] KIPQLEDVSNFLKRKTGFQLRPVAGLLSARDFLASLAFRT 312
TH [Lymnaea stagnalis] KIPQLEDVSNFLKRKTGFQLRPVAGLLSARDFLASLAFRT 309
TH [Danio rerio] NIPQLEDVSCFLKERTGFQLRPVAGLLSARDFLASLAFRV 299
TH [Mus musculus] SIPQLEDVSHFLKERTGFQLRPVAGLLSARDFLASLAFRV 308
TH [Homo sapiens] NIPQLEDVSRFLKERTGFQLRPVAGLLSARDFLASLAFRV 338
TH [Caenorhabditis elegans] FQTTTYLRHHKSPHHSPEPDLIHELLGHVPMFSDPLLAQM 364
TH [Drosophila melanogaster] FQSTQYVRHVNSPYHTPEPDSIHELLGHMPLLADPSFAQF 355
TH [Bombyx mori] FQSTQYVRHNNSPFHTPEPDCIHELLGHIPLLADPSFAQF 406
TH [Tribolium castaneum] FQSTQYVRHKNTPYHTPEPDCIHELLGHMPLLADPSFAQF 377
TH [Helobdella robusta] FQCTQYIRHASKPDHTVEPDCVHELLGHVPMLANPEFAEF 183
TH [Aplysia californica] FQCTQYVRHGEKPDHSPEPDCIHELLGHVPMLAEPKFAQF 352
TH [Lymnaea stagnalis] FQCTQYVRHGAKPDHSPEPDCIHELLGHVPMLADPKFAQF 349
TH [Danio rerio] FQCTQYIRHASSPMHSPEPDCVHELLGHVPILSDRTFAQF 339
TH [Mus musculus] FQCTQYIRHASSPMHSPEPDCCHELLGHVPMLADRTFAQF 348
TH [Homo sapiens] FQCTQYIRHASSPMHSPEPDCCHELLGHVPMLADRTFAQF 378
TH [Caenorhabditis elegans] SQDIGLMSLGASDEHIEKLSTVYWFIVEFGLCKEDGKLKA 404
TH [Drosophila melanogaster] SQEIGLASLGASDEEIEKLSTVYWFTVEFGLCKEHGQIKA 395
TH [Bombyx mori] SQEIGLASLGASDSEIEKLSTVYWFTVEFGLCKENQQLKA 446
TH [Tribolium castaneum] SQEIGLASLGASDAEIEKLSTVYWFTVEFGLCKESGVVKA 417
TH [Helobdella robusta] SQEIGLASLGASDENIEKLSTVYWFTVEFGLCKQEGQLKA 223
TH [Aplysia californica] AQELGLASLGVSDEDIEKFATLFWFTVEFGLCRQNGEIRA 392
TH [Lymnaea stagnalis] AQELGLATLGVSDENIEKFATLFWFTVEFGLCKQNGELRA 389
TH [Danio rerio] SQSIGLASLGASDEDIEKLSTMYWFTVEFGLCKQGGVIKA 379
TH [Mus musculus] SQDIGLASLGASDEEIEKLSTVYWFTVEFGLCKQNGELKA 388
TH [Homo sapiens] SQDIGLASLGASDEEIEKLSTLYWFTVEFGLCKQNGEVKA 418
TH [Caenorhabditis elegans] IGAGLLSAYGELMHACSDAPEHKDFDPAVTAVQKYEDDDY 444
TH [Drosophila melanogaster] YGAGLLSSYGELLHAISDKCEHRAFEPASTAVQPYQDQEY 435
TH [Bombyx mori] YGAALLSSIGELLHALSDKPELRPFEPASTSVQPYQDQEY 486
TH [Tribolium castaneum] YGAGLLSAYGELLHALSDKPELRPFEPAVTAVQPYQDQEY 457
TH [Helobdella robusta] YGAGLLSAYGELKYSLSDNATKLIFDPEIASVQKYDDQNY 263
TH [Aplysia californica] YGAGMLSSYGELENSLSGTPTIKEFEPSSTALQEYTDDDF 432
TH [Lymnaea stagnalis] YGAGMLSSYGELQNSLSGAPTVKEFDPMVTAVQEYKDDDF 429
TH [Danio rerio] YGAGLLSSYGELVHSLSDEPERREFDPDIVAVQPYQDQTY 419
TH [Mus musculus] YGAGLLSSYGELLHSLSEEPEVRAFDPDTAAVQPYQDQTY 428
TH [Homo sapiens] YGAGLLSSYGELLHCLSEEPEIRAFDPEAAAVQPYQDQTY 458
TH [Caenorhabditis elegans] QPLYFVADSIHDALAKLRKYASSMDRPFSVVYDPFTKSIE 484
TH [Drosophila melanogaster] QPIYYVAESFEDAKDKFRRWVSTMSRPFEVRFNPHTERVE 475
TH [Bombyx mori] QPIYYVAETFEDAKDKFRRWVSTMSRPFEVRFNPHTERVE 526
TH [Tribolium castaneum] QPIYFVAESFDDMKDKFRRWVSAMSRPFEVRFNPHTGRVE 497
TH [Helobdella robusta] QSVYFVAESFEDMKKQVKKFALTLNQNLNVCYDPYTQTVS 303
TH [Aplysia californica] QPVLFVVDSFDQMMAKMRQYAATIDRSFDVTYDPYTQSVK 472
TH [Lymnaea stagnalis] QPILFVVESFEDMMTKMRQYVASIDREFDLSYDPYTQSVK 469
TH [Danio rerio] QPVYFVSESFVDATEKLRTYVTRIKRPFSVRFDPYTDSIE 459
TH [Mus musculus] QPVYFVSESFSDAKDKLRNYASRIQRPFSVKFDPYTLAID 468
TH [Homo sapiens] QSVYFVSESFSDAKDKLRSYASRIQRPFSVKFDPYTLAID 498

(page 2)

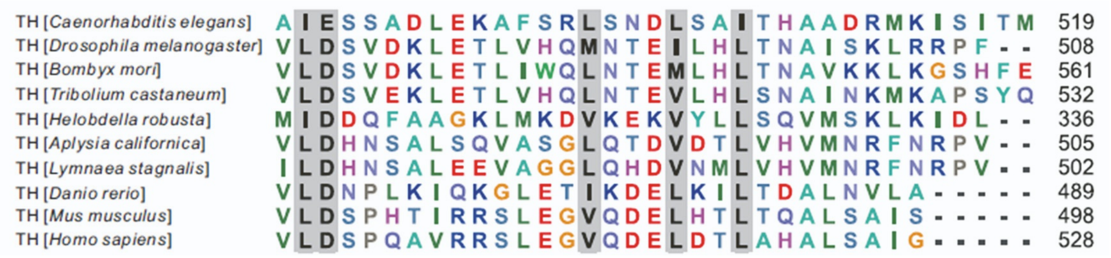

(page 3)

Figure S27: The alignment of tyrosine hydroxylase (TH) from several protostomes including nematodes (*Caenorhabditis elegans*), arthropods (*Drosophila melanogaster, Bombyx mori, Tribolium castaneum*), annelids (*Helobdella robusta*), mollusks (*Aplysia californica, Lymnaea stagnalis*), and several deuterostomes (*Danio rerio*, *Mus musculus*, *Homo sapiens*) using BioEdit (ClustalW Multiple Alignment-Graphic view). Related to Figure 6. *: the THs that have been identified.

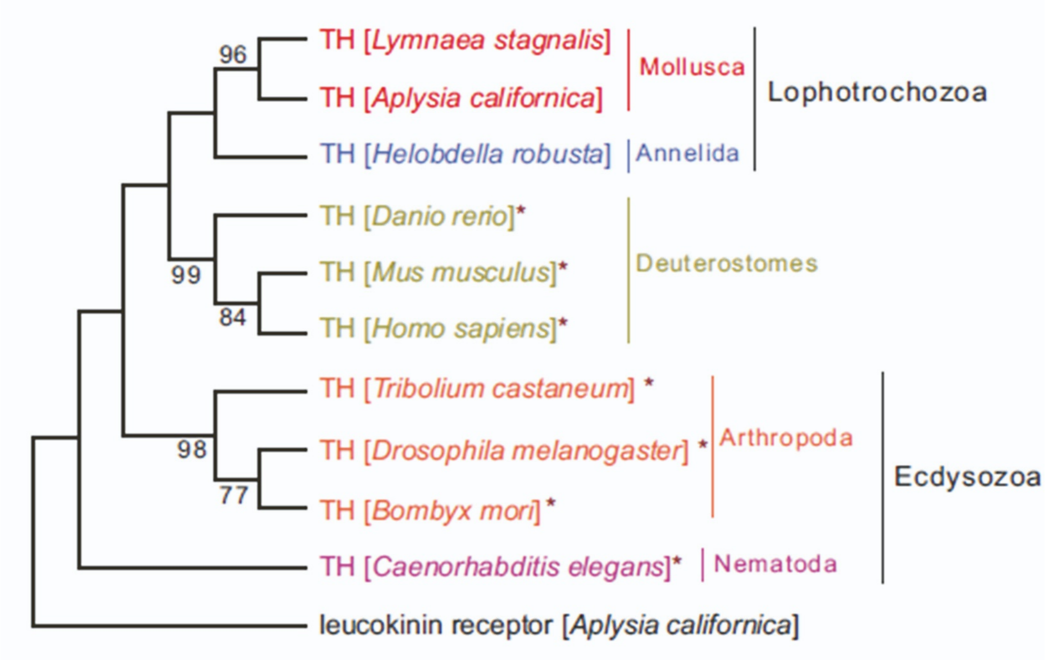

Figure S28: A phylogenetic tree of TH in *Aplysia* with predicted or verified TH in protostomes (including Lophotrochozoa and Ecdysozoa) and deuterostomes. Related to Figure 6. The tree was generated using LG + G model in MEGA X with 1000 replicates (See Dataset S9 for information on the sequences). This phylogenetic tree indicated that the *Aplysia* TH were more closely related to the TH sequences in molluscan *Lymnaea stagnalis*. * indicates that the TH has been verified. "leucokinin receptor" in *Aplysia californica* is used as an outgroup. Note that lophotrochozoan THs cluster with deuterostome THs rather than ecdysozoa THs. Numbers at the nodes are bootstrap values as percentage. Only bootstrap values greater than 50 are shown.

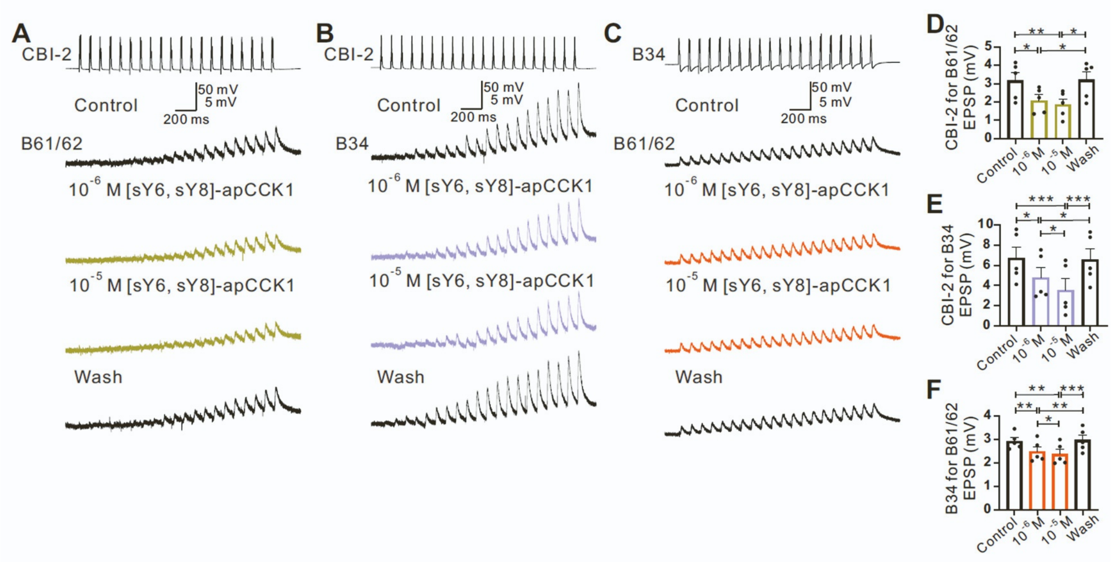

Figure S29: The effect of [sY6, sY8]-apCCK1 on the synaptic strength underlying modulation of protraction activity in feeding programs. Related to Figure 6. (A, D) [sY6, sY8]-apCCK1 reduced the synaptic strength between CBI-2 and B61/62. **(A)** Representative example. **(D)** Group data. One-way ANOVA, $F(3, 12) = 35.17$, $P = 0.0014$. **(B, E)** [sY6, sY8]-apCCK1 reduced the synaptic strength between CBI-2 and B34. **(B)** Representative example. **(E)** Group data. One-way ANOVA, $F(3, 12) = 83.05$, $P = 0.0001$. **(C, F)** [sY6, sY8]-apCCK1 reduced the synaptic strength between B34 and B61/62. **(C)** Representative example. **(F)** Group data. One-way ANOVA, $F(3, 12) = 57.71$, $P < 0.0001$. Tukey post hoc test: $^{*}P < 0.05$; $^{**}P < 0.01$; $^{***}P < 0.001$. Error bar: SEM.

Table S1. Effects of 8 forms of *Aplysia* CCKs on feeding behavior (food intake), CCK receptors (apCCKR1 and apCCKR2), and feeding motor programs. Related to Figure 2, Figure 3 and Figure 5.

Peptides	Behavioral effect	apCCKR1 (EC_{50})	apCCKR2 (EC_{50})	Feeding program effect
[sY8]-apCCK1	Strongest	22 nM	42 nM	Strongest
[sY6, sY8]-apCCK1	Strong	7 nM	640 nM	Strong
[sY5]-apCCK2	Moderate	850 nM	49 nM	Moderate
[sY2, sY5]-apCCK2	Weak	330 nM	370 nM	Weak
[sY6]-apCCK1	No effect	1800 nM	5900 nM	No effect
apCCK1	No effect	5600 nM	> 10000 nM	No effect
[sY2]-apCCK2	No effect	> 10000 nM	1400 nM	No effect
apCCK2	No effect	> 10000 nM	> 10000 nM	No effect
